# Health issues and needs among adolescents in Ethiopia: A cross-sectional study

**DOI:** 10.1371/journal.pgph.0006496

**Published:** 2026-06-01

**Authors:** Adamu Amanu Asari, Ameyu Godesso, Zewdie Birhanu

**Affiliations:** 1 Department of Health, Behavior, and Society, Faculty of Public Health, Jimma University, Oromia, Ethiopia; 2 Department of Sociology, College of Social Sciences, Jimma University, Oromia, Ethiopia; University of Sydney, AUSTRALIA

## Abstract

Adolescence, which is a critical foundation for lifelong health, comes with distinct health challenges and needs. Understanding these is crucial for timely and effective measures. This study therefore examines adolescent health issues and needs and identifies unmet health information and service needs, including differences by sex, grade level, and school type, to inform tailored and effective interventions. The study employed a cross-sectional design involving school adolescents. Respondents were randomly selected after stratification by sex at grade level. Data were collected using a self-administered questionnaire that was informed by qualitative findings. Descriptive statistics were used to summarize the data, and Pearson’s chi-square tests evaluated differences by sex, grade level, and school type, in SPSS 27.0. Of the 722 adolescents participated in this study, 27.6% reported recent health problems related to mental health, reproductive health, and other issues. Respondents identified risky sexual behaviors, substance use, mental health issues, and social media addiction as major health concerns among adolescents, with sex-based differences in perceived priorities. About 73% of the respondents reported seeking health information and services for various reasons, with females were more likely to do so than males (p = 0.025). They reported often consulting parents, friends, the Internet, and health professionals, in that order, while seeking health information, with variations by school type and grade level. However, they trusted health professionals most, followed by the Internet, television/radio, and family. Only 32.7% of the respondents reported that they could access health service when needed. Adolescents from private schools were significantly more likely than those from public schools to do so (p < 0.001). Overall, the study shows that adolescent health challenges and needs are multifaceted, demanding tailored interventions, including effective multichannel health communication, promotion of health literacy, strong parental and community involvement, and accessible adolescent-friendly health services.

## Background

Adolescents are often presumed to be a healthy group, as they are in a relatively healthy phase of life [1–3]. However, adolescence is a critical developmental period characterized by rapid physical, cognitive, and psychosocial changes that create both opportunities for positive health development and vulnerabilities, often expressed through risk-taking and experimentation [4,5]. This age group often face a range of health-related challenges, including risky sexual behaviors and reproductive health issues, substance use, mental health problems, injuries, school and interpersonal difficulties, unhealthy diets, and others [6–8]. Behaviors and habits established during adolescence have long lasting consequences [1,8,9]. For instance, substance use, risky sexual behaviors, physical inactivity, and other behavioral problems can seriously disrupt adolescent health and development [1,2,7]. Moreover, many serious diseases, particularly chronic conditions and premature mortality in adulthood have their roots in behaviors and lifestyles established during adolescence [9–11].

Therefore, adolescence is a foundational period for lifelong health and wellbeing [7,12] and represents a critical window for disease prevention and health promotion initiatives [1,7,13]. It is an important stage for providing timely and appropriate support for healthy lifestyles, as well as interventions for those engaging in unhealthy behaviors [13,14]. Thus, emphasizing adolescent health issues and ensuring that this age group has sufficient and continuous access to accurate health information and services is a crucial public health concern, especially in developing countries with predominantly young population such as Ethiopia [15]. Doing so helps to have healthy adolescents, raise healthier families and population in the future, reduce the burden of health problems, lower future healthcare costs, and enhance overall human capital and societal development [7,12,16]. Conversely, neglecting adolescent health issues can negatively affect adolescents’ present and future wellbeing and the quality of life of future generations [9,13,17].

Adolescents require health information and services across a wide range of areas, including mental health, sexual and reproductive health, nutrition, self-care, addiction issues, and preventive strategies [18,19]. However, many adolescents, particularly in developing countries, do not have access to adequate, adolescent-friendly health information and services tailored to their specific needs [19–21]. Ethiopia exemplifies this challenge [22–24], although the National Adolescent and Youth Health Strategy recognizes adolescents’ health issues, including reproductive health, nutrition, substance use, mental health, and others, as areas requiring considerations [15]. Due to this problem and as they are still in the process of developing health knowledge and decision-making skills and have limited past experiences, adolescents often depend on unreliable sources and services [25–28]. They may rely on information from friends, siblings, and social media [28–30], which can expose them to inaccuracies and negatively affect their health and well-being [26,31].

Therefore, health planners and providers need to understand adolescents’ health-related challenges, including emerging risks and specific needs [2,6,7,32], to ensure access to appropriate and timely services in order to support healthy development in this age group [19,33]. Accordingly, it is essential to examine adolescents’ health issues, including their health information and service needs, information-seeking behaviors, preferred sources, types of information needed, and access to services, to inform targeted interventions, with effective resource utilizations [19,20,28,33], especially in developing countries like Ethiopia. However, these issues remain under-researched in Ethiopia.

Therefore, as part of a larger research project on adolescent health literacy and needs in Ethiopia [16,34,35], this study focuses on adolescents’ health issues and needs. Specifically, it (i) assesses adolescents’ self-perceived health status, recent health problems, treatments used, and access to health services, and (ii) examines their health information-seeking behaviors, including types of information sought, usually consulted and trusted sources, and their perceived major health topics or concerns warranting attention, with the aim of identifying unmet health information and service needs, including differences by sex, grade level, and school type, in order to inform tailored and effective interventions.

## Methods

### Study design and participants

The study employed a cross-sectional design involving school adolescents in Jimma city, home to socio-demographically diverse population in Oromia regional state, Ethiopia. As previously reported in [18,35], there were sixteen high schools in the study area at the time of the study. Of these, eight schools with socio-demographically diverse and representative adolescent populations were included in this study. Among 737 adolescents selected using simple random sampling after stratification by sex at grade level across all the selected schools, 722 participated. This sample size was increased from the initially determined 464 (using Cochran’s formula with a finite population correction, assuming a 95% confidence level, a ± 5% precision level, and a 0.5 population proportion, along with a 25% non-response rate [36–38]) to ensure representation across school types and the accuracy of the results [35,39,40].

### Data collection and analysis

Data collection for this survey was carried out using a questionnaire developed on the basis of qualitative findings [16] and a review of relevant literature, in alignment with the study’s objectives. The questionnaire comprised multiple choice questions, most allowing multiple responses. This questionnaire begins with items on respondents’ sociodemographic characteristics, including sex, age, grade level, school type, and others. It then follows with questions on self-perceived overall health status, recent health experience (and types), treatments used for management or recovery, and access to health services. Finally, it focuses on the health topics the target groups might have been exposed to or have learned so far (including the sources of such topics or information), their health information-seeking behaviors (encompassing reasons, specific topics sought, sources they usually turn to, and trusted sources), and their perceived major health topics or concerns that warrant attention (See Supporting Information, S1 File). Prior to the actual data collection, the questionnaire was pretested to ensure the clarity, content quality, cognitive appropriateness, comprehensibility, and overall usability of the items or questions [41].

The questionnaire was administered in paper format in designated spaces, either within classrooms or nearby locations, to the recruited, consenting, and eligible adolescents (aged 19 years or younger). Data collection schedules were arranged with each school’s director. Data were collected between January 8 and February 27, 2024. The study was conducted in Afaan Oromo and Amharic languages based on the participants’ preferences.

The collected data were entered into Epidata 3.1 software and cleaned, and then transferred to the statistical package SPSS 27.0 to perform the analysis. Descriptive statistics were used to summarize the data, and Pearson’s chi-square tests were used to evaluate differences based on sex, grade level, and school type, and in cases where expected frequencies were less than five, Fisher-Freeman-Halton Exact Test was also applied with chi-square test to ensure accuracy of the results, with statistical significance of p < 0.05.

### Ethics approval and informed consent

The study was reviewed and approved by the Institutional Review Board (IRB) of Institute of Health at Jimma University (Ref. No. JUIH/IRB/321/23), and it adhered to the ethical principles of the Declaration of Helsinki [42]. Having obtained ethical clearance from the IRB, the researcher (the first author) made an official request to each school director, with a support letter from the Department of Health, Behavior, and Society, Faculty of Public Health, Jimma University, for the necessary facilitation and smooth conduct of the study. Participation in the study was fully voluntary. Informed consent was obtained from participants of age 18 and above after explaining the purpose and confidentiality of the study, and for those aged less than 18 years, informed consent was obtained from their parents or guardians before getting assent from this age group.

## Results

### Sociodemographic characteristics

In total, seven hundred twenty-two adolescents aged 14–19 participated in this study (mean age = 16.75, SD = 1.284, and mode = 16). As previously reported [18,35], more than half (56.40%) of the respondents were female. Most respondents (78.9%) were from urban background, and 58.2% attended public schools. In terms of grade level, 53.6% were in grades 9–10, while the remaining 46.4% were in grades 11–12. Concerning parents’ educational levels, 54.8% of fathers had low education and 45.2% had high education. While 59.8% of mothers had low education and 40.2% had high education. Thus, overall, fathers tend to have slightly higher levels of education than mothers. Regarding household average monthly income, 48.9%, 25.9%, and 25.2% reported it as low, middle, and high income, respectively.

### Self-perceived health status, recent health problems, treatments used, and access to health services

Self-perceived overall health status ranged from good to excellent for about 89.5% of the respondents. However, participants who reported sometimes, often, or everyday experiencing health problems recently, accounted for about 27.6% of the total sample (See Table 1). Among those who reported experiencing health problems recently, 50.4% reported mental health-related problems; 11.9% reproductive health-related problems; 18.9% sleep difficulty, and 18.5% other health problems (such as gastric/stomach problem, malaria, typhoid, toothache, eyes problem, lack of appetite, and asthma/sinus). Chi-square test showed that female respondents were more likely than male respondents to report experiencing mental health problems (p = 0.008).

**Table 1:** Self-perceived health status, recent health problems, treatments used, and access to health services by sex, grade level, and school type.

Questions	Responses	Sex			Grade level			School type			Total n (%)
		Male n (%)	Female n (%)	P-value	9-10 n (%)	11-12 n (%)	P-value	Public n (%)	Private n (%)	P-value	
Self- perceived health status	Excellent	139 (44.10%)	180 (44.20%)	0.788 0.795 ^F^	177 (45.70%)	142 (42.40%)	0.387 0.395 ^F^	177 (42.10%)	142 (47.00%)	0.122 0.119 ^F^	319 (44.20%)
	Very good	107 (34.00%)	124 (30.50%)		120 (31.00%)	111 (33.10%)		144 (34.30%)	87 (28.80%)		231 (32.00%)
	Good	36 (11.40%)	60 (14.70%)		52 (13.40%)	44 (13.10%)		62 (14.80%)	34 (11.30%)		96 (13.30%)
	Fair	24 (7.60%)	30 (7.40%)		23 (5.90%)	31 (9.30%)		27 (6.40%)	27 (8.90%)		54 (7.50%)
	Poor	7 (2.20%)	9 (2.20%)		11 (2.80%)	5 (1.50%)		6 (1.40%)	10 (3.30%)		16 (2.20%)
	Very poor	2 (0.60%)	4 (1.00%)		4 (1.00%)	2 (0.60%)		4 (1.00%)	2 (0.70%)		6 (0.80%)
Experiencing health problem recently	Never	159 (50.50%)	189 (46.40%)	0.093 0.093 ^F^	194 (50.10%)	154 (46.00%)	0.639 0.632 ^F^	210 (50.00%)	138 (45.70%)	0.351 0.370 ^F^	348 (48.20%)
	Rarely	85 (27.00%)	90 (22.10%)		86 (22.20%)	89 (26.60%)		101 (24.00%)	74 (24.50%)		175 (24.20%)
	Sometimes	59 (18.70%)	104 (25.60%)		89 (23.00%)	74 (22.10%)		92 (21.90%)	71 (23.50%)		163 (22.60%)
	Often	11 (3.50%)	20 (4.90%)		15 (3.90%)	16 (4.80%)		16 (3.80%)	15 (5.00%)		31 (4.30%)
	Everyday	1 (0.30%)	4 (1.00%)		3 (0.80%)	2 (0.60%)		1 (0.20%)	4 (1.30%)		5 (0.70%)
Health problem type	Mental health related	66 (42.30%)	123 (56.20%)	0.008	94 (48.70%)	95 (52.20%)	0.499	98 (46.40%)	91 (55.50%)	0.082	189 (50.40%)
	Reproductive health related	15 (9.60%)	30 (13.60%)	0.243	29 (15.00%)	16 (8.70%)	0.058	26 (12.20%)	19 (11.60%)	0.854	45 (11.90%)
	Sleep difficulty	36 (23.10%)	35 (16.00%)	0.084	34 (17.60%)	37 (20.30%)	0.503	47 (22.30%)	24 (14.60%)	0.061	71 (18.90%)
	Other										69 (18.50%)
Treatments used	Went to a health facility	28 (17.9%)	55 (25.2%)	0.095	44 (22.8%)	39 (21.5%)	0.771	42 (20.0%)	41 (25.0%)	0.248	83 (22.20%)
	Bought medications from a pharmacy	13 (8.3%)	16 (7.3%)	0.723	12 (6.2%)	17 (9.4%)	0.251	18 (8.6%)	11 (6.7%)	0.504	29 (7.80%)
	Used traditional medications	9 (5.8%)	14 (6.4%)	0.796	14 (7.3%)	9 (5.0%)	0.359	16 (7.6%)	7 (4.3%)	0.181	23 (6.10%)
	Used religious healing practices	33 (21.2%)	65 (29.8%)	0.060	57 (29.5%)	41 (22.7%)	0.130	57 (27.1%)	41 (25.0%)	0.640	98 (26.20%)
	Other										141 (37.7%)
Access to health services	Yes, when needed	90 (28.60%)	146 (35.90%)	0.098	143 (37.00%)	93 (27.80%)	0.031	110 (26.20%)	126 (41.70%)	< 0.001	236 (32.70%)
	Yes, to some extent	142 (45.10%)	158 (38.80%)		152 (39.30%)	148 (44.20%)		191 (45.50%)	109 (36.10%)		300 (41.60%)
	No	83 (26.30%)	103 (25.30%)		92 (23.80%)	94 (28.10%)		119 (28.30%)	67 (22.20%)		186 (25.80%)

^F^indicates Fisher-Freeman-Halton Exact Test.

To get treatment and manage the health problems they encountered recently, 22.2% visited health facility (hospital, clinic, or health center); 7.8% purchased medications from a pharmacy (without a prescription); 6.1% used traditional medications; 26.2% used religious healing practices (including prayers and reading holy books privately); and 37.7% others (such as exercise, lying down and rest, and drinking alcohol to forget or avoid it). Regarding access to health services, 32.7%, 41.6%, and 25.8% of the respondents reported that they could access service when needed, could access it to some extent, and could not access it, respectively. Chi-square test showed a significant association between school type and access to health services, with a higher proportion of respondents from private schools reporting access when needed compared to respondents from public schools (p < 0.001).

### Health topics learned, information-seeking behaviors, and trusted sources

Regarding health topics the target group have been exposed to or have learned so far, 89.2%, 69.1%, 41.8%, 39.5%, and 36.8%, reported learning about hygiene and sanitation, sexual and reproductive health-related matters, the health benefits of physical activity, nutrition, and smoking issues, respectively. Other topics learned included alcohol use issues (34.3%), mental health (31.5%), and oral health (22.1%). There were also respondents who reported receiving information on COVID-19, road traffic issues, primary healthcare, eye care, and the health of internal organs such as the lung, kidney, and heart. Respondents from private schools were more likely than those from public schools to report obtaining information on nutrition (p < 0.001), smoking issues (p < 0.001), oral health (p = 0.001), alcohol use issues (p = 0.002), and the health benefits of physical activity (p = 0.025).

As Table 2 shows, their sources of such information included school (96.40%), television/radio (43.90%), the Internet (37.30%), family (35.90%), health professionals (29.50%), religious bodies (27.80%), friends/peers (24.90%), and health clubs (12.80%). In addition, there were also respondents who reported sport rooms or trainers as their sources of health information. More respondents in grades 9–10 than those in grades 11–12 reported family (p = 0.001) and health clubs (p = 0.031) as their sources of such information. Additionally, more respondents from private schools than those from public schools reported family (p < 0.001), health professionals (p = 0.021), the Internet (p < 0.001), and health clubs (p < 0.001) as their sources of such information.

**Table 2:** Health topics learned and sources by sex, grade level, and school type.

Questions	Responses	Sex			Grade level			School type			Total n (%)
		Male n (%)	Female n (%)	P-value	9-10 n (%)	11-12 n (%)	P-value	Public n (%)	Private n (%)	P-value	
Which health information or topic have you got or learned about?	Sexual and reproductive health	215 (68.30%)	284 (69.80%)	0.660	272 (70.30%)	227 (67.80%)	0.464	285 (67.90%)	214 (70.90%)	0.389	499 (69.10%)
	Hygiene and sanitation	266 (84.40%)	378 (92.90%)	<0.001	338 (87.30%)	306 (91.30%)	0.084	372 (88.60%)	272 (90.10%)	0.523	644 (89.20%)
	Mental health	97 (30.90%)	130 (31.90%)	0.764	114 (29.50%)	113 (33.80%)	0.207	120 (28.60%)	107 (35.40%)	0.053	227 (31.50%)
	Nutrition	122 (38.70%)	163 (40.00%)	0.719	141 (36.40%)	144 (43.00%)	0.073	139 (33.10%)	146 (48.30%)	<0.001	285 (39.50%)
	Smoking issue	117 (37.10%)	149 (36.60%)	0.883	148 (38.20%)	118 (35.20%)	0.402	132 (31.40%)	134 (44.40%)	<0.001	266 (36.80%)
	Alcohol use issue	113 (35.90%)	135 (33.20%)	0.448	128 (33.10%)	120 (35.80%)	0.438	125 (29.80%)	123 (40.70%)	0.002	248 (34.30%)
	Physical activity	157 (49.80%)	145 (35.60%)	<0.001	147 (38.00%)	155 (46.30%)	0.024	161 (38.30%)	141 (46.70%)	0.025	302 (41.80%)
	Oral health	69 (22.00%)	90 (22.10%)	0.965	79 (20.40%)	80 (24.00%)	0.253	74 (17.70%)	85 (28.10%)	0.001	159 (22.10%)
From where have you got this information?	School/teachers	299 (94.90%)	397 (97.50%)	0.061	372 (96.10%)	324 (96.70%)	0.670	403 (96.00%)	293 (97.00%)	0.448	696 (96.40%)
	Television/radio	129 (41.00%)	188 (46.20%)	0.159	161 (41.60%)	156 (46.60%)	0.180	173 (41.20%)	144 (47.70%)	0.083	317 (43.90%)
	Family	108 (34.30%)	151 (37.10%)	0.434	160 (41.30%)	99 (29.60%)	0.001	122 (29.00%)	137 (45.40%)	<0.001	259 (35.90%)
	Friends/peers	82 (26.00%)	98 (24.10%)	0.547	97 (25.10%)	83 (24.80%)	0.929	106 (25.20%)	74 (24.50%)	0.822	180 (24.90%)
	Health professionals	98 (31.10%)	115 (28.30%)	0.404	115 (29.70%)	98 (29.30%)	0.892	110 (26.20%)	103 (34.10%)	0.021	213 (29.50%)
	Internet	131 (41.60%)	138 (33.90%)	0.034	126 (32.60%)	143 (42.70%)	0.005	117 (27.90%)	152 (50.30%)	<0.001	269 (37.30%)
	Books	90 (28.60%)	106 (26.00%)	0.449	98 (25.30%)	98 (29.30%)	0.236	104 (24.80%)	92 (30.50%)	0.089	196 (27.10%)
	Health clubs	35 (11.10%)	57 (14.00%)	0.254	59 (15.20%)	33 (9.90%)	0.031	31 (7.40%)	61 (20.20%)	<0.001	92 (12.80%)
	Religious bodies	83 (26.30%)	118 (29.00%)	0.432	100 (25.80%)	101 (30.10%)	0.198	120 (28.60%)	81 (26.80%)	0.605	201 (27.80%)

The respondents were asked whether they had ever engaged in seeking health information, and 73.40% reported ‘Yes’, and the remaining, 26.60%, reported ‘No’. Chi-square test found statistically significant difference based on sex (Male (69.20%) vs. Female (76.70%) responded ‘Yes’ with p-value of 0.025), indicating that females were more likely than males to engage in seeking health information. The respondents reported the reasons for seeking health information as for treatment and/or management of personal health problem (38.80%), prevention and maintenance of personal health (70.20%), to help parents (21.90%), to help siblings (11.30%), to help relatives (8.50%), and to learn about health issues (38.90%). Chi-square test revealed that respondents from private schools were more likely than those from public schools to engage in searching for health information to learn about health issues (p = 0.020).

Specific health topics searched for included mental health (42.90%), sexual and reproductive health (40.60%), and chronic conditions related (such as cancer, blood pressure, diabetes, and asthma) (16.90%). Additionally, there were also those who reported that it was specifically about nutrition, including the benefits of fruits, about the side effects of medications, skin (face) health, weight management, gastric issues, eye care, malaria, kidney, dental care, addiction related, and others. Sources usually consulted for these purposes included parents (60.80%), friends (20.60%), the Internet (17.30%), health professionals (13.60%), and others as shown in Table 3. In addition, there were those who reported obtaining the needed information by reading books, regularly attending health programs on television, and consulting their grandmothers. However, the respondents reported their most trusted sources as health professionals (76.50%), followed by the Internet (22.90%), television/radio (16.80%), and family (15.90%). In several cases, usually consulted sources varied based on school type and grade level. Respondents from private schools were more likely than those from public schools to consult parents (p = 0.039) and search the Internet (p < 0.001). Respondents in grades 11–12 were also more likely than those in grades 9–10 to search the Internet (p < 0.001), while respondents from public schools were more likely to consult friends (p = 0.022).

**Table 3:** Health information seeking, reasons, usually consulted sources, and trusted sources by sex, grade level, and school type.

Questions	Responses	Sex			Grade level			School type			Total n (%)
		Male n (%)	Female n (%)	P-value	9-10 n (%)	11-12 n (%)	P-value	Public n (%)	Private n (%)	P-value	
Interest in learning about health	Yes	219 (69.50%)	330 (81.10%)	0.001	280 (72.40%)	269 (80.30%)	0.020	331 (78.80%)	218 (72.20%)	0.096	549 (76.00%)
	To some extent	70 (22.20%)	60 (14.70%)		84 (21.70%)	46 (13.70%)		65 (15.50%)	65 (21.50%)		130 (18.00%)
	No, currently	26 (8.30%)	17 (4.20%)		23 (5.90%)	20 (6.00%)		24 (5.70%)	19 (6.30%)		43 (6.00%)
Practically seeking health information	Yes	218 (69.20%)	312 (76.70%)	0.025	275 (71.10%)	255 (76.10%)	0.125	306 (72.90%)	224 (74.20%)	0.693	530 (73.40%)
	No	97 (30.80%)	95 (23.30%)		112 (28.90%)	80 (23.90%)		114 (27.10%)	78 (25.80%)		192 (26.60%)
Reasons for seeking the information	Treatment& management (personal)	82 (37.60%)	123 (39.50%)	0.653	105 (38.20%)	100 (39.40%)	0.779	116 (38.00%)	89 (39.70%)	0.692	205 (38.80%)
	Prevention& maintenance (personal)	148 (67.90%)	224 (71.80%)	0.334	196 (71.30%)	176 (69.00%)	0.571	214 (69.90%)	158 (70.50%)	0.881	372 (70.20%)
	To help parents	46 (21.10%)	70 (22.40%)	0.715	58 (21.10%)	58 (22.70%)	0.645	60 (19.60%)	56 (25.00%)	0.138	116 (21.90%)
	To help siblings	26 (11.90%)	34 (10.90%)	0.713	36 (13.10%)	24 (9.40%)	0.182	38 (12.40%)	22 (9.80%)	0.351	60 (11.30%)
	To help relatives	21 (9.60%)	24 (7.70%)	0.430	27 (9.80%)	18 (7.10%)	0.255	25 (8.20%)	20 (8.90%)	0.757	4 (8.50%)
	To learn about health issues	84 (38.50%)	122 (39.10%)	0.895	103 (37.50%)	103 (40.40%)	0.488	106 (34.60%)	100 (44.60%)	0.020	206 (38.90%)
Specific health topics needed	Mental health related	87 (40.30%)	139 (44.70%)	0.314	117 (42.70%)	109 (43.10%)	0.929	135 (44.60%)	91 (40.60%)	0.368	226 (42.90%)
	Sexual and reproductive health related	74 (34.30%)	140 (45.00%)	0.013	114 (41.60%)	100 (39.50%)	0.627	138 (45.50%)	76 (33.90%)	0.007	214 (40.60%)
	Chronic conditions related	33 (15.20%)	56 (18.00%)	0.398	53 (19.30%)	36 (14.20%)	0.113	51 (16.80%)	38 (17.00%)	0.955	89 (16.90%)
Sources usually consulted for these purposes	Parents/guardians	175 (55.60%)	264 (64.90%)	0.011	247 (63.80%)	192 (57.30%)	0.074	242 (57.60%)	197 (65.20%)	0.039	439 (60.80%)
	Siblings	30 (9.50%)	50 (12.30%)	0.241	45 (11.60%)	35 (10.40%)	0.614	54 (12.90%)	26 (8.60%)	0.073	80 (11.10%)
	Friends	70 (22.20%)	79 (19.40%)	0.355	76 (19.60%)	73 (21.80%)	0.476	99 (23.60%)	50 (16.60%)	0.022	149 (20.60%)
	Health professionals	48 (15.20%)	50 (12.30%)	0.251	57 (14.70%)	41 (12.20%)	0.330	56 (13.30%)	42 (13.90%)	0.824	98 (13.60%)
	Teachers	4 (1.30%)	12 (3.00%)	0.127	8 (2.10%)	8 (2.40%)	0.774	10 (2.40%)	6 (2.00%)	0.728	16 (2.20%)
	Internet	60 (19.00%)	65 (16.00%)	0.278	45 (11.60%)	80 (23.90%)	<0.001	36 (8.60%)	89 (29.50%)	<0.001	125 (17.30%)
	Religious figures	11 (3.50%)	7 (1.70%)	0.130	8 (2.10%)	10 (3.00%)	0.430	11 (2.60%)	7 (2.30%)	0.798	18 (2.50%)
Trusted sources	Health professionals	230 (73.00%)	322 (79.10%)	0.055	301 (77.80%)	251 (74.90%)	0.368	328 (78.10%)	224 (74.20%)	0.220	552 (76.50%)
	Books	51 (16.20%)	61 (15.00%)	0.658	54 (14.00%)	58 (17.30%)	0.214	57 (13.60%)	55 (18.20%)	0.089	112 (15.50%)
	Internet	86 (27.30%)	79 (19.40%)	0.012	70 (18.10%)	95 (28.40%)	0.001	51 (12.10%)	114 (37.70%)	<0.001	165 (22.90%)
	Television/radio	60 (19.00%)	61 (15.00%)	0.147	66 (17.10%)	55 (16.40%)	0.819	79 (18.80%)	42 (13.90%)	0.082	121 (16.80%)
	Family	50 (15.90%)	65 (16.00%)	0.972	69 (17.80%)	46 (13.70%)	0.133	43 (10.20%)	72 (23.80%)	<0.001	115 (15.90%)
	Friends	8 (2.50%)	11 (2.70%)	0.892	9 (2.30%)	10 (3.00%)	0.581	11 (2.60%)	8 (2.60%)	0.980	19 (2.60%)
	Teachers	28 (8.90%)	50 (12.30%)	0.145	45 (11.60%)	33 (9.90%)	0.443	37 (8.80%)	41 (13.60%)	0.042	78 (10.80%)
	Health clubs	12 (3.80%)	26 (6.40%)	0.124	20 (5.20%)	18 (5.40%)	0.902	22 (5.20%)	16 (5.30%)	0.972	38 (5.30%)
	Religious bodies	35 (11.10%)	57 (14.00%)	0.247	50 (12.90%)	42 (12.50%)	0.878	54 (12.90%)	38 (12.60%)	0.913	92 (12.70%)

### Perceived major health topics or concerns identified as warranting attention

Topics or issues of major health-related concerns reported as warranting attention included risky sexual behaviors (62.30%), substance use issues (55.30%), mental health challenges (49.40%), and social media addiction (46.80%). Additionally, a number of the respondents identified non-communicable diseases-related issues (such as understanding risk factors for chronic conditions in adulthood or how to prevent them), stress or worries related to education and future employment opportunities, and parents’ problems (conflicts between parents), among others, as important health-related concerns among adolescents. Female respondents were more likely than male respondents to consider risky sexual behaviors (p = 0.037) and mental health issues (p = 0.029) as topics of main health concerns among adolescents. In comparison, males were more likely than females to identify substance use issues (p < 0.001) and social media addiction (p = 0.020) as issues of great health-related concerns among adolescents, as shown in Table 4.

**Table 4:** Perceived major health topics or concerns identified as warranting attention by sex, grade level, and school type.

Topics or issues of great concern	Sex			Grade level			School type			Total n (%)
	Male n (%)	Female n (%)	P-value	9-10 n (%)	11-12 n (%)	P-value	Public n (%)	Private n (%)	P-value	
Sexual and reproductive health issues (risky sexual behaviors)	181 (58.00%)	267 (65.60%)	0.037	247 (64.00%)	201 (60.40%)	0.317	262 (62.70%)	186 (61.80%)	0.809	448 (62.30%)
Substance abuse issues	218 (69.40%)	181 (44.50%)	<0.001	205 (53.00%)	194 (58.10%)	0.169	234 (55.80%)	165 (54.60%)	0.747	399 (55.30%)
Mental health issues	140 (44.70%)	215 (53.00%)	0.029	183 (47.40%)	172 (51.70%)	0.257	208 (49.90%)	147 (48.70%)	0.750	355 (49.40%)
Social media addiction	162 (51.80%)	175 (43.00%)	0.020	173 (44.70%)	164 (49.20%)	0.223	183 (43.80%)	154 (51.00%)	0.056	337 (46.80%)

## Discussion and implications

The study examined health issues and needs among adolescents, revealing multifaceted health challenges and substantial unmet health information and service needs. The findings highlight the importance of supportive and targeted educational, health, and policy initiatives, involving parental and community engagement, to address these challenges and improve adolescent health and well-being.

More than one-quarter (about 28.0%) of the respondents in this study reported recent health problems, including mental health-related issues, reproductive health-related problems, and other issues. Females were more likely than males to experience mental health problems, consistent with prior studies, such as Yoon et al. [43] and Kesanto-Jokipolvi et al. [44] that highlight sex-based disparities in mental health among adolescents. This study found that access to health services was limited, with only one-third of the respondents reporting that they could access services when needed. Adolescents from private schools were more likely to have better access, as they generally came from families with better socioeconomic status, which provide them with greater access to health facilities and services [16,45]. These findings underscore the need to improve access to health services, addressing affordability and confidentiality-related barriers, especially for public school adolescents.

Adolescents in the current study reported exposure to various health topics, most commonly hygiene and sanitation, followed by sexual and reproductive health, the health benefits of physical activity, mental health, and others, but with variations especially based on school type. Private school adolescents were more likely to receive information on diverse health topics, including nutrition, substances use issues such as cigarette and alcohol, and oral health. This likely reflects inequality not only based on school types but also across parental socioeconomic backgrounds. Schools emerged as the primary source of such information, while television/radio, the Internet, family, health professionals, religious bodies, friends/peers, and others, also played important roles, with variations observed by school types. Numerous other studies also reported that various sources play crucial roles in providing adolescents with essential health information [28,29,46,47]. However, compared to other countries such as Greece, where family plays a dominant role [47], the greater reliance on schools observed in the current study suggests cultural differences in parent-child communication about health issues. Introducing and strengthening community-based health education and equipping parents with accurate information and communication skills could help bridge these gaps, while reinforcing schools as key channels for comprehensive health education [16,28,46].

About three-quarters (73.40%) of the participants in the current study reported engaging in seeking health information for personal use (ranging from treatment to health enhancement), as well as to help others. Similarly, Asari et al. [16] reported that adolescents seek health information for their own health concerns and to assist others, especially their parents. In line with the current findings, various studies reported that adolescents commonly search for topics such as mental health, sexual and reproductive health, nutrition, body shape and weight management, and others [46,48]. The current study revealed that females were more likely than males to engage in information-seeking, consistent with prior studies, for instance [46]. This sex-based difference may be explained by the fact that female adolescents are generally more concerned about health [16,49] and may experience more health complaints [49].

Adolescents in the current study consulted multiple sources, including parents, friends, Internet, health professionals, and other sources, to obtain the health information they sought. Although health professionals were considered the most credible source of information (followed by the Internet and television/radio), access was limited, particularly for public school adolescents [16].This problem may not only stem from issues of availability, but also from the lack of adolescent-friendly services, including health information. Variations in usually consulted sources observed based on school type and grade level. These variations may depend on the nature or sensitivity of the health issues [46,50]. For sensitive issues, such as sexual and reproductive health matters, they may often rely on online sources [16,46,51] to maintain confidentiality and privacy [47,52], or on peers/friends, though these sometimes may expose them to misleading information [16,28,29,46,53]. For general health matters, they may consult parents, while open family discussions remain important for all health issues [16]. Socio-economic status also may influence it, affecting access to credible sources, appropriate services, and family communication [16,54]. Likewise, learning style may further shape adolescents’ preferences for health information sources: some prefer audio-visual contents, others value hearing directly from experts (in person or via media), while others prefer reading written materials [16].

Therefore, employing a multi-channel communication approach may be the most effective way to reach adolescents, with reliable, understandable, and engaging health information [28,55,56]. These may include school-based health programs, community-based health education, health education through television, radio, and religious institutions, and locally developed adolescent-friendly websites in native languages.

Adolescents in the current study also identified several issues as important health concerns among their age group. Major areas of concern requiring great attention include risky sexual behaviors, substance use, mental health challenges, and social media addiction, with sex-based differences in perceived priorities.

Overall, the findings reveal complex health challenges, alongside unmet needs in health information and services among adolescents. Addressing these issues requires multifaceted intervention strategies, including comprehensive health education, promotion of health literacy, increased parental and community engagement, accessible adolescent-friendly health services, and supportive policies [6,57–59].

### Strengths and limitations

The study addresses a significant health topic and makes a valuable contribution to the limited literature on the issue, particularly in Ethiopia. It provides crucial insights on adolescent health issues and needs, informing public health policy makers and interventions aiming at improving adolescent wellbeing and strengthening the health of future adult population and society at large. However, to draw conclusions from this study, the following points need to be noted. As the survey was based on self- reports and perceptions, responses may have been prone to response bias. However, as the survey was self-administered and confidential, participants were more likely to respond to the questions honestly and accurately. In addition, since other than adolescents currently attending school were not included in this study, the findings may not represent dropouts and those who were not in school in general. But the proportion of this out of school adolescents might be small, as adolescents are generally in school. Moreover, as the study was cross-sectional, causal inference cannot be made. Besides, although variables such as sex, grade level, and school type were considered as potential factors associated with adolescents’ health issues and needs, other variables were not considered in this study. Future studies should address these gaps. This study nevertheless provides crucial insights and basis for such investigations.

## Conclusion

The study identified multifaceted health concerns and unmet health information and service needs among adolescents. It emphasizes the urgent need for tailored interventions responsive to the needs of diverse sociodemographic groups. These interventions should include effective health communication across multiple channels, health literacy promotion, parental and community engagement, and accessible adolescent-friendly health services to improve the health and well-being of adolescents specifically and society at large.

## Supporting information

S1 File Survey questions and associated statistical results. (DOCX)
