# Supplementary material for: Health issues and needs among adolescents in Ethiopia: A cross-sectional study
Source: PLOS Glob Public Health. 2026 Jun 1;6(6):e0006496. doi: 10.1371/journal.pgph.0006496 (PMC13225383; doi:10.1371/journal.pgph.0006496)
Supplement: S1 File — (DOCX) [file pgph.0006496.s001.docx]

**Supporting Information**

**S1 File:** Survey Questions and Associated Statistical Results

**1. Self-perceived health status:** How do you perceive your overall health status?

How do you perceive your overall health status?  *** Sex**

| **Crosstab** | | | | | |
| --- | --- | --- | --- | --- | --- |
|  | | | Sex | | Total |
|  |  |  | Male | Female |  |
| How do you perceive your health status? | Excellent | Count | 139 | 180 | 319 |
|  |  | % within Sex | 44.1% | 44.2% | 44.2% |
|  |  | % of Total | 19.3% | 24.9% | 44.2% |
|  | Very good | Count | 107 | 124 | 231 |
|  |  | % within Sex | 34.0% | 30.5% | 32.0% |
|  |  | % of Total | 14.8% | 17.2% | 32.0% |
|  | Good | Count | 36 | 60 | 96 |
|  |  | % within Sex | 11.4% | 14.7% | 13.3% |
|  |  | % of Total | 5.0% | 8.3% | 13.3% |
|  | Fair | Count | 24 | 30 | 54 |
|  |  | % within Sex | 7.6% | 7.4% | 7.5% |
|  |  | % of Total | 3.3% | 4.2% | 7.5% |
|  | Poor | Count | 7 | 9 | 16 |
|  |  | % within Sex | 2.2% | 2.2% | 2.2% |
|  |  | % of Total | 1.0% | 1.2% | 2.2% |
|  | Very poor | Count | 2 | 4 | 6 |
|  |  | % within Sex | 0.6% | 1.0% | 0.8% |
|  |  | % of Total | 0.3% | 0.6% | 0.8% |
| Total | | Count | 315 | 407 | 722 |
|  |  | % within Sex | 100.0% | 100.0% | 100.0% |
|  |  | % of Total | 43.6% | 56.4% | 100.0% |

| **Chi-Square Tests** | | | | | | |
| --- | --- | --- | --- | --- | --- | --- |
|  | Value | df | Asymptotic Significance (2-sided) | Exact Sig. (2-sided) | Exact Sig. (1-sided) | Point Probability |
| Pearson Chi-Square | 2.420^a^ | 5 | .788 | .797 |  |  |
| Likelihood Ratio | 2.444 | 5 | .785 | .798 |  |  |
| Fisher-Freeman-Halton Exact Test | 2.456 |  |  | .795 |  |  |
| Linear-by-Linear Association | .245^b^ | 1 | .621 | .633 | .323 | .024 |
| N of Valid Cases | 722 |  |  |  |  |  |
| a. 2 cells (16.7%) have expected count less than 5. The minimum expected count is 2.62. | | | | | | |
| b. The standardized statistic is .495. | | | | | | |

How do you perceive your overall health status?  *** Grade category**

| **Crosstab** | | | | | |
| --- | --- | --- | --- | --- | --- |
|  | | | Grade category | | Total |
|  |  |  | 9-10 | 11-12 |  |
| How do you perceive your health status? | Excellent | Count | 177 | 142 | 319 |
|  |  | % within Grade category | 45.7% | 42.4% | 44.2% |
|  |  | % of Total | 24.5% | 19.7% | 44.2% |
|  | Very good | Count | 120 | 111 | 231 |
|  |  | % within Grade category | 31.0% | 33.1% | 32.0% |
|  |  | % of Total | 16.6% | 15.4% | 32.0% |
|  | Good | Count | 52 | 44 | 96 |
|  |  | % within Grade category | 13.4% | 13.1% | 13.3% |
|  |  | % of Total | 7.2% | 6.1% | 13.3% |
|  | Fair | Count | 23 | 31 | 54 |
|  |  | % within Grade category | 5.9% | 9.3% | 7.5% |
|  |  | % of Total | 3.2% | 4.3% | 7.5% |
|  | Poor | Count | 11 | 5 | 16 |
|  |  | % within Grade category | 2.8% | 1.5% | 2.2% |
|  |  | % of Total | 1.5% | 0.7% | 2.2% |
|  | Very poor | Count | 4 | 2 | 6 |
|  |  | % within Grade category | 1.0% | 0.6% | 0.8% |
|  |  | % of Total | 0.6% | 0.3% | 0.8% |
| Total | | Count | 387 | 335 | 722 |
|  |  | % within Grade category | 100.0% | 100.0% | 100.0% |
|  |  | % of Total | 53.6% | 46.4% | 100.0% |

| **Chi-Square Tests** | | | | | | |
| --- | --- | --- | --- | --- | --- | --- |
|  | Value | df | Asymptotic Significance (2-sided) | Exact Sig. (2-sided) | Exact Sig. (1-sided) | Point Probability |
| Pearson Chi-Square | 5.241^a^ | 5 | .387 | .392 |  |  |
| Likelihood Ratio | 5.293 | 5 | .381 | .398 |  |  |
| Fisher-Freeman-Halton Exact Test | 5.164 |  |  | .395 |  |  |
| Linear-by-Linear Association | .223^b^ | 1 | .636 | .659 | .330 | .024 |
| N of Valid Cases | 722 |  |  |  |  |  |
| a. 2 cells (16.7%) have expected count less than 5. The minimum expected count is 2.78. | | | | | | |
| b. The standardized statistic is .473. | | | | | | |

How do you perceive your overall health status?  *** School type**

| **Crosstab** | | | | | |
| --- | --- | --- | --- | --- | --- |
|  | | | School type | | Total |
|  |  |  | Public | Private |  |
| How do you perceive your health status? | Excellent | Count | 177 | 142 | 319 |
|  |  | % within School type | 42.1% | 47.0% | 44.2% |
|  |  | % of Total | 24.5% | 19.7% | 44.2% |
|  | Very good | Count | 144 | 87 | 231 |
|  |  | % within School type | 34.3% | 28.8% | 32.0% |
|  |  | % of Total | 19.9% | 12.0% | 32.0% |
|  | Good | Count | 62 | 34 | 96 |
|  |  | % within School type | 14.8% | 11.3% | 13.3% |
|  |  | % of Total | 8.6% | 4.7% | 13.3% |
|  | Fair | Count | 27 | 27 | 54 |
|  |  | % within School type | 6.4% | 8.9% | 7.5% |
|  |  | % of Total | 3.7% | 3.7% | 7.5% |
|  | Poor | Count | 6 | 10 | 16 |
|  |  | % within School type | 1.4% | 3.3% | 2.2% |
|  |  | % of Total | 0.8% | 1.4% | 2.2% |
|  | Very poor | Count | 4 | 2 | 6 |
|  |  | % within School type | 1.0% | 0.7% | 0.8% |
|  |  | % of Total | 0.6% | 0.3% | 0.8% |
| Total | | Count | 420 | 302 | 722 |
|  |  | % within School type | 100.0% | 100.0% | 100.0% |
|  |  | % of Total | 58.2% | 41.8% | 100.0% |

| **Chi-Square Tests** | | | | | | |
| --- | --- | --- | --- | --- | --- | --- |
|  | Value | df | Asymptotic Significance (2-sided) | Exact Sig. (2-sided) | Exact Sig. (1-sided) | Point Probability |
| Pearson Chi-Square | 8.685^a^ | 5 | .122 | .120 |  |  |
| Likelihood Ratio | 8.664 | 5 | .123 | .141 |  |  |
| Fisher-Freeman-Halton Exact Test | 8.629 |  |  | .119 |  |  |
| Linear-by-Linear Association | .019^b^ | 1 | .891 | .918 | .458 | .027 |
| N of Valid Cases | 722 |  |  |  |  |  |
| a. 2 cells (16.7%) have expected count less than 5. The minimum expected count is 2.51. | | | | | | |
| b. The standardized statistic is .137. | | | | | | |

**2. Health problem/s experiences:** How often have you experienced or encountered a physical or emotional problem recently, such as within the past month?

How often have you experienced or encountered a physical or emotional problem recently, such as within the past month? *** Sex**

| **Crosstab** | | | | | |
| --- | --- | --- | --- | --- | --- |
|  | | | Sex | | Total |
|  |  |  | Male | Female |  |
| How often have you experienced or encountered health or emotional problem? | Never | Count | 159 | 189 | 348 |
|  |  | % within Sex | 50.5% | 46.4% | 48.2% |
|  |  | % of Total | 22.0% | 26.2% | 48.2% |
|  | Rarely | Count | 85 | 90 | 175 |
|  |  | % within Sex | 27.0% | 22.1% | 24.2% |
|  |  | % of Total | 11.8% | 12.5% | 24.2% |
|  | Sometimes | Count | 59 | 104 | 163 |
|  |  | % within Sex | 18.7% | 25.6% | 22.6% |
|  |  | % of Total | 8.2% | 14.4% | 22.6% |
|  | Often | Count | 11 | 20 | 31 |
|  |  | % within Sex | 3.5% | 4.9% | 4.3% |
|  |  | % of Total | 1.5% | 2.8% | 4.3% |
|  | Everyday | Count | 1 | 4 | 5 |
|  |  | % within Sex | 0.3% | 1.0% | 0.7% |
|  |  | % of Total | 0.1% | 0.6% | 0.7% |
| Total | | Count | 315 | 407 | 722 |
|  |  | % within Sex | 100.0% | 100.0% | 100.0% |
|  |  | % of Total | 43.6% | 56.4% | 100.0% |

| **Chi-Square Tests** | | | | | | |
| --- | --- | --- | --- | --- | --- | --- |
|  | Value | df | Asymptotic Significance (2-sided) | Exact Sig. (2-sided) | Exact Sig. (1-sided) | Point Probability |
| Pearson Chi-Square | 7.972^a^ | 4 | .093 | .089 |  |  |
| Likelihood Ratio | 8.142 | 4 | .087 | .106 |  |  |
| Fisher-Freeman-Halton Exact Test | 7.797 |  |  | .093 |  |  |
| Linear-by-Linear Association | 4.755^b^ | 1 | .029 | .031 | .016 | .003 |
| N of Valid Cases | 722 |  |  |  |  |  |
| a. 2 cells (20.0%) have expected count less than 5. The minimum expected count is 2.18. | | | | | | |
| b. The standardized statistic is 2.181. | | | | | | |

How often have you experienced or encountered a physical or emotional problem recently, such as within the past month?  *** Grade category**

| **Crosstab** | | | | | |
| --- | --- | --- | --- | --- | --- |
|  | | | Grade category | | Total |
|  |  |  | 9-10 | 11-12 |  |
| How often have you experienced or encountered health or emotional problem? | Never | Count | 194 | 154 | 348 |
|  |  | % within Grade category | 50.1% | 46.0% | 48.2% |
|  |  | % of Total | 26.9% | 21.3% | 48.2% |
|  | Rarely | Count | 86 | 89 | 175 |
|  |  | % within Grade category | 22.2% | 26.6% | 24.2% |
|  |  | % of Total | 11.9% | 12.3% | 24.2% |
|  | Sometimes | Count | 89 | 74 | 163 |
|  |  | % within Grade category | 23.0% | 22.1% | 22.6% |
|  |  | % of Total | 12.3% | 10.2% | 22.6% |
|  | Often | Count | 15 | 16 | 31 |
|  |  | % within Grade category | 3.9% | 4.8% | 4.3% |
|  |  | % of Total | 2.1% | 2.2% | 4.3% |
|  | Everyday | Count | 3 | 2 | 5 |
|  |  | % within Grade category | 0.8% | 0.6% | 0.7% |
|  |  | % of Total | 0.4% | 0.3% | 0.7% |
| Total | | Count | 387 | 335 | 722 |
|  |  | % within Grade category | 100.0% | 100.0% | 100.0% |
|  |  | % of Total | 53.6% | 46.4% | 100.0% |

| **Chi-Square Tests** | | | | | | |
| --- | --- | --- | --- | --- | --- | --- |
|  | Value | df | Asymptotic Significance (2-sided) | Exact Sig. (2-sided) | Exact Sig. (1-sided) | Point Probability |
| Pearson Chi-Square | 2.530^a^ | 4 | .639 | .650 |  |  |
| Likelihood Ratio | 2.527 | 4 | .640 | .659 |  |  |
| Fisher-Freeman-Halton Exact Test | 2.617 |  |  | .632 |  |  |
| Linear-by-Linear Association | .398^b^ | 1 | .528 | .534 | .277 | .025 |
| N of Valid Cases | 722 |  |  |  |  |  |
| a. 2 cells (20.0%) have expected count less than 5. The minimum expected count is 2.32. | | | | | | |
| b. The standardized statistic is .631. | | | | | | |

How often have you experienced or encountered a physical or emotional problem recently, such as within the past month? *** School type**

| **Crosstab** | | | | | |
| --- | --- | --- | --- | --- | --- |
|  | | | School type | | Total |
|  |  |  | Public | Private |  |
| How often have you experienced or encountered health or emotional problem? | Never | Count | 210 | 138 | 348 |
|  |  | % within School type | 50.0% | 45.7% | 48.2% |
|  |  | % of Total | 29.1% | 19.1% | 48.2% |
|  | Rarely | Count | 101 | 74 | 175 |
|  |  | % within School type | 24.0% | 24.5% | 24.2% |
|  |  | % of Total | 14.0% | 10.2% | 24.2% |
|  | Sometimes | Count | 92 | 71 | 163 |
|  |  | % within School type | 21.9% | 23.5% | 22.6% |
|  |  | % of Total | 12.7% | 9.8% | 22.6% |
|  | Often | Count | 16 | 15 | 31 |
|  |  | % within School type | 3.8% | 5.0% | 4.3% |
|  |  | % of Total | 2.2% | 2.1% | 4.3% |
|  | Everyday | Count | 1 | 4 | 5 |
|  |  | % within School type | 0.2% | 1.3% | 0.7% |
|  |  | % of Total | 0.1% | 0.6% | 0.7% |
| Total | | Count | 420 | 302 | 722 |
|  |  | % within School type | 100.0% | 100.0% | 100.0% |
|  |  | % of Total | 58.2% | 41.8% | 100.0% |

| **Chi-Square Tests** | | | | | | |
| --- | --- | --- | --- | --- | --- | --- |
|  | Value | df | Asymptotic Significance (2-sided) | Exact Sig. (2-sided) | Exact Sig. (1-sided) | Point Probability |
| Pearson Chi-Square | 4.433^a^ | 4 | .351 | .357 |  |  |
| Likelihood Ratio | 4.488 | 4 | .344 | .367 |  |  |
| Fisher-Freeman-Halton Exact Test | 4.285 |  |  | .370 |  |  |
| Linear-by-Linear Association | 2.516^b^ | 1 | .113 | .116 | .061 | .009 |
| N of Valid Cases | 722 |  |  |  |  |  |
| a. 2 cells (20.0%) have expected count less than 5. The minimum expected count is 2.09. | | | | | | |
| b. The standardized statistic is 1.586. | | | | | | |

**3. Recent health problem/s encountered:** If you did experience a health problem recently, what is it (which problem did you encounter)?

**Mental health related problem (e.g., stress, depression, anxiety) * Sex**

| **Crosstab** | | | | | |
| --- | --- | --- | --- | --- | --- |
|  | | | Sex | | Total |
|  |  |  | Male | Female |  |
| Mental health related problem (e.g., stress, depression, anxiety) | Yes | Count | 66 | 123 | 189 |
|  |  | % within Sex | 42.3% | 56.2% | 50.4% |
|  |  | % of Total | 17.6% | 32.8% | 50.4% |
|  | No | Count | 90 | 96 | 186 |
|  |  | % within Sex | 57.7% | 43.8% | 49.6% |
|  |  | % of Total | 24.0% | 25.6% | 49.6% |
| Total | | Count | 156 | 219 | 375 |
|  |  | % within Sex | 100.0% | 100.0% | 100.0% |
|  |  | % of Total | 41.6% | 58.4% | 100.0% |

| **Chi-Square Tests** | | | | | |
| --- | --- | --- | --- | --- | --- |
|  | Value | df | Asymptotic Significance (2-sided) | Exact Sig. (2-sided) | Exact Sig. (1-sided) |
| Pearson Chi-Square | 6.998^a^ | 1 | .008 |  |  |
| Continuity Correction^b^ | 6.454 | 1 | .011 |  |  |
| Likelihood Ratio | 7.020 | 1 | .008 |  |  |
| Fisher's Exact Test |  |  |  | .009 | .005 |
| Linear-by-Linear Association | 6.979 | 1 | .008 |  |  |
| N of Valid Cases | 375 |  |  |  |  |
| a. 0 cells (0.0%) have expected count less than 5. The minimum expected count is 77.38. | | | | | |
| b. Computed only for a 2x2 table | | | | | |

**Mental health related problem (e.g., stress, depression, anxiety) * Grade category**

| **Crosstab** | | | | | |
| --- | --- | --- | --- | --- | --- |
|  | | | Grade category | | Total |
|  |  |  | 9-10 | 11-12 |  |
| Mental health related problem (e.g., stress, depression, anxiety) | Yes | Count | 94 | 95 | 189 |
|  |  | % within Grade category | 48.7% | 52.2% | 50.4% |
|  |  | % of Total | 25.1% | 25.3% | 50.4% |
|  | No | Count | 99 | 87 | 186 |
|  |  | % within Grade category | 51.3% | 47.8% | 49.6% |
|  |  | % of Total | 26.4% | 23.2% | 49.6% |
| Total | | Count | 193 | 182 | 375 |
|  |  | % within Grade category | 100.0% | 100.0% | 100.0% |
|  |  | % of Total | 51.5% | 48.5% | 100.0% |

| **Chi-Square Tests** | | | | | |
| --- | --- | --- | --- | --- | --- |
|  | Value | df | Asymptotic Significance (2-sided) | Exact Sig. (2-sided) | Exact Sig. (1-sided) |
| Pearson Chi-Square | .457^a^ | 1 | .499 |  |  |
| Continuity Correction^b^ | .328 | 1 | .567 |  |  |
| Likelihood Ratio | .457 | 1 | .499 |  |  |
| Fisher's Exact Test |  |  |  | .536 | .283 |
| Linear-by-Linear Association | .456 | 1 | .500 |  |  |
| N of Valid Cases | 375 |  |  |  |  |
| a. 0 cells (0.0%) have expected count less than 5. The minimum expected count is 90.27. | | | | | |
| b. Computed only for a 2x2 table | | | | | |

**Mental health related problem (e.g., stress, depression, anxiety) * School type**

| **Crosstab** | | | | | |
| --- | --- | --- | --- | --- | --- |
|  | | | School type | | Total |
|  |  |  | Public | Private |  |
| Mental health related problem (e.g., stress, depression, anxiety) | Yes | Count | 98 | 91 | 189 |
|  |  | % within School type | 46.4% | 55.5% | 50.4% |
|  |  | % of Total | 26.1% | 24.3% | 50.4% |
|  | No | Count | 113 | 73 | 186 |
|  |  | % within School type | 53.6% | 44.5% | 49.6% |
|  |  | % of Total | 30.1% | 19.5% | 49.6% |
| Total | | Count | 211 | 164 | 375 |
|  |  | % within School type | 100.0% | 100.0% | 100.0% |
|  |  | % of Total | 56.3% | 43.7% | 100.0% |

| **Chi-Square Tests** | | | | | |
| --- | --- | --- | --- | --- | --- |
|  | Value | df | Asymptotic Significance (2-sided) | Exact Sig. (2-sided) | Exact Sig. (1-sided) |
| Pearson Chi-Square | 3.018^a^ | 1 | .082 |  |  |
| Continuity Correction^b^ | 2.667 | 1 | .102 |  |  |
| Likelihood Ratio | 3.023 | 1 | .082 |  |  |
| Fisher's Exact Test |  |  |  | .096 | .051 |
| Linear-by-Linear Association | 3.010 | 1 | .083 |  |  |
| N of Valid Cases | 375 |  |  |  |  |
| a. 0 cells (0.0%) have expected count less than 5. The minimum expected count is 81.34. | | | | | |
| b. Computed only for a 2x2 table | | | | | |

**Sexual and reproductive health related problem * Sex**

| **Crosstab** | | | | | |
| --- | --- | --- | --- | --- | --- |
|  | | | Sex | | Total |
|  |  |  | Male | Female |  |
| Sexual and reproductive health realted problem | Yes | Count | 15 | 30 | 45 |
|  |  | % within Sex | 9.6% | 13.6% | 11.9% |
|  |  | % of Total | 4.0% | 8.0% | 11.9% |
|  | No | Count | 141 | 191 | 332 |
|  |  | % within Sex | 90.4% | 86.4% | 88.1% |
|  |  | % of Total | 37.4% | 50.7% | 88.1% |
| Total | | Count | 156 | 221 | 377 |
|  |  | % within Sex | 100.0% | 100.0% | 100.0% |
|  |  | % of Total | 41.4% | 58.6% | 100.0% |

| **Chi-Square Tests** | | | | | |
| --- | --- | --- | --- | --- | --- |
|  | Value | df | Asymptotic Significance (2-sided) | Exact Sig. (2-sided) | Exact Sig. (1-sided) |
| Pearson Chi-Square | 1.364^a^ | 1 | .243 |  |  |
| Continuity Correction^b^ | 1.013 | 1 | .314 |  |  |
| Likelihood Ratio | 1.393 | 1 | .238 |  |  |
| Fisher's Exact Test |  |  |  | .263 | .157 |
| Linear-by-Linear Association | 1.360 | 1 | .244 |  |  |
| N of Valid Cases | 377 |  |  |  |  |
| a. 0 cells (0.0%) have expected count less than 5. The minimum expected count is 18.62. | | | | | |
| b. Computed only for a 2x2 table | | | | | |

**Sexual and reproductive health related problem * Grade category**

| **Crosstab** | | | | | |
| --- | --- | --- | --- | --- | --- |
|  | | | Grade category | | Total |
|  |  |  | 9-10 | 11-12 |  |
| Sexual and reproductive health related problem | Yes | Count | 29 | 16 | 45 |
|  |  | % within Grade category | 15.0% | 8.7% | 11.9% |
|  |  | % of Total | 7.7% | 4.2% | 11.9% |
|  | No | Count | 164 | 168 | 332 |
|  |  | % within Grade category | 85.0% | 91.3% | 88.1% |
|  |  | % of Total | 43.5% | 44.6% | 88.1% |
| Total | | Count | 193 | 184 | 377 |
|  |  | % within Grade category | 100.0% | 100.0% | 100.0% |
|  |  | % of Total | 51.2% | 48.8% | 100.0% |

| **Chi-Square Tests** | | | | | |
| --- | --- | --- | --- | --- | --- |
|  | Value | df | Asymptotic Significance (2-sided) | Exact Sig. (2-sided) | Exact Sig. (1-sided) |
| Pearson Chi-Square | 3.591^a^ | 1 | .058 |  |  |
| Continuity Correction^b^ | 3.014 | 1 | .083 |  |  |
| Likelihood Ratio | 3.643 | 1 | .056 |  |  |
| Fisher's Exact Test |  |  |  | .080 | .041 |
| Linear-by-Linear Association | 3.581 | 1 | .058 |  |  |
| N of Valid Cases | 377 |  |  |  |  |
| a. 0 cells (0.0%) have expected count less than 5. The minimum expected count is 21.96. | | | | | |
| b. Computed only for a 2x2 table | | | | | |

**Sexual and reproductive health related problem * School type**

| **Crosstab** | | | | | |
| --- | --- | --- | --- | --- | --- |
|  | | | School type | | Total |
|  |  |  | Public | Private |  |
| Sexual and reproductive health realted problem | Yes | Count | 26 | 19 | 45 |
|  |  | % within School type | 12.2% | 11.6% | 11.9% |
|  |  | % of Total | 6.9% | 5.0% | 11.9% |
|  | No | Count | 187 | 145 | 332 |
|  |  | % within School type | 87.8% | 88.4% | 88.1% |
|  |  | % of Total | 49.6% | 38.5% | 88.1% |
| Total | | Count | 213 | 164 | 377 |
|  |  | % within School type | 100.0% | 100.0% | 100.0% |
|  |  | % of Total | 56.5% | 43.5% | 100.0% |

| **Chi-Square Tests** | | | | | |
| --- | --- | --- | --- | --- | --- |
|  | Value | df | Asymptotic Significance (2-sided) | Exact Sig. (2-sided) | Exact Sig. (1-sided) |
| Pearson Chi-Square | .034^a^ | 1 | .854 |  |  |
| Continuity Correction^b^ | .001 | 1 | .981 |  |  |
| Likelihood Ratio | .034 | 1 | .854 |  |  |
| Fisher's Exact Test |  |  |  | .874 | .492 |
| Linear-by-Linear Association | .034 | 1 | .854 |  |  |
| N of Valid Cases | 377 |  |  |  |  |
| a. 0 cells (0.0%) have expected count less than 5. The minimum expected count is 19.58. | | | | | |
| b. Computed only for a 2x2 table | | | | | |

**Sleep difficulty * Sex**

| **Crosstab** | | | | | |
| --- | --- | --- | --- | --- | --- |
|  | | | Sex | | Total |
|  |  |  | Male | Female |  |
| Sleep difficulty | Yes | Count | 36 | 35 | 71 |
|  |  | % within Sex | 23.1% | 16.0% | 18.9% |
|  |  | % of Total | 9.6% | 9.3% | 18.9% |
|  | No | Count | 120 | 184 | 304 |
|  |  | % within Sex | 76.9% | 84.0% | 81.1% |
|  |  | % of Total | 32.0% | 49.1% | 81.1% |
| Total | | Count | 156 | 219 | 375 |
|  |  | % within Sex | 100.0% | 100.0% | 100.0% |
|  |  | % of Total | 41.6% | 58.4% | 100.0% |

| **Chi-Square Tests** | | | | | |
| --- | --- | --- | --- | --- | --- |
|  | Value | df | Asymptotic Significance (2-sided) | Exact Sig. (2-sided) | Exact Sig. (1-sided) |
| Pearson Chi-Square | 2.988^a^ | 1 | .084 |  |  |
| Continuity Correction^b^ | 2.544 | 1 | .111 |  |  |
| Likelihood Ratio | 2.955 | 1 | .086 |  |  |
| Fisher's Exact Test |  |  |  | .108 | .056 |
| Linear-by-Linear Association | 2.980 | 1 | .084 |  |  |
| N of Valid Cases | 375 |  |  |  |  |
| a. 0 cells (0.0%) have expected count less than 5. The minimum expected count is 29.54. | | | | | |
| b. Computed only for a 2x2 table | | | | | |

**Sleep difficulty * Grade category**

| **Crosstab** | | | | | |
| --- | --- | --- | --- | --- | --- |
|  | | | Grade category | | Total |
|  |  |  | 9-10 | 11-12 |  |
| Sleep difficulty | Yes | Count | 34 | 37 | 71 |
|  |  | % within Grade category | 17.6% | 20.3% | 18.9% |
|  |  | % of Total | 9.1% | 9.9% | 18.9% |
|  | No | Count | 159 | 145 | 304 |
|  |  | % within Grade category | 82.4% | 79.7% | 81.1% |
|  |  | % of Total | 42.4% | 38.7% | 81.1% |
| Total | | Count | 193 | 182 | 375 |
|  |  | % within Grade category | 100.0% | 100.0% | 100.0% |
|  |  | % of Total | 51.5% | 48.5% | 100.0% |

| **Chi-Square Tests** | | | | | |
| --- | --- | --- | --- | --- | --- |
|  | Value | df | Asymptotic Significance (2-sided) | Exact Sig. (2-sided) | Exact Sig. (1-sided) |
| Pearson Chi-Square | .449^a^ | 1 | .503 |  |  |
| Continuity Correction^b^ | .290 | 1 | .590 |  |  |
| Likelihood Ratio | .449 | 1 | .503 |  |  |
| Fisher's Exact Test |  |  |  | .513 | .295 |
| Linear-by-Linear Association | .448 | 1 | .503 |  |  |
| N of Valid Cases | 375 |  |  |  |  |
| a. 0 cells (0.0%) have expected count less than 5. The minimum expected count is 34.46. | | | | | |
| b. Computed only for a 2x2 table | | | | | |

**Sleep difficulty * School type**

| **Crosstab** | | | | | |
| --- | --- | --- | --- | --- | --- |
|  | | | School type | | Total |
|  |  |  | Public | Private |  |
| Sleep difficulty | Yes | Count | 47 | 24 | 71 |
|  |  | % within School type | 22.3% | 14.6% | 18.9% |
|  |  | % of Total | 12.5% | 6.4% | 18.9% |
|  | No | Count | 164 | 140 | 304 |
|  |  | % within School type | 77.7% | 85.4% | 81.1% |
|  |  | % of Total | 43.7% | 37.3% | 81.1% |
| Total | | Count | 211 | 164 | 375 |
|  |  | % within School type | 100.0% | 100.0% | 100.0% |
|  |  | % of Total | 56.3% | 43.7% | 100.0% |

| **Chi-Square Tests** | | | | | |
| --- | --- | --- | --- | --- | --- |
|  | Value | df | Asymptotic Significance (2-sided) | Exact Sig. (2-sided) | Exact Sig. (1-sided) |
| Pearson Chi-Square | 3.510^a^ | 1 | .061 |  |  |
| Continuity Correction^b^ | 3.030 | 1 | .082 |  |  |
| Likelihood Ratio | 3.577 | 1 | .059 |  |  |
| Fisher's Exact Test |  |  |  | .064 | .040 |
| Linear-by-Linear Association | 3.501 | 1 | .061 |  |  |
| N of Valid Cases | 375 |  |  |  |  |
| a. 0 cells (0.0%) have expected count less than 5. The minimum expected count is 31.05. | | | | | |
| b. Computed only for a 2x2 table | | | | | |

**4. Treatment/s used:** If you did experience a health problem recently, what did you do to manage it or recover?

**Went to health facility (Hospital, clinic, health center) * Sex**

| **Crosstab** | | | | | |
| --- | --- | --- | --- | --- | --- |
|  | | | Sex | | Total |
|  |  |  | Male | Female |  |
| Went to health facility (Hospital, clinic, health center) | Yes | Count | 28 | 55 | 83 |
|  |  | % within Sex | 17.9% | 25.2% | 22.2% |
|  |  | % of Total | 7.5% | 14.7% | 22.2% |
|  | No | Count | 128 | 163 | 291 |
|  |  | % within Sex | 82.1% | 74.8% | 77.8% |
|  |  | % of Total | 34.2% | 43.6% | 77.8% |
| Total | | Count | 156 | 218 | 374 |
|  |  | % within Sex | 100.0% | 100.0% | 100.0% |
|  |  | % of Total | 41.7% | 58.3% | 100.0% |

| **Chi-Square Tests** | | | | | |
| --- | --- | --- | --- | --- | --- |
|  | Value | df | Asymptotic Significance (2-sided) | Exact Sig. (2-sided) | Exact Sig. (1-sided) |
| Pearson Chi-Square | 2.791^a^ | 1 | .095 |  |  |
| Continuity Correction^b^ | 2.386 | 1 | .122 |  |  |
| Likelihood Ratio | 2.839 | 1 | .092 |  |  |
| Fisher's Exact Test |  |  |  | .102 | .060 |
| Linear-by-Linear Association | 2.784 | 1 | .095 |  |  |
| N of Valid Cases | 374 |  |  |  |  |
| a. 0 cells (0.0%) have expected count less than 5. The minimum expected count is 34.62. | | | | | |
| b. Computed only for a 2x2 table | | | | | |

**Went to health facility (Hospital, clinic, health center) * Grade category**

| **Crosstab** | | | | | |
| --- | --- | --- | --- | --- | --- |
|  | | | Grade category | | Total |
|  |  |  | 9-10 | 11-12 |  |
| Went to health facility (Hospital, clinic, health center) | Yes | Count | 44 | 39 | 83 |
|  |  | % within Grade category | 22.8% | 21.5% | 22.2% |
|  |  | % of Total | 11.8% | 10.4% | 22.2% |
|  | No | Count | 149 | 142 | 291 |
|  |  | % within Grade category | 77.2% | 78.5% | 77.8% |
|  |  | % of Total | 39.8% | 38.0% | 77.8% |
| Total | | Count | 193 | 181 | 374 |
|  |  | % within Grade category | 100.0% | 100.0% | 100.0% |
|  |  | % of Total | 51.6% | 48.4% | 100.0% |

| **Chi-Square Tests** | | | | | |
| --- | --- | --- | --- | --- | --- |
|  | Value | df | Asymptotic Significance (2-sided) | Exact Sig. (2-sided) | Exact Sig. (1-sided) |
| Pearson Chi-Square | .085^a^ | 1 | .771 |  |  |
| Continuity Correction^b^ | .028 | 1 | .868 |  |  |
| Likelihood Ratio | .085 | 1 | .771 |  |  |
| Fisher's Exact Test |  |  |  | .804 | .434 |
| Linear-by-Linear Association | .084 | 1 | .771 |  |  |
| N of Valid Cases | 374 |  |  |  |  |
| a. 0 cells (0.0%) have expected count less than 5. The minimum expected count is 40.17. | | | | | |
| b. Computed only for a 2x2 table | | | | | |

**Went to health facility (Hospital, clinic, health center) * School type**

| **Crosstab** | | | | | |
| --- | --- | --- | --- | --- | --- |
|  | | | School type | | Total |
|  |  |  | Public | Private |  |
| Went to health facility (Hospital, clinic, health center) | Yes | Count | 42 | 41 | 83 |
|  |  | % within School type | 20.0% | 25.0% | 22.2% |
|  |  | % of Total | 11.2% | 11.0% | 22.2% |
|  | No | Count | 168 | 123 | 291 |
|  |  | % within School type | 80.0% | 75.0% | 77.8% |
|  |  | % of Total | 44.9% | 32.9% | 77.8% |
| Total | | Count | 210 | 164 | 374 |
|  |  | % within School type | 100.0% | 100.0% | 100.0% |
|  |  | % of Total | 56.1% | 43.9% | 100.0% |

| **Chi-Square Tests** | | | | | |
| --- | --- | --- | --- | --- | --- |
|  | Value | df | Asymptotic Significance (2-sided) | Exact Sig. (2-sided) | Exact Sig. (1-sided) |
| Pearson Chi-Square | 1.333^a^ | 1 | .248 |  |  |
| Continuity Correction^b^ | 1.059 | 1 | .303 |  |  |
| Likelihood Ratio | 1.327 | 1 | .249 |  |  |
| Fisher's Exact Test |  |  |  | .261 | .152 |
| Linear-by-Linear Association | 1.330 | 1 | .249 |  |  |
| N of Valid Cases | 374 |  |  |  |  |
| a. 0 cells (0.0%) have expected count less than 5. The minimum expected count is 36.40. | | | | | |
| b. Computed only for a 2x2 table | | | | | |

**Bought medications from pharmacy * Sex**

| **Crosstab** | | | | | |
| --- | --- | --- | --- | --- | --- |
|  | | | Sex | | Total |
|  |  |  | Male | Female |  |
| Bought medications from pharmacy | Yes | Count | 13 | 16 | 29 |
|  |  | % within Sex | 8.3% | 7.3% | 7.8% |
|  |  | % of Total | 3.5% | 4.3% | 7.8% |
|  | No | Count | 143 | 202 | 345 |
|  |  | % within Sex | 91.7% | 92.7% | 92.2% |
|  |  | % of Total | 38.2% | 54.0% | 92.2% |
| Total | | Count | 156 | 218 | 374 |
|  |  | % within Sex | 100.0% | 100.0% | 100.0% |
|  |  | % of Total | 41.7% | 58.3% | 100.0% |

| **Chi-Square Tests** | | | | | |
| --- | --- | --- | --- | --- | --- |
|  | Value | df | Asymptotic Significance (2-sided) | Exact Sig. (2-sided) | Exact Sig. (1-sided) |
| Pearson Chi-Square | .126^a^ | 1 | .723 |  |  |
| Continuity Correction^b^ | .025 | 1 | .874 |  |  |
| Likelihood Ratio | .125 | 1 | .724 |  |  |
| Fisher's Exact Test |  |  |  | .845 | .434 |
| Linear-by-Linear Association | .125 | 1 | .723 |  |  |
| N of Valid Cases | 374 |  |  |  |  |
| a. 0 cells (0.0%) have expected count less than 5. The minimum expected count is 12.10. | | | | | |
| b. Computed only for a 2x2 table | | | | | |

**Bought medications from pharmacy * Grade category**

| **Crosstab** | | | | | |
| --- | --- | --- | --- | --- | --- |
|  | | | Grade category | | Total |
|  |  |  | 9-10 | 11-12 |  |
| Bought medications from pharmacy | Yes | Count | 12 | 17 | 29 |
|  |  | % within Grade category | 6.2% | 9.4% | 7.8% |
|  |  | % of Total | 3.2% | 4.5% | 7.8% |
|  | No | Count | 181 | 164 | 345 |
|  |  | % within Grade category | 93.8% | 90.6% | 92.2% |
|  |  | % of Total | 48.4% | 43.9% | 92.2% |
| Total | | Count | 193 | 181 | 374 |
|  |  | % within Grade category | 100.0% | 100.0% | 100.0% |
|  |  | % of Total | 51.6% | 48.4% | 100.0% |

| **Chi-Square Tests** | | | | | |
| --- | --- | --- | --- | --- | --- |
|  | Value | df | Asymptotic Significance (2-sided) | Exact Sig. (2-sided) | Exact Sig. (1-sided) |
| Pearson Chi-Square | 1.316^a^ | 1 | .251 |  |  |
| Continuity Correction^b^ | .910 | 1 | .340 |  |  |
| Likelihood Ratio | 1.319 | 1 | .251 |  |  |
| Fisher's Exact Test |  |  |  | .334 | .170 |
| Linear-by-Linear Association | 1.313 | 1 | .252 |  |  |
| N of Valid Cases | 374 |  |  |  |  |
| a. 0 cells (0.0%) have expected count less than 5. The minimum expected count is 14.03. | | | | | |
| b. Computed only for a 2x2 table | | | | | |

**Bought medications from pharmacy * School type**

| **Crosstab** | | | | | |
| --- | --- | --- | --- | --- | --- |
|  | | | School type | | Total |
|  |  |  | Public | Private |  |
| Bought medications from pharmacy | Yes | Count | 18 | 11 | 29 |
|  |  | % within School type | 8.6% | 6.7% | 7.8% |
|  |  | % of Total | 4.8% | 2.9% | 7.8% |
|  | No | Count | 192 | 153 | 345 |
|  |  | % within School type | 91.4% | 93.3% | 92.2% |
|  |  | % of Total | 51.3% | 40.9% | 92.2% |
| Total | | Count | 210 | 164 | 374 |
|  |  | % within School type | 100.0% | 100.0% | 100.0% |
|  |  | % of Total | 56.1% | 43.9% | 100.0% |

| **Chi-Square Tests** | | | | | |
| --- | --- | --- | --- | --- | --- |
|  | Value | df | Asymptotic Significance (2-sided) | Exact Sig. (2-sided) | Exact Sig. (1-sided) |
| Pearson Chi-Square | .447^a^ | 1 | .504 |  |  |
| Continuity Correction^b^ | .225 | 1 | .635 |  |  |
| Likelihood Ratio | .452 | 1 | .501 |  |  |
| Fisher's Exact Test |  |  |  | .563 | .320 |
| Linear-by-Linear Association | .446 | 1 | .504 |  |  |
| N of Valid Cases | 374 |  |  |  |  |
| a. 0 cells (0.0%) have expected count less than 5. The minimum expected count is 12.72. | | | | | |
| b. Computed only for a 2x2 table | | | | | |

**Used traditional medications * Sex**

| **Crosstab** | | | | | |
| --- | --- | --- | --- | --- | --- |
|  | | | Sex | | Total |
|  |  |  | Male | Female |  |
| Used traditional medications | Yes | Count | 9 | 14 | 23 |
|  |  | % within Sex | 5.8% | 6.4% | 6.1% |
|  |  | % of Total | 2.4% | 3.7% | 6.1% |
|  | No | Count | 147 | 204 | 351 |
|  |  | % within Sex | 94.2% | 93.6% | 93.9% |
|  |  | % of Total | 39.3% | 54.5% | 93.9% |
| Total | | Count | 156 | 218 | 374 |
|  |  | % within Sex | 100.0% | 100.0% | 100.0% |
|  |  | % of Total | 41.7% | 58.3% | 100.0% |

| **Chi-Square Tests** | | | | | |
| --- | --- | --- | --- | --- | --- |
|  | Value | df | Asymptotic Significance (2-sided) | Exact Sig. (2-sided) | Exact Sig. (1-sided) |
| Pearson Chi-Square | .067^a^ | 1 | .796 |  |  |
| Continuity Correction^b^ | .002 | 1 | .967 |  |  |
| Likelihood Ratio | .068 | 1 | .795 |  |  |
| Fisher's Exact Test |  |  |  | .831 | .488 |
| Linear-by-Linear Association | .067 | 1 | .796 |  |  |
| N of Valid Cases | 374 |  |  |  |  |
| a. 0 cells (0.0%) have expected count less than 5. The minimum expected count is 9.59. | | | | | |
| b. Computed only for a 2x2 table | | | | | |

**Used traditional medications * Grade category**

| **Crosstab** | | | | | |
| --- | --- | --- | --- | --- | --- |
|  | | | Grade category | | Total |
|  |  |  | 9-10 | 11-12 |  |
| Used traditional medications | Yes | Count | 14 | 9 | 23 |
|  |  | % within Grade category | 7.3% | 5.0% | 6.1% |
|  |  | % of Total | 3.7% | 2.4% | 6.1% |
|  | No | Count | 179 | 172 | 351 |
|  |  | % within Grade category | 92.7% | 95.0% | 93.9% |
|  |  | % of Total | 47.9% | 46.0% | 93.9% |
| Total | | Count | 193 | 181 | 374 |
|  |  | % within Grade category | 100.0% | 100.0% | 100.0% |
|  |  | % of Total | 51.6% | 48.4% | 100.0% |

| **Chi-Square Tests** | | | | | |
| --- | --- | --- | --- | --- | --- |
|  | Value | df | Asymptotic Significance (2-sided) | Exact Sig. (2-sided) | Exact Sig. (1-sided) |
| Pearson Chi-Square | .842^a^ | 1 | .359 |  |  |
| Continuity Correction^b^ | .493 | 1 | .482 |  |  |
| Likelihood Ratio | .850 | 1 | .356 |  |  |
| Fisher's Exact Test |  |  |  | .396 | .242 |
| Linear-by-Linear Association | .840 | 1 | .359 |  |  |
| N of Valid Cases | 374 |  |  |  |  |
| a. 0 cells (0.0%) have expected count less than 5. The minimum expected count is 11.13. | | | | | |
| b. Computed only for a 2x2 table | | | | | |

**Used traditional medications * School type**

| **Crosstab** | | | | | |
| --- | --- | --- | --- | --- | --- |
|  | | | School type | | Total |
|  |  |  | Public | Private |  |
| Used traditional medications | Yes | Count | 16 | 7 | 23 |
|  |  | % within School type | 7.6% | 4.3% | 6.1% |
|  |  | % of Total | 4.3% | 1.9% | 6.1% |
|  | No | Count | 194 | 157 | 351 |
|  |  | % within School type | 92.4% | 95.7% | 93.9% |
|  |  | % of Total | 51.9% | 42.0% | 93.9% |
| Total | | Count | 210 | 164 | 374 |
|  |  | % within School type | 100.0% | 100.0% | 100.0% |
|  |  | % of Total | 56.1% | 43.9% | 100.0% |

| **Chi-Square Tests** | | | | | |
| --- | --- | --- | --- | --- | --- |
|  | Value | df | Asymptotic Significance (2-sided) | Exact Sig. (2-sided) | Exact Sig. (1-sided) |
| Pearson Chi-Square | 1.791^a^ | 1 | .181 |  |  |
| Continuity Correction^b^ | 1.258 | 1 | .262 |  |  |
| Likelihood Ratio | 1.853 | 1 | .173 |  |  |
| Fisher's Exact Test |  |  |  | .200 | .130 |
| Linear-by-Linear Association | 1.787 | 1 | .181 |  |  |
| N of Valid Cases | 374 |  |  |  |  |
| a. 0 cells (0.0%) have expected count less than 5. The minimum expected count is 10.09. | | | | | |
| b. Computed only for a 2x2 table | | | | | |

**Used religious healing practices/prayer * Sex**

| **Crosstab** | | | | | |
| --- | --- | --- | --- | --- | --- |
|  | | | Sex | | Total |
|  |  |  | Male | Female |  |
| Used religious healing practices/prayer | Yes | Count | 33 | 65 | 98 |
|  |  | % within Sex | 21.2% | 29.8% | 26.2% |
|  |  | % of Total | 8.8% | 17.4% | 26.2% |
|  | No | Count | 123 | 153 | 276 |
|  |  | % within Sex | 78.8% | 70.2% | 73.8% |
|  |  | % of Total | 32.9% | 40.9% | 73.8% |
| Total | | Count | 156 | 218 | 374 |
|  |  | % within Sex | 100.0% | 100.0% | 100.0% |
|  |  | % of Total | 41.7% | 58.3% | 100.0% |

| **Chi-Square Tests** | | | | | |
| --- | --- | --- | --- | --- | --- |
|  | Value | df | Asymptotic Significance (2-sided) | Exact Sig. (2-sided) | Exact Sig. (1-sided) |
| Pearson Chi-Square | 3.529^a^ | 1 | .060 |  |  |
| Continuity Correction^b^ | 3.095 | 1 | .079 |  |  |
| Likelihood Ratio | 3.585 | 1 | .058 |  |  |
| Fisher's Exact Test |  |  |  | .073 | .039 |
| Linear-by-Linear Association | 3.519 | 1 | .061 |  |  |
| N of Valid Cases | 374 |  |  |  |  |
| a. 0 cells (0.0%) have expected count less than 5. The minimum expected count is 40.88. | | | | | |
| b. Computed only for a 2x2 table | | | | | |

**Used religious healing practices/prayer * Grade category**

| **Crosstab** | | | | | |
| --- | --- | --- | --- | --- | --- |
|  | | | Grade category | | Total |
|  |  |  | 9-10 | 11-12 |  |
| Used religious healing practices/prayer | Yes | Count | 57 | 41 | 98 |
|  |  | % within Grade category | 29.5% | 22.7% | 26.2% |
|  |  | % of Total | 15.2% | 11.0% | 26.2% |
|  | No | Count | 136 | 140 | 276 |
|  |  | % within Grade category | 70.5% | 77.3% | 73.8% |
|  |  | % of Total | 36.4% | 37.4% | 73.8% |
| Total | | Count | 193 | 181 | 374 |
|  |  | % within Grade category | 100.0% | 100.0% | 100.0% |
|  |  | % of Total | 51.6% | 48.4% | 100.0% |

| **Chi-Square Tests** | | | | | |
| --- | --- | --- | --- | --- | --- |
|  | Value | df | Asymptotic Significance (2-sided) | Exact Sig. (2-sided) | Exact Sig. (1-sided) |
| Pearson Chi-Square | 2.288^a^ | 1 | .130 |  |  |
| Continuity Correction^b^ | 1.946 | 1 | .163 |  |  |
| Likelihood Ratio | 2.297 | 1 | .130 |  |  |
| Fisher's Exact Test |  |  |  | .158 | .081 |
| Linear-by-Linear Association | 2.281 | 1 | .131 |  |  |
| N of Valid Cases | 374 |  |  |  |  |
| a. 0 cells (0.0%) have expected count less than 5. The minimum expected count is 47.43. | | | | | |
| b. Computed only for a 2x2 table | | | | | |

**Used religious healing practices/prayer * School type**

| **Crosstab** | | | | | |
| --- | --- | --- | --- | --- | --- |
|  | | | School type | | Total |
|  |  |  | Public | Private |  |
| Used religious healing practices/prayer | Yes | Count | 57 | 41 | 98 |
|  |  | % within School type | 27.1% | 25.0% | 26.2% |
|  |  | % of Total | 15.2% | 11.0% | 26.2% |
|  | No | Count | 153 | 123 | 276 |
|  |  | % within School type | 72.9% | 75.0% | 73.8% |
|  |  | % of Total | 40.9% | 32.9% | 73.8% |
| Total | | Count | 210 | 164 | 374 |
|  |  | % within School type | 100.0% | 100.0% | 100.0% |
|  |  | % of Total | 56.1% | 43.9% | 100.0% |

| **Chi-Square Tests** | | | | | |
| --- | --- | --- | --- | --- | --- |
|  | Value | df | Asymptotic Significance (2-sided) | Exact Sig. (2-sided) | Exact Sig. (1-sided) |
| Pearson Chi-Square | .219^a^ | 1 | .640 |  |  |
| Continuity Correction^b^ | .122 | 1 | .727 |  |  |
| Likelihood Ratio | .219 | 1 | .640 |  |  |
| Fisher's Exact Test |  |  |  | .722 | .364 |
| Linear-by-Linear Association | .218 | 1 | .641 |  |  |
| N of Valid Cases | 374 |  |  |  |  |
| a. 0 cells (0.0%) have expected count less than 5. The minimum expected count is 42.97. | | | | | |
| b. Computed only for a 2x2 table | | | | | |

**5. Access to health services:** Could you able to get health services when needed?

**Access to health services * Sex**

| **Crosstab** | | | | | |
| --- | --- | --- | --- | --- | --- |
|  | | | Sex | | Total |
|  |  |  | Male | Female |  |
| Access to health services | Yes, properly | Count | 90 | 146 | 236 |
|  |  | % within Sex | 28.6% | 35.9% | 32.7% |
|  |  | % of Total | 12.5% | 20.2% | 32.7% |
|  | Yes, to some extent | Count | 142 | 158 | 300 |
|  |  | % within Sex | 45.1% | 38.8% | 41.6% |
|  |  | % of Total | 19.7% | 21.9% | 41.6% |
|  | No | Count | 83 | 103 | 186 |
|  |  | % within Sex | 26.3% | 25.3% | 25.8% |
|  |  | % of Total | 11.5% | 14.3% | 25.8% |
| Total | | Count | 315 | 407 | 722 |
|  |  | % within Sex | 100.0% | 100.0% | 100.0% |
|  |  | % of Total | 43.6% | 56.4% | 100.0% |

| **Chi-Square Tests** | | | |
| --- | --- | --- | --- |
|  | Value | df | Asymptotic Significance (2-sided) |
| Pearson Chi-Square | 4.644^a^ | 2 | .098 |
| Likelihood Ratio | 4.669 | 2 | .097 |
| Linear-by-Linear Association | 2.129 | 1 | .145 |
| N of Valid Cases | 722 |  |  |
| a. 0 cells (0.0%) have expected count less than 5. The minimum expected count is 81.15. | | | |

**Access to health services * Grade category**

| **Crosstab** | | | | | |
| --- | --- | --- | --- | --- | --- |
|  | | | Grade category | | Total |
|  |  |  | 9-10 | 11-12 |  |
| Access to health services | Yes, properly | Count | 143 | 93 | 236 |
|  |  | % within Grade category | 37.0% | 27.8% | 32.7% |
|  |  | % of Total | 19.8% | 12.9% | 32.7% |
|  | Yes, to some extent | Count | 152 | 148 | 300 |
|  |  | % within Grade category | 39.3% | 44.2% | 41.6% |
|  |  | % of Total | 21.1% | 20.5% | 41.6% |
|  | No | Count | 92 | 94 | 186 |
|  |  | % within Grade category | 23.8% | 28.1% | 25.8% |
|  |  | % of Total | 12.7% | 13.0% | 25.8% |
| Total | | Count | 387 | 335 | 722 |
|  |  | % within Grade category | 100.0% | 100.0% | 100.0% |
|  |  | % of Total | 53.6% | 46.4% | 100.0% |

| **Chi-Square Tests** | | | |
| --- | --- | --- | --- |
|  | Value | df | Asymptotic Significance (2-sided) |
| Pearson Chi-Square | 6.959^a^ | 2 | .031 |
| Likelihood Ratio | 7.000 | 2 | .030 |
| Linear-by-Linear Association | 5.618 | 1 | .018 |
| N of Valid Cases | 722 |  |  |
| a. 0 cells (0.0%) have expected count less than 5. The minimum expected count is 86.30. | | | |

**Access to health services * School type**

| **Crosstab** | | | | | |
| --- | --- | --- | --- | --- | --- |
|  | | | School type | | Total |
|  |  |  | Public | Private |  |
| Access to health services | Yes, properly | Count | 110 | 126 | 236 |
|  |  | % within School type | 26.2% | 41.7% | 32.7% |
|  |  | % of Total | 15.2% | 17.5% | 32.7% |
|  | Yes, to some extent | Count | 191 | 109 | 300 |
|  |  | % within School type | 45.5% | 36.1% | 41.6% |
|  |  | % of Total | 26.5% | 15.1% | 41.6% |
|  | No | Count | 119 | 67 | 186 |
|  |  | % within School type | 28.3% | 22.2% | 25.8% |
|  |  | % of Total | 16.5% | 9.3% | 25.8% |
| Total | | Count | 420 | 302 | 722 |
|  |  | % within School type | 100.0% | 100.0% | 100.0% |
|  |  | % of Total | 58.2% | 41.8% | 100.0% |

| **Chi-Square Tests** | | | |
| --- | --- | --- | --- |
|  | Value | df | Asymptotic Significance (2-sided) |
| Pearson Chi-Square | 19.265^a^ | 2 | .000 |
| Likelihood Ratio | 19.148 | 2 | .000 |
| Linear-by-Linear Association | 14.224 | 1 | .000 |
| N of Valid Cases | 722 |  |  |
| a. 0 cells (0.0%) have expected count less than 5. The minimum expected count is 77.80. | | | |

**6. Exposure to health information or health topics learned:** Which health information or topic have you got or learned about so far?

**Sexual and reproductive health (Puberty, STIs/ STDs/HIV/AIDS, Pregnancy) * Sex**

| **Crosstab** | | | | | |
| --- | --- | --- | --- | --- | --- |
|  | | | Sex | | Total |
|  |  |  | Male | Female |  |
| Sexual and reproductive health (Puberty, STIs/ STDs/HIV/AIDS, Pregnancy) | Yes | Count | 215 | 284 | 499 |
|  |  | % within Sex | 68.3% | 69.8% | 69.1% |
|  |  | % of Total | 29.8% | 39.3% | 69.1% |
|  | No | Count | 100 | 123 | 223 |
|  |  | % within Sex | 31.7% | 30.2% | 30.9% |
|  |  | % of Total | 13.9% | 17.0% | 30.9% |
| Total | | Count | 315 | 407 | 722 |
|  |  | % within Sex | 100.0% | 100.0% | 100.0% |
|  |  | % of Total | 43.6% | 56.4% | 100.0% |

| **Chi-Square Tests** | | | | | |
| --- | --- | --- | --- | --- | --- |
|  | Value | df | Asymptotic Significance (2-sided) | Exact Sig. (2-sided) | Exact Sig. (1-sided) |
| Pearson Chi-Square | .193^a^ | 1 | .660 |  |  |
| Continuity Correction^b^ | .129 | 1 | .720 |  |  |
| Likelihood Ratio | .193 | 1 | .660 |  |  |
| Fisher's Exact Test |  |  |  | .685 | .360 |
| Linear-by-Linear Association | .193 | 1 | .660 |  |  |
| N of Valid Cases | 722 |  |  |  |  |
| a. 0 cells (0.0%) have expected count less than 5. The minimum expected count is 97.29. | | | | | |
| b. Computed only for a 2x2 table | | | | | |

**Sexual and reproductive health (Puberty, STIs/ STDs/HIV/AIDS, Pregnancy) * Grade category**

| **Crosstab** | | | | | |
| --- | --- | --- | --- | --- | --- |
|  | | | Grade category | | Total |
|  |  |  | 9-10 | 11-12 |  |
| Sexual and reproductive health (Puberty, STIs/ STDs/HIV/AIDS, Pregnancy) | Yes | Count | 272 | 227 | 499 |
|  |  | % within Grade category | 70.3% | 67.8% | 69.1% |
|  |  | % of Total | 37.7% | 31.4% | 69.1% |
|  | No | Count | 115 | 108 | 223 |
|  |  | % within Grade category | 29.7% | 32.2% | 30.9% |
|  |  | % of Total | 15.9% | 15.0% | 30.9% |
| Total | | Count | 387 | 335 | 722 |
|  |  | % within Grade category | 100.0% | 100.0% | 100.0% |
|  |  | % of Total | 53.6% | 46.4% | 100.0% |

| **Chi-Square Tests** | | | | | |
| --- | --- | --- | --- | --- | --- |
|  | Value | df | Asymptotic Significance (2-sided) | Exact Sig. (2-sided) | Exact Sig. (1-sided) |
| Pearson Chi-Square | .535^a^ | 1 | .464 |  |  |
| Continuity Correction^b^ | .424 | 1 | .515 |  |  |
| Likelihood Ratio | .535 | 1 | .465 |  |  |
| Fisher's Exact Test |  |  |  | .469 | .257 |
| Linear-by-Linear Association | .535 | 1 | .465 |  |  |
| N of Valid Cases | 722 |  |  |  |  |
| a. 0 cells (0.0%) have expected count less than 5. The minimum expected count is 103.47. | | | | | |
| b. Computed only for a 2x2 table | | | | | |

**Sexual and reproductive health (Puberty, STIs/ STDs/HIV/AIDS, Pregnancy) * School type**

| **Crosstab** | | | | | |
| --- | --- | --- | --- | --- | --- |
|  | | | School type | | Total |
|  |  |  | Public | Private |  |
| Sexual and reproductive health (Puberty, STIs/ STDs/HIV/AIDS, Pregnancy) | Yes | Count | 285 | 214 | 499 |
|  |  | % within School type | 67.9% | 70.9% | 69.1% |
|  |  | % of Total | 39.5% | 29.6% | 69.1% |
|  | No | Count | 135 | 88 | 223 |
|  |  | % within School type | 32.1% | 29.1% | 30.9% |
|  |  | % of Total | 18.7% | 12.2% | 30.9% |
| Total | | Count | 420 | 302 | 722 |
|  |  | % within School type | 100.0% | 100.0% | 100.0% |
|  |  | % of Total | 58.2% | 41.8% | 100.0% |

| **Chi-Square Tests** | | | | | |
| --- | --- | --- | --- | --- | --- |
|  | Value | df | Asymptotic Significance (2-sided) | Exact Sig. (2-sided) | Exact Sig. (1-sided) |
| Pearson Chi-Square | .743^a^ | 1 | .389 |  |  |
| Continuity Correction^b^ | .609 | 1 | .435 |  |  |
| Likelihood Ratio | .745 | 1 | .388 |  |  |
| Fisher's Exact Test |  |  |  | .415 | .218 |
| Linear-by-Linear Association | .742 | 1 | .389 |  |  |
| N of Valid Cases | 722 |  |  |  |  |
| a. 0 cells (0.0%) have expected count less than 5. The minimum expected count is 93.28. | | | | | |
| b. Computed only for a 2x2 table | | | | | |

**Personal hygiene and environmental sanitation * Sex**

| **Crosstab** | | | | | |
| --- | --- | --- | --- | --- | --- |
|  | | | Sex | | Total |
|  |  |  | Male | Female |  |
| Personal hygiene and environmental sanitation | Yes | Count | 266 | 378 | 644 |
|  |  | % within Sex | 84.4% | 92.9% | 89.2% |
|  |  | % of Total | 36.8% | 52.4% | 89.2% |
|  | No | Count | 49 | 29 | 78 |
|  |  | % within Sex | 15.6% | 7.1% | 10.8% |
|  |  | % of Total | 6.8% | 4.0% | 10.8% |
| Total | | Count | 315 | 407 | 722 |
|  |  | % within Sex | 100.0% | 100.0% | 100.0% |
|  |  | % of Total | 43.6% | 56.4% | 100.0% |

| **Chi-Square Tests** | | | | | |
| --- | --- | --- | --- | --- | --- |
|  | Value | df | Asymptotic Significance (2-sided) | Exact Sig. (2-sided) | Exact Sig. (1-sided) |
| Pearson Chi-Square | 13.096^a^ | 1 | .000 |  |  |
| Continuity Correction^b^ | 12.236 | 1 | .000 |  |  |
| Likelihood Ratio | 13.009 | 1 | .000 |  |  |
| Fisher's Exact Test |  |  |  | .000 | .000 |
| Linear-by-Linear Association | 13.078 | 1 | .000 |  |  |
| N of Valid Cases | 722 |  |  |  |  |
| a. 0 cells (0.0%) have expected count less than 5. The minimum expected count is 34.03. | | | | | |
| b. Computed only for a 2x2 table | | | | | |

**Personal hygiene and environmental sanitation * Grade category**

| **Crosstab** | | | | | |
| --- | --- | --- | --- | --- | --- |
|  | | | Grade category | | Total |
|  |  |  | 9-10 | 11-12 |  |
| Personal hygiene and environmental sanitation | Yes | Count | 338 | 306 | 644 |
|  |  | % within Grade category | 87.3% | 91.3% | 89.2% |
|  |  | % of Total | 46.8% | 42.4% | 89.2% |
|  | No | Count | 49 | 29 | 78 |
|  |  | % within Grade category | 12.7% | 8.7% | 10.8% |
|  |  | % of Total | 6.8% | 4.0% | 10.8% |
| Total | | Count | 387 | 335 | 722 |
|  |  | % within Grade category | 100.0% | 100.0% | 100.0% |
|  |  | % of Total | 53.6% | 46.4% | 100.0% |

| **Chi-Square Tests** | | | | | |
| --- | --- | --- | --- | --- | --- |
|  | Value | df | Asymptotic Significance (2-sided) | Exact Sig. (2-sided) | Exact Sig. (1-sided) |
| Pearson Chi-Square | 2.989^a^ | 1 | .084 |  |  |
| Continuity Correction^b^ | 2.587 | 1 | .108 |  |  |
| Likelihood Ratio | 3.028 | 1 | .082 |  |  |
| Fisher's Exact Test |  |  |  | .093 | .053 |
| Linear-by-Linear Association | 2.984 | 1 | .084 |  |  |
| N of Valid Cases | 722 |  |  |  |  |
| a. 0 cells (0.0%) have expected count less than 5. The minimum expected count is 36.19. | | | | | |
| b. Computed only for a 2x2 table | | | | | |

**Personal hygiene and environmental sanitation * School type**

| **Crosstab** | | | | | |
| --- | --- | --- | --- | --- | --- |
|  | | | School type | | Total |
|  |  |  | Public | Private |  |
| Personal hygiene and environmental sanitation | Yes | Count | 372 | 272 | 644 |
|  |  | % within School type | 88.6% | 90.1% | 89.2% |
|  |  | % of Total | 51.5% | 37.7% | 89.2% |
|  | No | Count | 48 | 30 | 78 |
|  |  | % within School type | 11.4% | 9.9% | 10.8% |
|  |  | % of Total | 6.6% | 4.2% | 10.8% |
| Total | | Count | 420 | 302 | 722 |
|  |  | % within School type | 100.0% | 100.0% | 100.0% |
|  |  | % of Total | 58.2% | 41.8% | 100.0% |

| **Chi-Square Tests** | | | | | |
| --- | --- | --- | --- | --- | --- |
|  | Value | df | Asymptotic Significance (2-sided) | Exact Sig. (2-sided) | Exact Sig. (1-sided) |
| Pearson Chi-Square | .407^a^ | 1 | .523 |  |  |
| Continuity Correction^b^ | .267 | 1 | .605 |  |  |
| Likelihood Ratio | .410 | 1 | .522 |  |  |
| Fisher's Exact Test |  |  |  | .546 | .304 |
| Linear-by-Linear Association | .407 | 1 | .524 |  |  |
| N of Valid Cases | 722 |  |  |  |  |
| a. 0 cells (0.0%) have expected count less than 5. The minimum expected count is 32.63. | | | | | |
| b. Computed only for a 2x2 table | | | | | |

**Mental health * Sex**

| **Crosstab** | | | | | |
| --- | --- | --- | --- | --- | --- |
|  | | | Sex | | Total |
|  |  |  | Male | Female |  |
| Mental health | Yes | Count | 97 | 130 | 227 |
|  |  | % within Sex | 30.9% | 31.9% | 31.5% |
|  |  | % of Total | 13.5% | 18.0% | 31.5% |
|  | No | Count | 217 | 277 | 494 |
|  |  | % within Sex | 69.1% | 68.1% | 68.5% |
|  |  | % of Total | 30.1% | 38.4% | 68.5% |
| Total | | Count | 314 | 407 | 721 |
|  |  | % within Sex | 100.0% | 100.0% | 100.0% |
|  |  | % of Total | 43.6% | 56.4% | 100.0% |

| **Chi-Square Tests** | | | | | |
| --- | --- | --- | --- | --- | --- |
|  | Value | df | Asymptotic Significance (2-sided) | Exact Sig. (2-sided) | Exact Sig. (1-sided) |
| Pearson Chi-Square | .090^a^ | 1 | .764 |  |  |
| Continuity Correction^b^ | .048 | 1 | .826 |  |  |
| Likelihood Ratio | .091 | 1 | .763 |  |  |
| Fisher's Exact Test |  |  |  | .808 | .413 |
| Linear-by-Linear Association | .090 | 1 | .764 |  |  |
| N of Valid Cases | 721 |  |  |  |  |
| a. 0 cells (0.0%) have expected count less than 5. The minimum expected count is 98.86. | | | | | |
| b. Computed only for a 2x2 table | | | | | |

**Mental health * Grade category**

| **Crosstab** | | | | | |
| --- | --- | --- | --- | --- | --- |
|  | | | Grade category | | Total |
|  |  |  | 9-10 | 11-12 |  |
| Mental health | Yes | Count | 114 | 113 | 227 |
|  |  | % within Grade category | 29.5% | 33.8% | 31.5% |
|  |  | % of Total | 15.8% | 15.7% | 31.5% |
|  | No | Count | 273 | 221 | 494 |
|  |  | % within Grade category | 70.5% | 66.2% | 68.5% |
|  |  | % of Total | 37.9% | 30.7% | 68.5% |
| Total | | Count | 387 | 334 | 721 |
|  |  | % within Grade category | 100.0% | 100.0% | 100.0% |
|  |  | % of Total | 53.7% | 46.3% | 100.0% |

| **Chi-Square Tests** | | | | | |
| --- | --- | --- | --- | --- | --- |
|  | Value | df | Asymptotic Significance (2-sided) | Exact Sig. (2-sided) | Exact Sig. (1-sided) |
| Pearson Chi-Square | 1.591^a^ | 1 | .207 |  |  |
| Continuity Correction^b^ | 1.394 | 1 | .238 |  |  |
| Likelihood Ratio | 1.589 | 1 | .208 |  |  |
| Fisher's Exact Test |  |  |  | .228 | .119 |
| Linear-by-Linear Association | 1.589 | 1 | .208 |  |  |
| N of Valid Cases | 721 |  |  |  |  |
| a. 0 cells (0.0%) have expected count less than 5. The minimum expected count is 105.16. | | | | | |
| b. Computed only for a 2x2 table | | | | | |

**Mental health * School type**

| **Crosstab** | | | | | |
| --- | --- | --- | --- | --- | --- |
|  | | | School type | | Total |
|  |  |  | Public | Private |  |
| Mental health | Yes | Count | 120 | 107 | 227 |
|  |  | % within School type | 28.6% | 35.4% | 31.5% |
|  |  | % of Total | 16.6% | 14.8% | 31.5% |
|  | No | Count | 299 | 195 | 494 |
|  |  | % within School type | 71.4% | 64.6% | 68.5% |
|  |  | % of Total | 41.5% | 27.0% | 68.5% |
| Total | | Count | 419 | 302 | 721 |
|  |  | % within School type | 100.0% | 100.0% | 100.0% |
|  |  | % of Total | 58.1% | 41.9% | 100.0% |

| **Chi-Square Tests** | | | | | |
| --- | --- | --- | --- | --- | --- |
|  | Value | df | Asymptotic Significance (2-sided) | Exact Sig. (2-sided) | Exact Sig. (1-sided) |
| Pearson Chi-Square | 3.752^a^ | 1 | .053 |  |  |
| Continuity Correction^b^ | 3.444 | 1 | .063 |  |  |
| Likelihood Ratio | 3.734 | 1 | .053 |  |  |
| Fisher's Exact Test |  |  |  | .062 | .032 |
| Linear-by-Linear Association | 3.747 | 1 | .053 |  |  |
| N of Valid Cases | 721 |  |  |  |  |
| a. 0 cells (0.0%) have expected count less than 5. The minimum expected count is 95.08. | | | | | |
| b. Computed only for a 2x2 table | | | | | |

**Nutrition * Sex**

| **Crosstab** | | | | | |
| --- | --- | --- | --- | --- | --- |
|  | | | Sex | | Total |
|  |  |  | Male | Female |  |
| Nutrition | Yes | Count | 122 | 163 | 285 |
|  |  | % within Sex | 38.7% | 40.0% | 39.5% |
|  |  | % of Total | 16.9% | 22.6% | 39.5% |
|  | No | Count | 193 | 244 | 437 |
|  |  | % within Sex | 61.3% | 60.0% | 60.5% |
|  |  | % of Total | 26.7% | 33.8% | 60.5% |
| Total | | Count | 315 | 407 | 722 |
|  |  | % within Sex | 100.0% | 100.0% | 100.0% |
|  |  | % of Total | 43.6% | 56.4% | 100.0% |

| **Chi-Square Tests** | | | | | |
| --- | --- | --- | --- | --- | --- |
|  | Value | df | Asymptotic Significance (2-sided) | Exact Sig. (2-sided) | Exact Sig. (1-sided) |
| Pearson Chi-Square | .129^a^ | 1 | .719 |  |  |
| Continuity Correction^b^ | .080 | 1 | .777 |  |  |
| Likelihood Ratio | .129 | 1 | .719 |  |  |
| Fisher's Exact Test |  |  |  | .759 | .389 |
| Linear-by-Linear Association | .129 | 1 | .719 |  |  |
| N of Valid Cases | 722 |  |  |  |  |
| a. 0 cells (0.0%) have expected count less than 5. The minimum expected count is 124.34. | | | | | |
| b. Computed only for a 2x2 table | | | | | |

**Nutrition * Grade category**

| **Crosstab** | | | | | |
| --- | --- | --- | --- | --- | --- |
|  | | | Grade category | | Total |
|  |  |  | 9-10 | 11-12 |  |
| Nutrition | Yes | Count | 141 | 144 | 285 |
|  |  | % within Grade category | 36.4% | 43.0% | 39.5% |
|  |  | % of Total | 19.5% | 19.9% | 39.5% |
|  | No | Count | 246 | 191 | 437 |
|  |  | % within Grade category | 63.6% | 57.0% | 60.5% |
|  |  | % of Total | 34.1% | 26.5% | 60.5% |
| Total | | Count | 387 | 335 | 722 |
|  |  | % within Grade category | 100.0% | 100.0% | 100.0% |
|  |  | % of Total | 53.6% | 46.4% | 100.0% |

| **Chi-Square Tests** | | | | | |
| --- | --- | --- | --- | --- | --- |
|  | Value | df | Asymptotic Significance (2-sided) | Exact Sig. (2-sided) | Exact Sig. (1-sided) |
| Pearson Chi-Square | 3.225^a^ | 1 | .073 |  |  |
| Continuity Correction^b^ | 2.957 | 1 | .086 |  |  |
| Likelihood Ratio | 3.224 | 1 | .073 |  |  |
| Fisher's Exact Test |  |  |  | .079 | .043 |
| Linear-by-Linear Association | 3.221 | 1 | .073 |  |  |
| N of Valid Cases | 722 |  |  |  |  |
| a. 0 cells (0.0%) have expected count less than 5. The minimum expected count is 132.24. | | | | | |
| b. Computed only for a 2x2 table | | | | | |

**Nutrition * School type**

| **Crosstab** | | | | | |
| --- | --- | --- | --- | --- | --- |
|  | | | School type | | Total |
|  |  |  | Public | Private |  |
| Nutrition | Yes | Count | 139 | 146 | 285 |
|  |  | % within School type | 33.1% | 48.3% | 39.5% |
|  |  | % of Total | 19.3% | 20.2% | 39.5% |
|  | No | Count | 281 | 156 | 437 |
|  |  | % within School type | 66.9% | 51.7% | 60.5% |
|  |  | % of Total | 38.9% | 21.6% | 60.5% |
| Total | | Count | 420 | 302 | 722 |
|  |  | % within School type | 100.0% | 100.0% | 100.0% |
|  |  | % of Total | 58.2% | 41.8% | 100.0% |

| **Chi-Square Tests** | | | | | |
| --- | --- | --- | --- | --- | --- |
|  | Value | df | Asymptotic Significance (2-sided) | Exact Sig. (2-sided) | Exact Sig. (1-sided) |
| Pearson Chi-Square | 17.098^a^ | 1 | .000 |  |  |
| Continuity Correction^b^ | 16.466 | 1 | .000 |  |  |
| Likelihood Ratio | 17.059 | 1 | .000 |  |  |
| Fisher's Exact Test |  |  |  | .000 | .000 |
| Linear-by-Linear Association | 17.075 | 1 | .000 |  |  |
| N of Valid Cases | 722 |  |  |  |  |
| a. 0 cells (0.0%) have expected count less than 5. The minimum expected count is 119.21. | | | | | |
| b. Computed only for a 2x2 table | | | | | |

**Smoking issue * Sex**

| **Crosstab** | | | | | |
| --- | --- | --- | --- | --- | --- |
|  | | | Sex | | Total |
|  |  |  | Male | Female |  |
| Smoking issue | Yes | Count | 117 | 149 | 266 |
|  |  | % within Sex | 37.1% | 36.6% | 36.8% |
|  |  | % of Total | 16.2% | 20.6% | 36.8% |
|  | No | Count | 198 | 258 | 456 |
|  |  | % within Sex | 62.9% | 63.4% | 63.2% |
|  |  | % of Total | 27.4% | 35.7% | 63.2% |
| Total | | Count | 315 | 407 | 722 |
|  |  | % within Sex | 100.0% | 100.0% | 100.0% |
|  |  | % of Total | 43.6% | 56.4% | 100.0% |

| **Chi-Square Tests** | | | | | |
| --- | --- | --- | --- | --- | --- |
|  | Value | df | Asymptotic Significance (2-sided) | Exact Sig. (2-sided) | Exact Sig. (1-sided) |
| Pearson Chi-Square | .022^a^ | 1 | .883 |  |  |
| Continuity Correction^b^ | .005 | 1 | .945 |  |  |
| Likelihood Ratio | .022 | 1 | .883 |  |  |
| Fisher's Exact Test |  |  |  | .938 | .472 |
| Linear-by-Linear Association | .022 | 1 | .883 |  |  |
| N of Valid Cases | 722 |  |  |  |  |
| a. 0 cells (0.0%) have expected count less than 5. The minimum expected count is 116.05. | | | | | |
| b. Computed only for a 2x2 table | | | | | |

**Smoking issue * Grade category**

| **Crosstab** | | | | | |
| --- | --- | --- | --- | --- | --- |
|  | | | Grade category | | Total |
|  |  |  | 9-10 | 11-12 |  |
| Smoking issue | Yes | Count | 148 | 118 | 266 |
|  |  | % within Grade category | 38.2% | 35.2% | 36.8% |
|  |  | % of Total | 20.5% | 16.3% | 36.8% |
|  | No | Count | 239 | 217 | 456 |
|  |  | % within Grade category | 61.8% | 64.8% | 63.2% |
|  |  | % of Total | 33.1% | 30.1% | 63.2% |
| Total | | Count | 387 | 335 | 722 |
|  |  | % within Grade category | 100.0% | 100.0% | 100.0% |
|  |  | % of Total | 53.6% | 46.4% | 100.0% |

| **Chi-Square Tests** | | | | | |
| --- | --- | --- | --- | --- | --- |
|  | Value | df | Asymptotic Significance (2-sided) | Exact Sig. (2-sided) | Exact Sig. (1-sided) |
| Pearson Chi-Square | .703^a^ | 1 | .402 |  |  |
| Continuity Correction^b^ | .580 | 1 | .446 |  |  |
| Likelihood Ratio | .704 | 1 | .401 |  |  |
| Fisher's Exact Test |  |  |  | .439 | .223 |
| Linear-by-Linear Association | .702 | 1 | .402 |  |  |
| N of Valid Cases | 722 |  |  |  |  |
| a. 0 cells (0.0%) have expected count less than 5. The minimum expected count is 123.42. | | | | | |
| b. Computed only for a 2x2 table | | | | | |

**Smoking issue * School type**

| **Crosstab** | | | | | |
| --- | --- | --- | --- | --- | --- |
|  | | | School type | | Total |
|  |  |  | Public | Private |  |
| Smoking issue | Yes | Count | 132 | 134 | 266 |
|  |  | % within School type | 31.4% | 44.4% | 36.8% |
|  |  | % of Total | 18.3% | 18.6% | 36.8% |
|  | No | Count | 288 | 168 | 456 |
|  |  | % within School type | 68.6% | 55.6% | 63.2% |
|  |  | % of Total | 39.9% | 23.3% | 63.2% |
| Total | | Count | 420 | 302 | 722 |
|  |  | % within School type | 100.0% | 100.0% | 100.0% |
|  |  | % of Total | 58.2% | 41.8% | 100.0% |

| **Chi-Square Tests** | | | | | |
| --- | --- | --- | --- | --- | --- |
|  | Value | df | Asymptotic Significance (2-sided) | Exact Sig. (2-sided) | Exact Sig. (1-sided) |
| Pearson Chi-Square | 12.646^a^ | 1 | .000 |  |  |
| Continuity Correction^b^ | 12.096 | 1 | .001 |  |  |
| Likelihood Ratio | 12.597 | 1 | .000 |  |  |
| Fisher's Exact Test |  |  |  | .000 | .000 |
| Linear-by-Linear Association | 12.629 | 1 | .000 |  |  |
| N of Valid Cases | 722 |  |  |  |  |
| a. 0 cells (0.0%) have expected count less than 5. The minimum expected count is 111.26. | | | | | |
| b. Computed only for a 2x2 table | | | | | |

**Alcohol use issue * Sex**

| **Crosstab** | | | | | |
| --- | --- | --- | --- | --- | --- |
|  | | | Sex | | Total |
|  |  |  | Male | Female |  |
| Alcohol use issue | Yes | Count | 113 | 135 | 248 |
|  |  | % within Sex | 35.9% | 33.2% | 34.3% |
|  |  | % of Total | 15.7% | 18.7% | 34.3% |
|  | No | Count | 202 | 272 | 474 |
|  |  | % within Sex | 64.1% | 66.8% | 65.7% |
|  |  | % of Total | 28.0% | 37.7% | 65.7% |
| Total | | Count | 315 | 407 | 722 |
|  |  | % within Sex | 100.0% | 100.0% | 100.0% |
|  |  | % of Total | 43.6% | 56.4% | 100.0% |

| **Chi-Square Tests** | | | | | |
| --- | --- | --- | --- | --- | --- |
|  | Value | df | Asymptotic Significance (2-sided) | Exact Sig. (2-sided) | Exact Sig. (1-sided) |
| Pearson Chi-Square | .576^a^ | 1 | .448 |  |  |
| Continuity Correction^b^ | .462 | 1 | .497 |  |  |
| Likelihood Ratio | .575 | 1 | .448 |  |  |
| Fisher's Exact Test |  |  |  | .477 | .248 |
| Linear-by-Linear Association | .575 | 1 | .448 |  |  |
| N of Valid Cases | 722 |  |  |  |  |
| a. 0 cells (0.0%) have expected count less than 5. The minimum expected count is 108.20. | | | | | |
| b. Computed only for a 2x2 table | | | | | |

**Alcohol use issue * Grade category**

| **Crosstab** | | | | | |
| --- | --- | --- | --- | --- | --- |
|  | | | Grade category | | Total |
|  |  |  | 9-10 | 11-12 |  |
| Alcohol use issue | Yes | Count | 128 | 120 | 248 |
|  |  | % within Grade category | 33.1% | 35.8% | 34.3% |
|  |  | % of Total | 17.7% | 16.6% | 34.3% |
|  | No | Count | 259 | 215 | 474 |
|  |  | % within Grade category | 66.9% | 64.2% | 65.7% |
|  |  | % of Total | 35.9% | 29.8% | 65.7% |
| Total | | Count | 387 | 335 | 722 |
|  |  | % within Grade category | 100.0% | 100.0% | 100.0% |
|  |  | % of Total | 53.6% | 46.4% | 100.0% |

| **Chi-Square Tests** | | | | | |
| --- | --- | --- | --- | --- | --- |
|  | Value | df | Asymptotic Significance (2-sided) | Exact Sig. (2-sided) | Exact Sig. (1-sided) |
| Pearson Chi-Square | .600^a^ | 1 | .438 |  |  |
| Continuity Correction^b^ | .485 | 1 | .486 |  |  |
| Likelihood Ratio | .600 | 1 | .439 |  |  |
| Fisher's Exact Test |  |  |  | .480 | .243 |
| Linear-by-Linear Association | .600 | 1 | .439 |  |  |
| N of Valid Cases | 722 |  |  |  |  |
| a. 0 cells (0.0%) have expected count less than 5. The minimum expected count is 115.07. | | | | | |
| b. Computed only for a 2x2 table | | | | | |

**Alcohol use issue * School type**

| **Crosstab** | | | | | |
| --- | --- | --- | --- | --- | --- |
|  | | | School type | | Total |
|  |  |  | Public | Private |  |
| Alcohol use issue | Yes | Count | 125 | 123 | 248 |
|  |  | % within School type | 29.8% | 40.7% | 34.3% |
|  |  | % of Total | 17.3% | 17.0% | 34.3% |
|  | No | Count | 295 | 179 | 474 |
|  |  | % within School type | 70.2% | 59.3% | 65.7% |
|  |  | % of Total | 40.9% | 24.8% | 65.7% |
| Total | | Count | 420 | 302 | 722 |
|  |  | % within School type | 100.0% | 100.0% | 100.0% |
|  |  | % of Total | 58.2% | 41.8% | 100.0% |

| **Chi-Square Tests** | | | | | |
| --- | --- | --- | --- | --- | --- |
|  | Value | df | Asymptotic Significance (2-sided) | Exact Sig. (2-sided) | Exact Sig. (1-sided) |
| Pearson Chi-Square | 9.369^a^ | 1 | .002 |  |  |
| Continuity Correction^b^ | 8.889 | 1 | .003 |  |  |
| Likelihood Ratio | 9.323 | 1 | .002 |  |  |
| Fisher's Exact Test |  |  |  | .003 | .001 |
| Linear-by-Linear Association | 9.356 | 1 | .002 |  |  |
| N of Valid Cases | 722 |  |  |  |  |
| a. 0 cells (0.0%) have expected count less than 5. The minimum expected count is 103.73. | | | | | |
| b. Computed only for a 2x2 table | | | | | |

**Physical activity * Sex**

| **Crosstab** | | | | | |
| --- | --- | --- | --- | --- | --- |
|  | | | Sex | | Total |
|  |  |  | Male | Female |  |
| Physical activity | Yes | Count | 157 | 145 | 302 |
|  |  | % within Sex | 49.8% | 35.6% | 41.8% |
|  |  | % of Total | 21.7% | 20.1% | 41.8% |
|  | No | Count | 158 | 262 | 420 |
|  |  | % within Sex | 50.2% | 64.4% | 58.2% |
|  |  | % of Total | 21.9% | 36.3% | 58.2% |
| Total | | Count | 315 | 407 | 722 |
|  |  | % within Sex | 100.0% | 100.0% | 100.0% |
|  |  | % of Total | 43.6% | 56.4% | 100.0% |

| **Chi-Square Tests** | | | | | |
| --- | --- | --- | --- | --- | --- |
|  | Value | df | Asymptotic Significance (2-sided) | Exact Sig. (2-sided) | Exact Sig. (1-sided) |
| Pearson Chi-Square | 14.746^a^ | 1 | .000 |  |  |
| Continuity Correction^b^ | 14.167 | 1 | .000 |  |  |
| Likelihood Ratio | 14.744 | 1 | .000 |  |  |
| Fisher's Exact Test |  |  |  | .000 | .000 |
| Linear-by-Linear Association | 14.725 | 1 | .000 |  |  |
| N of Valid Cases | 722 |  |  |  |  |
| a. 0 cells (0.0%) have expected count less than 5. The minimum expected count is 131.76. | | | | | |
| b. Computed only for a 2x2 table | | | | | |

**Physical activity * Grade category**

| **Crosstab** | | | | | |
| --- | --- | --- | --- | --- | --- |
|  | | | Grade category | | Total |
|  |  |  | 9-10 | 11-12 |  |
| Physical activity | Yes | Count | 147 | 155 | 302 |
|  |  | % within Grade category | 38.0% | 46.3% | 41.8% |
|  |  | % of Total | 20.4% | 21.5% | 41.8% |
|  | No | Count | 240 | 180 | 420 |
|  |  | % within Grade category | 62.0% | 53.7% | 58.2% |
|  |  | % of Total | 33.2% | 24.9% | 58.2% |
| Total | | Count | 387 | 335 | 722 |
|  |  | % within Grade category | 100.0% | 100.0% | 100.0% |
|  |  | % of Total | 53.6% | 46.4% | 100.0% |

| **Chi-Square Tests** | | | | | |
| --- | --- | --- | --- | --- | --- |
|  | Value | df | Asymptotic Significance (2-sided) | Exact Sig. (2-sided) | Exact Sig. (1-sided) |
| Pearson Chi-Square | 5.064^a^ | 1 | .024 |  |  |
| Continuity Correction^b^ | 4.730 | 1 | .030 |  |  |
| Likelihood Ratio | 5.064 | 1 | .024 |  |  |
| Fisher's Exact Test |  |  |  | .028 | .015 |
| Linear-by-Linear Association | 5.057 | 1 | .025 |  |  |
| N of Valid Cases | 722 |  |  |  |  |
| a. 0 cells (0.0%) have expected count less than 5. The minimum expected count is 140.12. | | | | | |
| b. Computed only for a 2x2 table | | | | | |

**Physical activity * School type**

| **Crosstab** | | | | | |
| --- | --- | --- | --- | --- | --- |
|  | | | School type | | Total |
|  |  |  | Public | Private |  |
| Physical activity | Yes | Count | 161 | 141 | 302 |
|  |  | % within School type | 38.3% | 46.7% | 41.8% |
|  |  | % of Total | 22.3% | 19.5% | 41.8% |
|  | No | Count | 259 | 161 | 420 |
|  |  | % within School type | 61.7% | 53.3% | 58.2% |
|  |  | % of Total | 35.9% | 22.3% | 58.2% |
| Total | | Count | 420 | 302 | 722 |
|  |  | % within School type | 100.0% | 100.0% | 100.0% |
|  |  | % of Total | 58.2% | 41.8% | 100.0% |

| **Chi-Square Tests** | | | | | |
| --- | --- | --- | --- | --- | --- |
|  | Value | df | Asymptotic Significance (2-sided) | Exact Sig. (2-sided) | Exact Sig. (1-sided) |
| Pearson Chi-Square | 5.040^a^ | 1 | .025 |  |  |
| Continuity Correction^b^ | 4.703 | 1 | .030 |  |  |
| Likelihood Ratio | 5.032 | 1 | .025 |  |  |
| Fisher's Exact Test |  |  |  | .027 | .015 |
| Linear-by-Linear Association | 5.034 | 1 | .025 |  |  |
| N of Valid Cases | 722 |  |  |  |  |
| a. 0 cells (0.0%) have expected count less than 5. The minimum expected count is 126.32. | | | | | |
| b. Computed only for a 2x2 table | | | | | |

**Oral health * Sex**

| **Crosstab** | | | | | |
| --- | --- | --- | --- | --- | --- |
|  | | | Sex | | Total |
|  |  |  | Male | Female |  |
| Oral health | Yes | Count | 69 | 90 | 159 |
|  |  | % within Sex | 22.0% | 22.1% | 22.1% |
|  |  | % of Total | 9.6% | 12.5% | 22.1% |
|  | No | Count | 245 | 317 | 562 |
|  |  | % within Sex | 78.0% | 77.9% | 77.9% |
|  |  | % of Total | 34.0% | 44.0% | 77.9% |
| Total | | Count | 314 | 407 | 721 |
|  |  | % within Sex | 100.0% | 100.0% | 100.0% |
|  |  | % of Total | 43.6% | 56.4% | 100.0% |

| **Chi-Square Tests** | | | | | |
| --- | --- | --- | --- | --- | --- |
|  | Value | df | Asymptotic Significance (2-sided) | Exact Sig. (2-sided) | Exact Sig. (1-sided) |
| Pearson Chi-Square | .002^a^ | 1 | .965 |  |  |
| Continuity Correction^b^ | .000 | 1 | 1.000 |  |  |
| Likelihood Ratio | .002 | 1 | .965 |  |  |
| Fisher's Exact Test |  |  |  | 1.000 | .519 |
| Linear-by-Linear Association | .002 | 1 | .965 |  |  |
| N of Valid Cases | 721 |  |  |  |  |
| a. 0 cells (0.0%) have expected count less than 5. The minimum expected count is 69.25. | | | | | |
| b. Computed only for a 2x2 table | | | | | |

**Oral health * Grade category**

| **Crosstab** | | | | | |
| --- | --- | --- | --- | --- | --- |
|  | | | Grade category | | Total |
|  |  |  | 9-10 | 11-12 |  |
| Oral health | Yes | Count | 79 | 80 | 159 |
|  |  | % within Grade category | 20.4% | 24.0% | 22.1% |
|  |  | % of Total | 11.0% | 11.1% | 22.1% |
|  | No | Count | 308 | 254 | 562 |
|  |  | % within Grade category | 79.6% | 76.0% | 77.9% |
|  |  | % of Total | 42.7% | 35.2% | 77.9% |
| Total | | Count | 387 | 334 | 721 |
|  |  | % within Grade category | 100.0% | 100.0% | 100.0% |
|  |  | % of Total | 53.7% | 46.3% | 100.0% |

| **Chi-Square Tests** | | | | | |
| --- | --- | --- | --- | --- | --- |
|  | Value | df | Asymptotic Significance (2-sided) | Exact Sig. (2-sided) | Exact Sig. (1-sided) |
| Pearson Chi-Square | 1.306^a^ | 1 | .253 |  |  |
| Continuity Correction^b^ | 1.108 | 1 | .292 |  |  |
| Likelihood Ratio | 1.303 | 1 | .254 |  |  |
| Fisher's Exact Test |  |  |  | .280 | .146 |
| Linear-by-Linear Association | 1.304 | 1 | .253 |  |  |
| N of Valid Cases | 721 |  |  |  |  |
| a. 0 cells (0.0%) have expected count less than 5. The minimum expected count is 73.66. | | | | | |
| b. Computed only for a 2x2 table | | | | | |

**Oral health * School type**

| **Crosstab** | | | | | |
| --- | --- | --- | --- | --- | --- |
|  | | | School type | | Total |
|  |  |  | Public | Private |  |
| Oral health | Yes | Count | 74 | 85 | 159 |
|  |  | % within School type | 17.7% | 28.1% | 22.1% |
|  |  | % of Total | 10.3% | 11.8% | 22.1% |
|  | No | Count | 345 | 217 | 562 |
|  |  | % within School type | 82.3% | 71.9% | 77.9% |
|  |  | % of Total | 47.9% | 30.1% | 77.9% |
| Total | | Count | 419 | 302 | 721 |
|  |  | % within School type | 100.0% | 100.0% | 100.0% |
|  |  | % of Total | 58.1% | 41.9% | 100.0% |

| **Chi-Square Tests** | | | | | |
| --- | --- | --- | --- | --- | --- |
|  | Value | df | Asymptotic Significance (2-sided) | Exact Sig. (2-sided) | Exact Sig. (1-sided) |
| Pearson Chi-Square | 11.223^a^ | 1 | .001 |  |  |
| Continuity Correction^b^ | 10.622 | 1 | .001 |  |  |
| Likelihood Ratio | 11.102 | 1 | .001 |  |  |
| Fisher's Exact Test |  |  |  | .001 | .001 |
| Linear-by-Linear Association | 11.208 | 1 | .001 |  |  |
| N of Valid Cases | 721 |  |  |  |  |
| a. 0 cells (0.0%) have expected count less than 5. The minimum expected count is 66.60. | | | | | |
| b. Computed only for a 2x2 table | | | | | |

**7. Sources of the health information or topic learned:** From where have you got or learned this information?

**School/teachers * Sex**

| **Crosstab** | | | | | |
| --- | --- | --- | --- | --- | --- |
|  | | | Sex | | Total |
|  |  |  | Male | Female |  |
| School/teachers | Yes | Count | 299 | 397 | 696 |
|  |  | % within Sex | 94.9% | 97.5% | 96.4% |
|  |  | % of Total | 41.4% | 55.0% | 96.4% |
|  | No | Count | 16 | 10 | 26 |
|  |  | % within Sex | 5.1% | 2.5% | 3.6% |
|  |  | % of Total | 2.2% | 1.4% | 3.6% |
| Total | | Count | 315 | 407 | 722 |
|  |  | % within Sex | 100.0% | 100.0% | 100.0% |
|  |  | % of Total | 43.6% | 56.4% | 100.0% |

| **Chi-Square Tests** | | | | | |
| --- | --- | --- | --- | --- | --- |
|  | Value | df | Asymptotic Significance (2-sided) | Exact Sig. (2-sided) | Exact Sig. (1-sided) |
| Pearson Chi-Square | 3.518^a^ | 1 | .061 |  |  |
| Continuity Correction^b^ | 2.803 | 1 | .094 |  |  |
| Likelihood Ratio | 3.487 | 1 | .062 |  |  |
| Fisher's Exact Test |  |  |  | .071 | .048 |
| Linear-by-Linear Association | 3.513 | 1 | .061 |  |  |
| N of Valid Cases | 722 |  |  |  |  |
| a. 0 cells (0.0%) have expected count less than 5. The minimum expected count is 11.34. | | | | | |
| b. Computed only for a 2x2 table | | | | | |

**School/teachers * Grade category**

| **Crosstab** | | | | | |
| --- | --- | --- | --- | --- | --- |
|  | | | Grade category | | Total |
|  |  |  | 9-10 | 11-12 |  |
| School/teachers | Yes | Count | 372 | 324 | 696 |
|  |  | % within Grade category | 96.1% | 96.7% | 96.4% |
|  |  | % of Total | 51.5% | 44.9% | 96.4% |
|  | No | Count | 15 | 11 | 26 |
|  |  | % within Grade category | 3.9% | 3.3% | 3.6% |
|  |  | % of Total | 2.1% | 1.5% | 3.6% |
| Total | | Count | 387 | 335 | 722 |
|  |  | % within Grade category | 100.0% | 100.0% | 100.0% |
|  |  | % of Total | 53.6% | 46.4% | 100.0% |

| **Chi-Square Tests** | | | | | |
| --- | --- | --- | --- | --- | --- |
|  | Value | df | Asymptotic Significance (2-sided) | Exact Sig. (2-sided) | Exact Sig. (1-sided) |
| Pearson Chi-Square | .182^a^ | 1 | .670 |  |  |
| Continuity Correction^b^ | .051 | 1 | .821 |  |  |
| Likelihood Ratio | .182 | 1 | .669 |  |  |
| Fisher's Exact Test |  |  |  | .695 | .413 |
| Linear-by-Linear Association | .181 | 1 | .670 |  |  |
| N of Valid Cases | 722 |  |  |  |  |
| a. 0 cells (0.0%) have expected count less than 5. The minimum expected count is 12.06. | | | | | |
| b. Computed only for a 2x2 table | | | | | |

**School/teachers * School type**

| **Crosstab** | | | | | |
| --- | --- | --- | --- | --- | --- |
|  | | | School type | | Total |
|  |  |  | Public | Private |  |
| School/teachers | Yes | Count | 403 | 293 | 696 |
|  |  | % within School type | 96.0% | 97.0% | 96.4% |
|  |  | % of Total | 55.8% | 40.6% | 96.4% |
|  | No | Count | 17 | 9 | 26 |
|  |  | % within School type | 4.0% | 3.0% | 3.6% |
|  |  | % of Total | 2.4% | 1.2% | 3.6% |
| Total | | Count | 420 | 302 | 722 |
|  |  | % within School type | 100.0% | 100.0% | 100.0% |
|  |  | % of Total | 58.2% | 41.8% | 100.0% |

| **Chi-Square Tests** | | | | | |
| --- | --- | --- | --- | --- | --- |
|  | Value | df | Asymptotic Significance (2-sided) | Exact Sig. (2-sided) | Exact Sig. (1-sided) |
| Pearson Chi-Square | .577^a^ | 1 | .448 |  |  |
| Continuity Correction^b^ | .310 | 1 | .578 |  |  |
| Likelihood Ratio | .588 | 1 | .443 |  |  |
| Fisher's Exact Test |  |  |  | .545 | .292 |
| Linear-by-Linear Association | .576 | 1 | .448 |  |  |
| N of Valid Cases | 722 |  |  |  |  |
| a. 0 cells (0.0%) have expected count less than 5. The minimum expected count is 10.88. | | | | | |
| b. Computed only for a 2x2 table | | | | | |

**Television/radio * Sex**

| **Crosstab** | | | | | |
| --- | --- | --- | --- | --- | --- |
|  | | | Sex | | Total |
|  |  |  | Male | Female |  |
| Television/radio | Yes | Count | 129 | 188 | 317 |
|  |  | % within Sex | 41.0% | 46.2% | 43.9% |
|  |  | % of Total | 17.9% | 26.0% | 43.9% |
|  | No | Count | 186 | 219 | 405 |
|  |  | % within Sex | 59.0% | 53.8% | 56.1% |
|  |  | % of Total | 25.8% | 30.3% | 56.1% |
| Total | | Count | 315 | 407 | 722 |
|  |  | % within Sex | 100.0% | 100.0% | 100.0% |
|  |  | % of Total | 43.6% | 56.4% | 100.0% |

| **Chi-Square Tests** | | | | | |
| --- | --- | --- | --- | --- | --- |
|  | Value | df | Asymptotic Significance (2-sided) | Exact Sig. (2-sided) | Exact Sig. (1-sided) |
| Pearson Chi-Square | 1.979^a^ | 1 | .159 |  |  |
| Continuity Correction^b^ | 1.772 | 1 | .183 |  |  |
| Likelihood Ratio | 1.982 | 1 | .159 |  |  |
| Fisher's Exact Test |  |  |  | .174 | .091 |
| Linear-by-Linear Association | 1.976 | 1 | .160 |  |  |
| N of Valid Cases | 722 |  |  |  |  |
| a. 0 cells (0.0%) have expected count less than 5. The minimum expected count is 138.30. | | | | | |
| b. Computed only for a 2x2 table | | | | | |

**Television/radio * Grade category**

| **Crosstab** | | | | | |
| --- | --- | --- | --- | --- | --- |
|  | | | Grade category | | Total |
|  |  |  | 9-10 | 11-12 |  |
| Television/radio | Yes | Count | 161 | 156 | 317 |
|  |  | % within Grade category | 41.6% | 46.6% | 43.9% |
|  |  | % of Total | 22.3% | 21.6% | 43.9% |
|  | No | Count | 226 | 179 | 405 |
|  |  | % within Grade category | 58.4% | 53.4% | 56.1% |
|  |  | % of Total | 31.3% | 24.8% | 56.1% |
| Total | | Count | 387 | 335 | 722 |
|  |  | % within Grade category | 100.0% | 100.0% | 100.0% |
|  |  | % of Total | 53.6% | 46.4% | 100.0% |

| **Chi-Square Tests** | | | | | |
| --- | --- | --- | --- | --- | --- |
|  | Value | df | Asymptotic Significance (2-sided) | Exact Sig. (2-sided) | Exact Sig. (1-sided) |
| Pearson Chi-Square | 1.797^a^ | 1 | .180 |  |  |
| Continuity Correction^b^ | 1.601 | 1 | .206 |  |  |
| Likelihood Ratio | 1.797 | 1 | .180 |  |  |
| Fisher's Exact Test |  |  |  | .201 | .103 |
| Linear-by-Linear Association | 1.795 | 1 | .180 |  |  |
| N of Valid Cases | 722 |  |  |  |  |
| a. 0 cells (0.0%) have expected count less than 5. The minimum expected count is 147.08. | | | | | |
| b. Computed only for a 2x2 table | | | | | |

**Television/radio * School type**

| **Crosstab** | | | | | |
| --- | --- | --- | --- | --- | --- |
|  | | | School type | | Total |
|  |  |  | Public | Private |  |
| Television/radio | Yes | Count | 173 | 144 | 317 |
|  |  | % within School type | 41.2% | 47.7% | 43.9% |
|  |  | % of Total | 24.0% | 19.9% | 43.9% |
|  | No | Count | 247 | 158 | 405 |
|  |  | % within School type | 58.8% | 52.3% | 56.1% |
|  |  | % of Total | 34.2% | 21.9% | 56.1% |
| Total | | Count | 420 | 302 | 722 |
|  |  | % within School type | 100.0% | 100.0% | 100.0% |
|  |  | % of Total | 58.2% | 41.8% | 100.0% |

| **Chi-Square Tests** | | | | | |
| --- | --- | --- | --- | --- | --- |
|  | Value | df | Asymptotic Significance (2-sided) | Exact Sig. (2-sided) | Exact Sig. (1-sided) |
| Pearson Chi-Square | 3.006^a^ | 1 | .083 |  |  |
| Continuity Correction^b^ | 2.748 | 1 | .097 |  |  |
| Likelihood Ratio | 3.003 | 1 | .083 |  |  |
| Fisher's Exact Test |  |  |  | .094 | .049 |
| Linear-by-Linear Association | 3.002 | 1 | .083 |  |  |
| N of Valid Cases | 722 |  |  |  |  |
| a. 0 cells (0.0%) have expected count less than 5. The minimum expected count is 132.60. | | | | | |
| b. Computed only for a 2x2 table | | | | | |

**Family * Sex**

| **Crosstab** | | | | | |
| --- | --- | --- | --- | --- | --- |
|  | | | Sex | | Total |
|  |  |  | Male | Female |  |
| Family | Yes | Count | 108 | 151 | 259 |
|  |  | % within Sex | 34.3% | 37.1% | 35.9% |
|  |  | % of Total | 15.0% | 20.9% | 35.9% |
|  | No | Count | 207 | 256 | 463 |
|  |  | % within Sex | 65.7% | 62.9% | 64.1% |
|  |  | % of Total | 28.7% | 35.5% | 64.1% |
| Total | | Count | 315 | 407 | 722 |
|  |  | % within Sex | 100.0% | 100.0% | 100.0% |
|  |  | % of Total | 43.6% | 56.4% | 100.0% |

| **Chi-Square Tests** | | | | | |
| --- | --- | --- | --- | --- | --- |
|  | Value | df | Asymptotic Significance (2-sided) | Exact Sig. (2-sided) | Exact Sig. (1-sided) |
| Pearson Chi-Square | .612^a^ | 1 | .434 |  |  |
| Continuity Correction^b^ | .495 | 1 | .482 |  |  |
| Likelihood Ratio | .613 | 1 | .434 |  |  |
| Fisher's Exact Test |  |  |  | .481 | .241 |
| Linear-by-Linear Association | .611 | 1 | .434 |  |  |
| N of Valid Cases | 722 |  |  |  |  |
| a. 0 cells (0.0%) have expected count less than 5. The minimum expected count is 113.00. | | | | | |
| b. Computed only for a 2x2 table | | | | | |

**Family * Grade category**

| **Crosstab** | | | | | |
| --- | --- | --- | --- | --- | --- |
|  | | | Grade category | | Total |
|  |  |  | 9-10 | 11-12 |  |
| Family | Yes | Count | 160 | 99 | 259 |
|  |  | % within Grade category | 41.3% | 29.6% | 35.9% |
|  |  | % of Total | 22.2% | 13.7% | 35.9% |
|  | No | Count | 227 | 236 | 463 |
|  |  | % within Grade category | 58.7% | 70.4% | 64.1% |
|  |  | % of Total | 31.4% | 32.7% | 64.1% |
| Total | | Count | 387 | 335 | 722 |
|  |  | % within Grade category | 100.0% | 100.0% | 100.0% |
|  |  | % of Total | 53.6% | 46.4% | 100.0% |

| **Chi-Square Tests** | | | | | |
| --- | --- | --- | --- | --- | --- |
|  | Value | df | Asymptotic Significance (2-sided) | Exact Sig. (2-sided) | Exact Sig. (1-sided) |
| Pearson Chi-Square | 10.853^a^ | 1 | .001 |  |  |
| Continuity Correction^b^ | 10.346 | 1 | .001 |  |  |
| Likelihood Ratio | 10.929 | 1 | .001 |  |  |
| Fisher's Exact Test |  |  |  | .001 | .001 |
| Linear-by-Linear Association | 10.838 | 1 | .001 |  |  |
| N of Valid Cases | 722 |  |  |  |  |
| a. 0 cells (0.0%) have expected count less than 5. The minimum expected count is 120.17. | | | | | |
| b. Computed only for a 2x2 table | | | | | |

**Family * School type**

| **Crosstab** | | | | | |
| --- | --- | --- | --- | --- | --- |
|  | | | School type | | Total |
|  |  |  | Public | Private |  |
| Family | Yes | Count | 122 | 137 | 259 |
|  |  | % within School type | 29.0% | 45.4% | 35.9% |
|  |  | % of Total | 16.9% | 19.0% | 35.9% |
|  | No | Count | 298 | 165 | 463 |
|  |  | % within School type | 71.0% | 54.6% | 64.1% |
|  |  | % of Total | 41.3% | 22.9% | 64.1% |
| Total | | Count | 420 | 302 | 722 |
|  |  | % within School type | 100.0% | 100.0% | 100.0% |
|  |  | % of Total | 58.2% | 41.8% | 100.0% |

| **Chi-Square Tests** | | | | | |
| --- | --- | --- | --- | --- | --- |
|  | Value | df | Asymptotic Significance (2-sided) | Exact Sig. (2-sided) | Exact Sig. (1-sided) |
| Pearson Chi-Square | 20.332^a^ | 1 | .000 |  |  |
| Continuity Correction^b^ | 19.629 | 1 | .000 |  |  |
| Likelihood Ratio | 20.246 | 1 | .000 |  |  |
| Fisher's Exact Test |  |  |  | .000 | .000 |
| Linear-by-Linear Association | 20.304 | 1 | .000 |  |  |
| N of Valid Cases | 722 |  |  |  |  |
| a. 0 cells (0.0%) have expected count less than 5. The minimum expected count is 108.34. | | | | | |
| b. Computed only for a 2x2 table | | | | | |

**Friends/peers * Sex**

| **Crosstab** | | | | | |
| --- | --- | --- | --- | --- | --- |
|  | | | Sex | | Total |
|  |  |  | Male | Female |  |
| Friends/peers | Yes | Count | 82 | 98 | 180 |
|  |  | % within Sex | 26.0% | 24.1% | 24.9% |
|  |  | % of Total | 11.4% | 13.6% | 24.9% |
|  | No | Count | 233 | 309 | 542 |
|  |  | % within Sex | 74.0% | 75.9% | 75.1% |
|  |  | % of Total | 32.3% | 42.8% | 75.1% |
| Total | | Count | 315 | 407 | 722 |
|  |  | % within Sex | 100.0% | 100.0% | 100.0% |
|  |  | % of Total | 43.6% | 56.4% | 100.0% |

| **Chi-Square Tests** | | | | | |
| --- | --- | --- | --- | --- | --- |
|  | Value | df | Asymptotic Significance (2-sided) | Exact Sig. (2-sided) | Exact Sig. (1-sided) |
| Pearson Chi-Square | .362^a^ | 1 | .547 |  |  |
| Continuity Correction^b^ | .265 | 1 | .607 |  |  |
| Likelihood Ratio | .361 | 1 | .548 |  |  |
| Fisher's Exact Test |  |  |  | .603 | .303 |
| Linear-by-Linear Association | .361 | 1 | .548 |  |  |
| N of Valid Cases | 722 |  |  |  |  |
| a. 0 cells (0.0%) have expected count less than 5. The minimum expected count is 78.53. | | | | | |
| b. Computed only for a 2x2 table | | | | | |

**Friends/peers * Grade category**

| **Crosstab** | | | | | |
| --- | --- | --- | --- | --- | --- |
|  | | | Grade category | | Total |
|  |  |  | 9-10 | 11-12 |  |
| Friends/peers | Yes | Count | 97 | 83 | 180 |
|  |  | % within Grade category | 25.1% | 24.8% | 24.9% |
|  |  | % of Total | 13.4% | 11.5% | 24.9% |
|  | No | Count | 290 | 252 | 542 |
|  |  | % within Grade category | 74.9% | 75.2% | 75.1% |
|  |  | % of Total | 40.2% | 34.9% | 75.1% |
| Total | | Count | 387 | 335 | 722 |
|  |  | % within Grade category | 100.0% | 100.0% | 100.0% |
|  |  | % of Total | 53.6% | 46.4% | 100.0% |

| **Chi-Square Tests** | | | | | |
| --- | --- | --- | --- | --- | --- |
|  | Value | df | Asymptotic Significance (2-sided) | Exact Sig. (2-sided) | Exact Sig. (1-sided) |
| Pearson Chi-Square | .008^a^ | 1 | .929 |  |  |
| Continuity Correction^b^ | .000 | 1 | .998 |  |  |
| Likelihood Ratio | .008 | 1 | .929 |  |  |
| Fisher's Exact Test |  |  |  | 1.000 | .499 |
| Linear-by-Linear Association | .008 | 1 | .929 |  |  |
| N of Valid Cases | 722 |  |  |  |  |
| a. 0 cells (0.0%) have expected count less than 5. The minimum expected count is 83.52. | | | | | |
| b. Computed only for a 2x2 table | | | | | |

**Friends/peers * School type**

| **Crosstab** | | | | | |
| --- | --- | --- | --- | --- | --- |
|  | | | School type | | Total |
|  |  |  | Public | Private |  |
| Friends/peers | Yes | Count | 106 | 74 | 180 |
|  |  | % within School type | 25.2% | 24.5% | 24.9% |
|  |  | % of Total | 14.7% | 10.2% | 24.9% |
|  | No | Count | 314 | 228 | 542 |
|  |  | % within School type | 74.8% | 75.5% | 75.1% |
|  |  | % of Total | 43.5% | 31.6% | 75.1% |
| Total | | Count | 420 | 302 | 722 |
|  |  | % within School type | 100.0% | 100.0% | 100.0% |
|  |  | % of Total | 58.2% | 41.8% | 100.0% |

| **Chi-Square Tests** | | | | | |
| --- | --- | --- | --- | --- | --- |
|  | Value | df | Asymptotic Significance (2-sided) | Exact Sig. (2-sided) | Exact Sig. (1-sided) |
| Pearson Chi-Square | .051^a^ | 1 | .822 |  |  |
| Continuity Correction^b^ | .019 | 1 | .890 |  |  |
| Likelihood Ratio | .051 | 1 | .822 |  |  |
| Fisher's Exact Test |  |  |  | .862 | .446 |
| Linear-by-Linear Association | .051 | 1 | .822 |  |  |
| N of Valid Cases | 722 |  |  |  |  |
| a. 0 cells (0.0%) have expected count less than 5. The minimum expected count is 75.29. | | | | | |
| b. Computed only for a 2x2 table | | | | | |

**Health professionals * Sex**

| **Crosstab** | | | | | |
| --- | --- | --- | --- | --- | --- |
|  | | | Sex | | Total |
|  |  |  | Male | Female |  |
| Health professionals | Yes | Count | 98 | 115 | 213 |
|  |  | % within Sex | 31.1% | 28.3% | 29.5% |
|  |  | % of Total | 13.6% | 15.9% | 29.5% |
|  | No | Count | 217 | 292 | 509 |
|  |  | % within Sex | 68.9% | 71.7% | 70.5% |
|  |  | % of Total | 30.1% | 40.4% | 70.5% |
| Total | | Count | 315 | 407 | 722 |
|  |  | % within Sex | 100.0% | 100.0% | 100.0% |
|  |  | % of Total | 43.6% | 56.4% | 100.0% |

| **Chi-Square Tests** | | | | | |
| --- | --- | --- | --- | --- | --- |
|  | Value | df | Asymptotic Significance (2-sided) | Exact Sig. (2-sided) | Exact Sig. (1-sided) |
| Pearson Chi-Square | .696^a^ | 1 | .404 |  |  |
| Continuity Correction^b^ | .566 | 1 | .452 |  |  |
| Likelihood Ratio | .695 | 1 | .405 |  |  |
| Fisher's Exact Test |  |  |  | .412 | .226 |
| Linear-by-Linear Association | .695 | 1 | .404 |  |  |
| N of Valid Cases | 722 |  |  |  |  |
| a. 0 cells (0.0%) have expected count less than 5. The minimum expected count is 92.93. | | | | | |
| b. Computed only for a 2x2 table | | | | | |

**Health professionals * Grade category**

| **Crosstab** | | | | | |
| --- | --- | --- | --- | --- | --- |
|  | | | Grade category | | Total |
|  |  |  | 9-10 | 11-12 |  |
| Health professionals | Yes | Count | 115 | 98 | 213 |
|  |  | % within Grade category | 29.7% | 29.3% | 29.5% |
|  |  | % of Total | 15.9% | 13.6% | 29.5% |
|  | No | Count | 272 | 237 | 509 |
|  |  | % within Grade category | 70.3% | 70.7% | 70.5% |
|  |  | % of Total | 37.7% | 32.8% | 70.5% |
| Total | | Count | 387 | 335 | 722 |
|  |  | % within Grade category | 100.0% | 100.0% | 100.0% |
|  |  | % of Total | 53.6% | 46.4% | 100.0% |

| **Chi-Square Tests** | | | | | |
| --- | --- | --- | --- | --- | --- |
|  | Value | df | Asymptotic Significance (2-sided) | Exact Sig. (2-sided) | Exact Sig. (1-sided) |
| Pearson Chi-Square | .018^a^ | 1 | .892 |  |  |
| Continuity Correction^b^ | .003 | 1 | .957 |  |  |
| Likelihood Ratio | .018 | 1 | .892 |  |  |
| Fisher's Exact Test |  |  |  | .935 | .479 |
| Linear-by-Linear Association | .018 | 1 | .892 |  |  |
| N of Valid Cases | 722 |  |  |  |  |
| a. 0 cells (0.0%) have expected count less than 5. The minimum expected count is 98.83. | | | | | |
| b. Computed only for a 2x2 table | | | | | |

**Health professionals * School type**

| **Crosstab** | | | | | |
| --- | --- | --- | --- | --- | --- |
|  | | | School type | | Total |
|  |  |  | Public | Private |  |
| Health professionals | Yes | Count | 110 | 103 | 213 |
|  |  | % within School type | 26.2% | 34.1% | 29.5% |
|  |  | % of Total | 15.2% | 14.3% | 29.5% |
|  | No | Count | 310 | 199 | 509 |
|  |  | % within School type | 73.8% | 65.9% | 70.5% |
|  |  | % of Total | 42.9% | 27.6% | 70.5% |
| Total | | Count | 420 | 302 | 722 |
|  |  | % within School type | 100.0% | 100.0% | 100.0% |
|  |  | % of Total | 58.2% | 41.8% | 100.0% |

| **Chi-Square Tests** | | | | | |
| --- | --- | --- | --- | --- | --- |
|  | Value | df | Asymptotic Significance (2-sided) | Exact Sig. (2-sided) | Exact Sig. (1-sided) |
| Pearson Chi-Square | 5.292^a^ | 1 | .021 |  |  |
| Continuity Correction^b^ | 4.919 | 1 | .027 |  |  |
| Likelihood Ratio | 5.260 | 1 | .022 |  |  |
| Fisher's Exact Test |  |  |  | .025 | .013 |
| Linear-by-Linear Association | 5.285 | 1 | .022 |  |  |
| N of Valid Cases | 722 |  |  |  |  |
| a. 0 cells (0.0%) have expected count less than 5. The minimum expected count is 89.09. | | | | | |
| b. Computed only for a 2x2 table | | | | | |

**Internet * Sex**

| **Crosstab** | | | | | |
| --- | --- | --- | --- | --- | --- |
|  | | | Sex | | Total |
|  |  |  | Male | Female |  |
| Internet | Yes | Count | 131 | 138 | 269 |
|  |  | % within Sex | 41.6% | 33.9% | 37.3% |
|  |  | % of Total | 18.1% | 19.1% | 37.3% |
|  | No | Count | 184 | 269 | 453 |
|  |  | % within Sex | 58.4% | 66.1% | 62.7% |
|  |  | % of Total | 25.5% | 37.3% | 62.7% |
| Total | | Count | 315 | 407 | 722 |
|  |  | % within Sex | 100.0% | 100.0% | 100.0% |
|  |  | % of Total | 43.6% | 56.4% | 100.0% |

| **Chi-Square Tests** | | | | | |
| --- | --- | --- | --- | --- | --- |
|  | Value | df | Asymptotic Significance (2-sided) | Exact Sig. (2-sided) | Exact Sig. (1-sided) |
| Pearson Chi-Square | 4.481^a^ | 1 | .034 |  |  |
| Continuity Correction^b^ | 4.159 | 1 | .041 |  |  |
| Likelihood Ratio | 4.471 | 1 | .034 |  |  |
| Fisher's Exact Test |  |  |  | .036 | .021 |
| Linear-by-Linear Association | 4.475 | 1 | .034 |  |  |
| N of Valid Cases | 722 |  |  |  |  |
| a. 0 cells (0.0%) have expected count less than 5. The minimum expected count is 117.36. | | | | | |
| b. Computed only for a 2x2 table | | | | | |

**Internet * Grade category**

| **Crosstab** | | | | | |
| --- | --- | --- | --- | --- | --- |
|  | | | Grade category | | Total |
|  |  |  | 9-10 | 11-12 |  |
| Internet | Yes | Count | 126 | 143 | 269 |
|  |  | % within Grade category | 32.6% | 42.7% | 37.3% |
|  |  | % of Total | 17.5% | 19.8% | 37.3% |
|  | No | Count | 261 | 192 | 453 |
|  |  | % within Grade category | 67.4% | 57.3% | 62.7% |
|  |  | % of Total | 36.1% | 26.6% | 62.7% |
| Total | | Count | 387 | 335 | 722 |
|  |  | % within Grade category | 100.0% | 100.0% | 100.0% |
|  |  | % of Total | 53.6% | 46.4% | 100.0% |

| **Chi-Square Tests** | | | | | |
| --- | --- | --- | --- | --- | --- |
|  | Value | df | Asymptotic Significance (2-sided) | Exact Sig. (2-sided) | Exact Sig. (1-sided) |
| Pearson Chi-Square | 7.880^a^ | 1 | .005 |  |  |
| Continuity Correction^b^ | 7.453 | 1 | .006 |  |  |
| Likelihood Ratio | 7.878 | 1 | .005 |  |  |
| Fisher's Exact Test |  |  |  | .005 | .003 |
| Linear-by-Linear Association | 7.869 | 1 | .005 |  |  |
| N of Valid Cases | 722 |  |  |  |  |
| a. 0 cells (0.0%) have expected count less than 5. The minimum expected count is 124.81. | | | | | |
| b. Computed only for a 2x2 table | | | | | |

**Internet * School type**

| **Crosstab** | | | | | |
| --- | --- | --- | --- | --- | --- |
|  | | | School type | | Total |
|  |  |  | Public | Private |  |
| Internet | Yes | Count | 117 | 152 | 269 |
|  |  | % within School type | 27.9% | 50.3% | 37.3% |
|  |  | % of Total | 16.2% | 21.1% | 37.3% |
|  | No | Count | 303 | 150 | 453 |
|  |  | % within School type | 72.1% | 49.7% | 62.7% |
|  |  | % of Total | 42.0% | 20.8% | 62.7% |
| Total | | Count | 420 | 302 | 722 |
|  |  | % within School type | 100.0% | 100.0% | 100.0% |
|  |  | % of Total | 58.2% | 41.8% | 100.0% |

| **Chi-Square Tests** | | | | | |
| --- | --- | --- | --- | --- | --- |
|  | Value | df | Asymptotic Significance (2-sided) | Exact Sig. (2-sided) | Exact Sig. (1-sided) |
| Pearson Chi-Square | 37.958^a^ | 1 | .000 |  |  |
| Continuity Correction^b^ | 37.003 | 1 | .000 |  |  |
| Likelihood Ratio | 37.900 | 1 | .000 |  |  |
| Fisher's Exact Test |  |  |  | .000 | .000 |
| Linear-by-Linear Association | 37.905 | 1 | .000 |  |  |
| N of Valid Cases | 722 |  |  |  |  |
| a. 0 cells (0.0%) have expected count less than 5. The minimum expected count is 112.52. | | | | | |
| b. Computed only for a 2x2 table | | | | | |

**Books * Sex**

| **Crosstab** | | | | | |
| --- | --- | --- | --- | --- | --- |
|  | | | Sex | | Total |
|  |  |  | Male | Female |  |
| Books | Yes | Count | 90 | 106 | 196 |
|  |  | % within Sex | 28.6% | 26.0% | 27.1% |
|  |  | % of Total | 12.5% | 14.7% | 27.1% |
|  | No | Count | 225 | 301 | 526 |
|  |  | % within Sex | 71.4% | 74.0% | 72.9% |
|  |  | % of Total | 31.2% | 41.7% | 72.9% |
| Total | | Count | 315 | 407 | 722 |
|  |  | % within Sex | 100.0% | 100.0% | 100.0% |
|  |  | % of Total | 43.6% | 56.4% | 100.0% |

| **Chi-Square Tests** | | | | | |
| --- | --- | --- | --- | --- | --- |
|  | Value | df | Asymptotic Significance (2-sided) | Exact Sig. (2-sided) | Exact Sig. (1-sided) |
| Pearson Chi-Square | .573^a^ | 1 | .449 |  |  |
| Continuity Correction^b^ | .453 | 1 | .501 |  |  |
| Likelihood Ratio | .572 | 1 | .449 |  |  |
| Fisher's Exact Test |  |  |  | .449 | .250 |
| Linear-by-Linear Association | .573 | 1 | .449 |  |  |
| N of Valid Cases | 722 |  |  |  |  |
| a. 0 cells (0.0%) have expected count less than 5. The minimum expected count is 85.51. | | | | | |
| b. Computed only for a 2x2 table | | | | | |

**Books * Grade category**

| **Crosstab** | | | | | |
| --- | --- | --- | --- | --- | --- |
|  | | | Grade category | | Total |
|  |  |  | 9-10 | 11-12 |  |
| Books | Yes | Count | 98 | 98 | 196 |
|  |  | % within Grade category | 25.3% | 29.3% | 27.1% |
|  |  | % of Total | 13.6% | 13.6% | 27.1% |
|  | No | Count | 289 | 237 | 526 |
|  |  | % within Grade category | 74.7% | 70.7% | 72.9% |
|  |  | % of Total | 40.0% | 32.8% | 72.9% |
| Total | | Count | 387 | 335 | 722 |
|  |  | % within Grade category | 100.0% | 100.0% | 100.0% |
|  |  | % of Total | 53.6% | 46.4% | 100.0% |

| **Chi-Square Tests** | | | | | |
| --- | --- | --- | --- | --- | --- |
|  | Value | df | Asymptotic Significance (2-sided) | Exact Sig. (2-sided) | Exact Sig. (1-sided) |
| Pearson Chi-Square | 1.403^a^ | 1 | .236 |  |  |
| Continuity Correction^b^ | 1.211 | 1 | .271 |  |  |
| Likelihood Ratio | 1.401 | 1 | .237 |  |  |
| Fisher's Exact Test |  |  |  | .241 | .136 |
| Linear-by-Linear Association | 1.401 | 1 | .237 |  |  |
| N of Valid Cases | 722 |  |  |  |  |
| a. 0 cells (0.0%) have expected count less than 5. The minimum expected count is 90.94. | | | | | |
| b. Computed only for a 2x2 table | | | | | |

**Books * School type**

| **Crosstab** | | | | | |
| --- | --- | --- | --- | --- | --- |
|  | | | School type | | Total |
|  |  |  | Public | Private |  |
| Books | Yes | Count | 104 | 92 | 196 |
|  |  | % within School type | 24.8% | 30.5% | 27.1% |
|  |  | % of Total | 14.4% | 12.7% | 27.1% |
|  | No | Count | 316 | 210 | 526 |
|  |  | % within School type | 75.2% | 69.5% | 72.9% |
|  |  | % of Total | 43.8% | 29.1% | 72.9% |
| Total | | Count | 420 | 302 | 722 |
|  |  | % within School type | 100.0% | 100.0% | 100.0% |
|  |  | % of Total | 58.2% | 41.8% | 100.0% |

| **Chi-Square Tests** | | | | | |
| --- | --- | --- | --- | --- | --- |
|  | Value | df | Asymptotic Significance (2-sided) | Exact Sig. (2-sided) | Exact Sig. (1-sided) |
| Pearson Chi-Square | 2.888^a^ | 1 | .089 |  |  |
| Continuity Correction^b^ | 2.607 | 1 | .106 |  |  |
| Likelihood Ratio | 2.871 | 1 | .090 |  |  |
| Fisher's Exact Test |  |  |  | .091 | .054 |
| Linear-by-Linear Association | 2.884 | 1 | .089 |  |  |
| N of Valid Cases | 722 |  |  |  |  |
| a. 0 cells (0.0%) have expected count less than 5. The minimum expected count is 81.98. | | | | | |
| b. Computed only for a 2x2 table | | | | | |

**Health club * Sex**

| **Crosstab** | | | | | |
| --- | --- | --- | --- | --- | --- |
|  | | | Sex | | Total |
|  |  |  | Male | Female |  |
| Health club | Yes | Count | 35 | 57 | 92 |
|  |  | % within Sex | 11.1% | 14.0% | 12.8% |
|  |  | % of Total | 4.9% | 7.9% | 12.8% |
|  | No | Count | 279 | 350 | 629 |
|  |  | % within Sex | 88.9% | 86.0% | 87.2% |
|  |  | % of Total | 38.7% | 48.5% | 87.2% |
| Total | | Count | 314 | 407 | 721 |
|  |  | % within Sex | 100.0% | 100.0% | 100.0% |
|  |  | % of Total | 43.6% | 56.4% | 100.0% |

| **Chi-Square Tests** | | | | | |
| --- | --- | --- | --- | --- | --- |
|  | Value | df | Asymptotic Significance (2-sided) | Exact Sig. (2-sided) | Exact Sig. (1-sided) |
| Pearson Chi-Square | 1.301^a^ | 1 | .254 |  |  |
| Continuity Correction^b^ | 1.057 | 1 | .304 |  |  |
| Likelihood Ratio | 1.314 | 1 | .252 |  |  |
| Fisher's Exact Test |  |  |  | .263 | .152 |
| Linear-by-Linear Association | 1.299 | 1 | .254 |  |  |
| N of Valid Cases | 721 |  |  |  |  |
| a. 0 cells (0.0%) have expected count less than 5. The minimum expected count is 40.07. | | | | | |
| b. Computed only for a 2x2 table | | | | | |

**Health club * Grade category**

| **Crosstab** | | | | | |
| --- | --- | --- | --- | --- | --- |
|  | | | Grade category | | Total |
|  |  |  | 9-10 | 11-12 |  |
| Health club | Yes | Count | 59 | 33 | 92 |
|  |  | % within Grade category | 15.2% | 9.9% | 12.8% |
|  |  | % of Total | 8.2% | 4.6% | 12.8% |
|  | No | Count | 328 | 301 | 629 |
|  |  | % within Grade category | 84.8% | 90.1% | 87.2% |
|  |  | % of Total | 45.5% | 41.7% | 87.2% |
| Total | | Count | 387 | 334 | 721 |
|  |  | % within Grade category | 100.0% | 100.0% | 100.0% |
|  |  | % of Total | 53.7% | 46.3% | 100.0% |

| **Chi-Square Tests** | | | | | |
| --- | --- | --- | --- | --- | --- |
|  | Value | df | Asymptotic Significance (2-sided) | Exact Sig. (2-sided) | Exact Sig. (1-sided) |
| Pearson Chi-Square | 4.636^a^ | 1 | .031 |  |  |
| Continuity Correction^b^ | 4.166 | 1 | .041 |  |  |
| Likelihood Ratio | 4.709 | 1 | .030 |  |  |
| Fisher's Exact Test |  |  |  | .034 | .020 |
| Linear-by-Linear Association | 4.629 | 1 | .031 |  |  |
| N of Valid Cases | 721 |  |  |  |  |
| a. 0 cells (0.0%) have expected count less than 5. The minimum expected count is 42.62. | | | | | |
| b. Computed only for a 2x2 table | | | | | |

**Health club * School type**

| **Crosstab** | | | | | |
| --- | --- | --- | --- | --- | --- |
|  | | | School type | | Total |
|  |  |  | Public | Private |  |
| Health club | Yes | Count | 31 | 61 | 92 |
|  |  | % within School type | 7.4% | 20.2% | 12.8% |
|  |  | % of Total | 4.3% | 8.5% | 12.8% |
|  | No | Count | 388 | 241 | 629 |
|  |  | % within School type | 92.6% | 79.8% | 87.2% |
|  |  | % of Total | 53.8% | 33.4% | 87.2% |
| Total | | Count | 419 | 302 | 721 |
|  |  | % within School type | 100.0% | 100.0% | 100.0% |
|  |  | % of Total | 58.1% | 41.9% | 100.0% |

| **Chi-Square Tests** | | | | | |
| --- | --- | --- | --- | --- | --- |
|  | Value | df | Asymptotic Significance (2-sided) | Exact Sig. (2-sided) | Exact Sig. (1-sided) |
| Pearson Chi-Square | 25.831^a^ | 1 | .000 |  |  |
| Continuity Correction^b^ | 24.694 | 1 | .000 |  |  |
| Likelihood Ratio | 25.568 | 1 | .000 |  |  |
| Fisher's Exact Test |  |  |  | .000 | .000 |
| Linear-by-Linear Association | 25.795 | 1 | .000 |  |  |
| N of Valid Cases | 721 |  |  |  |  |
| a. 0 cells (0.0%) have expected count less than 5. The minimum expected count is 38.54. | | | | | |
| b. Computed only for a 2x2 table | | | | | |

**Religious bodies * Sex**

| **Crosstab** | | | | | |
| --- | --- | --- | --- | --- | --- |
|  | | | Sex | | Total |
|  |  |  | Male | Female |  |
| Religious leaders | Yes | Count | 83 | 118 | 201 |
|  |  | % within Sex | 26.3% | 29.0% | 27.8% |
|  |  | % of Total | 11.5% | 16.3% | 27.8% |
|  | No | Count | 232 | 289 | 521 |
|  |  | % within Sex | 73.7% | 71.0% | 72.2% |
|  |  | % of Total | 32.1% | 40.0% | 72.2% |
| Total | | Count | 315 | 407 | 722 |
|  |  | % within Sex | 100.0% | 100.0% | 100.0% |
|  |  | % of Total | 43.6% | 56.4% | 100.0% |

| **Chi-Square Tests** | | | | | |
| --- | --- | --- | --- | --- | --- |
|  | Value | df | Asymptotic Significance (2-sided) | Exact Sig. (2-sided) | Exact Sig. (1-sided) |
| Pearson Chi-Square | .618^a^ | 1 | .432 |  |  |
| Continuity Correction^b^ | .493 | 1 | .483 |  |  |
| Likelihood Ratio | .619 | 1 | .431 |  |  |
| Fisher's Exact Test |  |  |  | .452 | .242 |
| Linear-by-Linear Association | .617 | 1 | .432 |  |  |
| N of Valid Cases | 722 |  |  |  |  |
| a. 0 cells (0.0%) have expected count less than 5. The minimum expected count is 87.69. | | | | | |
| b. Computed only for a 2x2 table | | | | | |

**Religious bodies * Grade category**

| **Crosstab** | | | | | |
| --- | --- | --- | --- | --- | --- |
|  | | | Grade category | | Total |
|  |  |  | 9-10 | 11-12 |  |
| Religious leaders | Yes | Count | 100 | 101 | 201 |
|  |  | % within Grade category | 25.8% | 30.1% | 27.8% |
|  |  | % of Total | 13.9% | 14.0% | 27.8% |
|  | No | Count | 287 | 234 | 521 |
|  |  | % within Grade category | 74.2% | 69.9% | 72.2% |
|  |  | % of Total | 39.8% | 32.4% | 72.2% |
| Total | | Count | 387 | 335 | 722 |
|  |  | % within Grade category | 100.0% | 100.0% | 100.0% |
|  |  | % of Total | 53.6% | 46.4% | 100.0% |

| **Chi-Square Tests** | | | | | |
| --- | --- | --- | --- | --- | --- |
|  | Value | df | Asymptotic Significance (2-sided) | Exact Sig. (2-sided) | Exact Sig. (1-sided) |
| Pearson Chi-Square | 1.660^a^ | 1 | .198 |  |  |
| Continuity Correction^b^ | 1.452 | 1 | .228 |  |  |
| Likelihood Ratio | 1.657 | 1 | .198 |  |  |
| Fisher's Exact Test |  |  |  | .212 | .114 |
| Linear-by-Linear Association | 1.658 | 1 | .198 |  |  |
| N of Valid Cases | 722 |  |  |  |  |
| a. 0 cells (0.0%) have expected count less than 5. The minimum expected count is 93.26. | | | | | |
| b. Computed only for a 2x2 table | | | | | |

**Religious bodies * School type**

| **Crosstab** | | | | | |
| --- | --- | --- | --- | --- | --- |
|  | | | School type | | Total |
|  |  |  | Public | Private |  |
| Religious leaders | Yes | Count | 120 | 81 | 201 |
|  |  | % within School type | 28.6% | 26.8% | 27.8% |
|  |  | % of Total | 16.6% | 11.2% | 27.8% |
|  | No | Count | 300 | 221 | 521 |
|  |  | % within School type | 71.4% | 73.2% | 72.2% |
|  |  | % of Total | 41.6% | 30.6% | 72.2% |
| Total | | Count | 420 | 302 | 722 |
|  |  | % within School type | 100.0% | 100.0% | 100.0% |
|  |  | % of Total | 58.2% | 41.8% | 100.0% |

| **Chi-Square Tests** | | | | | |
| --- | --- | --- | --- | --- | --- |
|  | Value | df | Asymptotic Significance (2-sided) | Exact Sig. (2-sided) | Exact Sig. (1-sided) |
| Pearson Chi-Square | .268^a^ | 1 | .605 |  |  |
| Continuity Correction^b^ | .188 | 1 | .665 |  |  |
| Likelihood Ratio | .268 | 1 | .604 |  |  |
| Fisher's Exact Test |  |  |  | .615 | .333 |
| Linear-by-Linear Association | .268 | 1 | .605 |  |  |
| N of Valid Cases | 722 |  |  |  |  |
| a. 0 cells (0.0%) have expected count less than 5. The minimum expected count is 84.07. | | | | | |
| b. Computed only for a 2x2 table | | | | | |

**8. Seeking health information:** Have you ever intentionally searched or looked for health information?

**Have you ever looked/searched for health information intentionally? * Sex**

| **Crosstab** | | | | | |
| --- | --- | --- | --- | --- | --- |
|  | | | Sex | | Total |
|  |  |  | Male | Female |  |
| Have you ever looked/searched for health information intentionally? | Yes | Count | 218 | 312 | 530 |
|  |  | % within Sex | 69.2% | 76.7% | 73.4% |
|  |  | % of Total | 30.2% | 43.2% | 73.4% |
|  | No | Count | 97 | 95 | 192 |
|  |  | % within Sex | 30.8% | 23.3% | 26.6% |
|  |  | % of Total | 13.4% | 13.2% | 26.6% |
| Total | | Count | 315 | 407 | 722 |
|  |  | % within Sex | 100.0% | 100.0% | 100.0% |
|  |  | % of Total | 43.6% | 56.4% | 100.0% |

| **Chi-Square Tests** | | | | | |
| --- | --- | --- | --- | --- | --- |
|  | Value | df | Asymptotic Significance (2-sided) | Exact Sig. (2-sided) | Exact Sig. (1-sided) |
| Pearson Chi-Square | 5.052^a^ | 1 | .025 |  |  |
| Continuity Correction^b^ | 4.677 | 1 | .031 |  |  |
| Likelihood Ratio | 5.026 | 1 | .025 |  |  |
| Fisher's Exact Test |  |  |  | .027 | .015 |
| Linear-by-Linear Association | 5.045 | 1 | .025 |  |  |
| N of Valid Cases | 722 |  |  |  |  |
| a. 0 cells (0.0%) have expected count less than 5. The minimum expected count is 83.77. | | | | | |
| b. Computed only for a 2x2 table | | | | | |

**Have you ever looked/searched for health information intentionally? * Grade category**

| **Crosstab** | | | | | |
| --- | --- | --- | --- | --- | --- |
|  | | | Grade category | | Total |
|  |  |  | 9-10 | 11-12 |  |
| Have you ever looked/searched for health information intentionally? | Yes | Count | 275 | 255 | 530 |
|  |  | % within Grade category | 71.1% | 76.1% | 73.4% |
|  |  | % of Total | 38.1% | 35.3% | 73.4% |
|  | No | Count | 112 | 80 | 192 |
|  |  | % within Grade category | 28.9% | 23.9% | 26.6% |
|  |  | % of Total | 15.5% | 11.1% | 26.6% |
| Total | | Count | 387 | 335 | 722 |
|  |  | % within Grade category | 100.0% | 100.0% | 100.0% |
|  |  | % of Total | 53.6% | 46.4% | 100.0% |

| **Chi-Square Tests** | | | | | |
| --- | --- | --- | --- | --- | --- |
|  | Value | df | Asymptotic Significance (2-sided) | Exact Sig. (2-sided) | Exact Sig. (1-sided) |
| Pearson Chi-Square | 2.355^a^ | 1 | .125 |  |  |
| Continuity Correction^b^ | 2.103 | 1 | .147 |  |  |
| Likelihood Ratio | 2.365 | 1 | .124 |  |  |
| Fisher's Exact Test |  |  |  | .129 | .073 |
| Linear-by-Linear Association | 2.352 | 1 | .125 |  |  |
| N of Valid Cases | 722 |  |  |  |  |
| a. 0 cells (0.0%) have expected count less than 5. The minimum expected count is 89.09. | | | | | |
| b. Computed only for a 2x2 table | | | | | |

**Have you ever looked/searched for health information intentionally? * School type**

| **Crosstab** | | | | | |
| --- | --- | --- | --- | --- | --- |
|  | | | School type | | Total |
|  |  |  | Public | Private |  |
| Have you ever looked/searched for health information intentionally? | Yes | Count | 306 | 224 | 530 |
|  |  | % within School type | 72.9% | 74.2% | 73.4% |
|  |  | % of Total | 42.4% | 31.0% | 73.4% |
|  | No | Count | 114 | 78 | 192 |
|  |  | % within School type | 27.1% | 25.8% | 26.6% |
|  |  | % of Total | 15.8% | 10.8% | 26.6% |
| Total | | Count | 420 | 302 | 722 |
|  |  | % within School type | 100.0% | 100.0% | 100.0% |
|  |  | % of Total | 58.2% | 41.8% | 100.0% |

| **Chi-Square Tests** | | | | | |
| --- | --- | --- | --- | --- | --- |
|  | Value | df | Asymptotic Significance (2-sided) | Exact Sig. (2-sided) | Exact Sig. (1-sided) |
| Pearson Chi-Square | .156^a^ | 1 | .693 |  |  |
| Continuity Correction^b^ | .096 | 1 | .757 |  |  |
| Likelihood Ratio | .156 | 1 | .693 |  |  |
| Fisher's Exact Test |  |  |  | .733 | .379 |
| Linear-by-Linear Association | .155 | 1 | .693 |  |  |
| N of Valid Cases | 722 |  |  |  |  |
| a. 0 cells (0.0%) have expected count less than 5. The minimum expected count is 80.31. | | | | | |
| b. Computed only for a 2x2 table | | | | | |

**9. Reasons for searching for health information:** If you have ever intentionally searched for health information, what was/were the reason/s for searching for it?

**For treatment and management (personal illness) * Sex**

| **Crosstab** | | | | | |
| --- | --- | --- | --- | --- | --- |
|  | | | Sex | | Total |
|  |  |  | Male | Female |  |
| For treatment and management (personal illness) | Yes | Count | 82 | 123 | 205 |
|  |  | % within Sex | 37.6% | 39.5% | 38.8% |
|  |  | % of Total | 15.5% | 23.3% | 38.8% |
|  | No | Count | 136 | 188 | 324 |
|  |  | % within Sex | 62.4% | 60.5% | 61.2% |
|  |  | % of Total | 25.7% | 35.5% | 61.2% |
| Total | | Count | 218 | 311 | 529 |
|  |  | % within Sex | 100.0% | 100.0% | 100.0% |
|  |  | % of Total | 41.2% | 58.8% | 100.0% |

| **Chi-Square Tests** | | | | | |
| --- | --- | --- | --- | --- | --- |
|  | Value | df | Asymptotic Significance (2-sided) | Exact Sig. (2-sided) | Exact Sig. (1-sided) |
| Pearson Chi-Square | .202^a^ | 1 | .653 |  |  |
| Continuity Correction^b^ | .129 | 1 | .720 |  |  |
| Likelihood Ratio | .202 | 1 | .653 |  |  |
| Fisher's Exact Test |  |  |  | .717 | .360 |
| Linear-by-Linear Association | .202 | 1 | .653 |  |  |
| N of Valid Cases | 529 |  |  |  |  |
| a. 0 cells (0.0%) have expected count less than 5. The minimum expected count is 84.48. | | | | | |
| b. Computed only for a 2x2 table | | | | | |

**For treatment and management (personal illness) * Grade category**

| **Crosstab** | | | | | |
| --- | --- | --- | --- | --- | --- |
|  | | | Grade category | | Total |
|  |  |  | 9-10 | 11-12 |  |
| For treatment and management (personal illness) | Yes | Count | 105 | 100 | 205 |
|  |  | % within Grade category | 38.2% | 39.4% | 38.8% |
|  |  | % of Total | 19.8% | 18.9% | 38.8% |
|  | No | Count | 170 | 154 | 324 |
|  |  | % within Grade category | 61.8% | 60.6% | 61.2% |
|  |  | % of Total | 32.1% | 29.1% | 61.2% |
| Total | | Count | 275 | 254 | 529 |
|  |  | % within Grade category | 100.0% | 100.0% | 100.0% |
|  |  | % of Total | 52.0% | 48.0% | 100.0% |

| **Chi-Square Tests** | | | | | |
| --- | --- | --- | --- | --- | --- |
|  | Value | df | Asymptotic Significance (2-sided) | Exact Sig. (2-sided) | Exact Sig. (1-sided) |
| Pearson Chi-Square | .079^a^ | 1 | .779 |  |  |
| Continuity Correction^b^ | .036 | 1 | .849 |  |  |
| Likelihood Ratio | .079 | 1 | .779 |  |  |
| Fisher's Exact Test |  |  |  | .789 | .424 |
| Linear-by-Linear Association | .078 | 1 | .779 |  |  |
| N of Valid Cases | 529 |  |  |  |  |
| a. 0 cells (0.0%) have expected count less than 5. The minimum expected count is 98.43. | | | | | |
| b. Computed only for a 2x2 table | | | | | |

**For treatment and management (personal illness) * School type**

| **Crosstab** | | | | | |
| --- | --- | --- | --- | --- | --- |
|  | | | School type | | Total |
|  |  |  | Public | Private |  |
| For treatment and management (personal illness) | Yes | Count | 116 | 89 | 205 |
|  |  | % within School type | 38.0% | 39.7% | 38.8% |
|  |  | % of Total | 21.9% | 16.8% | 38.8% |
|  | No | Count | 189 | 135 | 324 |
|  |  | % within School type | 62.0% | 60.3% | 61.2% |
|  |  | % of Total | 35.7% | 25.5% | 61.2% |
| Total | | Count | 305 | 224 | 529 |
|  |  | % within School type | 100.0% | 100.0% | 100.0% |
|  |  | % of Total | 57.7% | 42.3% | 100.0% |

| **Chi-Square Tests** | | | | | |
| --- | --- | --- | --- | --- | --- |
|  | Value | df | Asymptotic Significance (2-sided) | Exact Sig. (2-sided) | Exact Sig. (1-sided) |
| Pearson Chi-Square | .157^a^ | 1 | .692 |  |  |
| Continuity Correction^b^ | .094 | 1 | .760 |  |  |
| Likelihood Ratio | .157 | 1 | .692 |  |  |
| Fisher's Exact Test |  |  |  | .718 | .379 |
| Linear-by-Linear Association | .157 | 1 | .692 |  |  |
| N of Valid Cases | 529 |  |  |  |  |
| a. 0 cells (0.0%) have expected count less than 5. The minimum expected count is 86.81. | | | | | |
| b. Computed only for a 2x2 table | | | | | |

**For prevention and maintenance (personal health) * Sex**

| **Crosstab** | | | | | |
| --- | --- | --- | --- | --- | --- |
|  | | | Sex | | Total |
|  |  |  | Male | Female |  |
| For prevention and maintenance (personal health) | Yes | Count | 148 | 224 | 372 |
|  |  | % within Sex | 67.9% | 71.8% | 70.2% |
|  |  | % of Total | 27.9% | 42.3% | 70.2% |
|  | No | Count | 70 | 88 | 158 |
|  |  | % within Sex | 32.1% | 28.2% | 29.8% |
|  |  | % of Total | 13.2% | 16.6% | 29.8% |
| Total | | Count | 218 | 312 | 530 |
|  |  | % within Sex | 100.0% | 100.0% | 100.0% |
|  |  | % of Total | 41.1% | 58.9% | 100.0% |

| **Chi-Square Tests** | | | | | |
| --- | --- | --- | --- | --- | --- |
|  | Value | df | Asymptotic Significance (2-sided) | Exact Sig. (2-sided) | Exact Sig. (1-sided) |
| Pearson Chi-Square | .935^a^ | 1 | .334 |  |  |
| Continuity Correction^b^ | .758 | 1 | .384 |  |  |
| Likelihood Ratio | .932 | 1 | .334 |  |  |
| Fisher's Exact Test |  |  |  | .337 | .192 |
| Linear-by-Linear Association | .933 | 1 | .334 |  |  |
| N of Valid Cases | 530 |  |  |  |  |
| a. 0 cells (0.0%) have expected count less than 5. The minimum expected count is 64.99. | | | | | |
| b. Computed only for a 2x2 table | | | | | |

**For prevention and maintenance (personal health) * Grade category**

| **Crosstab** | | | | | |
| --- | --- | --- | --- | --- | --- |
|  | | | Grade category | | Total |
|  |  |  | 9-10 | 11-12 |  |
| For prevention and maintenance (personal health) | Yes | Count | 196 | 176 | 372 |
|  |  | % within Grade category | 71.3% | 69.0% | 70.2% |
|  |  | % of Total | 37.0% | 33.2% | 70.2% |
|  | No | Count | 79 | 79 | 158 |
|  |  | % within Grade category | 28.7% | 31.0% | 29.8% |
|  |  | % of Total | 14.9% | 14.9% | 29.8% |
| Total | | Count | 275 | 255 | 530 |
|  |  | % within Grade category | 100.0% | 100.0% | 100.0% |
|  |  | % of Total | 51.9% | 48.1% | 100.0% |

| **Chi-Square Tests** | | | | | |
| --- | --- | --- | --- | --- | --- |
|  | Value | df | Asymptotic Significance (2-sided) | Exact Sig. (2-sided) | Exact Sig. (1-sided) |
| Pearson Chi-Square | .321^a^ | 1 | .571 |  |  |
| Continuity Correction^b^ | .222 | 1 | .637 |  |  |
| Likelihood Ratio | .321 | 1 | .571 |  |  |
| Fisher's Exact Test |  |  |  | .635 | .319 |
| Linear-by-Linear Association | .320 | 1 | .571 |  |  |
| N of Valid Cases | 530 |  |  |  |  |
| a. 0 cells (0.0%) have expected count less than 5. The minimum expected count is 76.02. | | | | | |
| b. Computed only for a 2x2 table | | | | | |

**For prevention and maintenance (personal health) * School type**

| **Crosstab** | | | | | |
| --- | --- | --- | --- | --- | --- |
|  | | | School type | | Total |
|  |  |  | Public | Private |  |
| For prevention and maintenance (personal health) | Yes | Count | 214 | 158 | 372 |
|  |  | % within School type | 69.9% | 70.5% | 70.2% |
|  |  | % of Total | 40.4% | 29.8% | 70.2% |
|  | No | Count | 92 | 66 | 158 |
|  |  | % within School type | 30.1% | 29.5% | 29.8% |
|  |  | % of Total | 17.4% | 12.5% | 29.8% |
| Total | | Count | 306 | 224 | 530 |
|  |  | % within School type | 100.0% | 100.0% | 100.0% |
|  |  | % of Total | 57.7% | 42.3% | 100.0% |

| **Chi-Square Tests** | | | | | |
| --- | --- | --- | --- | --- | --- |
|  | Value | df | Asymptotic Significance (2-sided) | Exact Sig. (2-sided) | Exact Sig. (1-sided) |
| Pearson Chi-Square | .022^a^ | 1 | .881 |  |  |
| Continuity Correction^b^ | .003 | 1 | .957 |  |  |
| Likelihood Ratio | .022 | 1 | .881 |  |  |
| Fisher's Exact Test |  |  |  | .924 | .480 |
| Linear-by-Linear Association | .022 | 1 | .881 |  |  |
| N of Valid Cases | 530 |  |  |  |  |
| a. 0 cells (0.0%) have expected count less than 5. The minimum expected count is 66.78. | | | | | |
| b. Computed only for a 2x2 table | | | | | |

**To help my parents * Sex**

| **Crosstab** | | | | | |
| --- | --- | --- | --- | --- | --- |
|  | | | Sex | | Total |
|  |  |  | Male | Female |  |
| To help my parents | Yes | Count | 46 | 70 | 116 |
|  |  | % within Sex | 21.1% | 22.4% | 21.9% |
|  |  | % of Total | 8.7% | 13.2% | 21.9% |
|  | No | Count | 172 | 242 | 414 |
|  |  | % within Sex | 78.9% | 77.6% | 78.1% |
|  |  | % of Total | 32.5% | 45.7% | 78.1% |
| Total | | Count | 218 | 312 | 530 |
|  |  | % within Sex | 100.0% | 100.0% | 100.0% |
|  |  | % of Total | 41.1% | 58.9% | 100.0% |

| **Chi-Square Tests** | | | | | |
| --- | --- | --- | --- | --- | --- |
|  | Value | df | Asymptotic Significance (2-sided) | Exact Sig. (2-sided) | Exact Sig. (1-sided) |
| Pearson Chi-Square | .134^a^ | 1 | .715 |  |  |
| Continuity Correction^b^ | .067 | 1 | .796 |  |  |
| Likelihood Ratio | .134 | 1 | .714 |  |  |
| Fisher's Exact Test |  |  |  | .750 | .399 |
| Linear-by-Linear Association | .134 | 1 | .715 |  |  |
| N of Valid Cases | 530 |  |  |  |  |
| a. 0 cells (0.0%) have expected count less than 5. The minimum expected count is 47.71. | | | | | |
| b. Computed only for a 2x2 table | | | | | |

**To help my parents * Grade category**

| **Crosstab** | | | | | |
| --- | --- | --- | --- | --- | --- |
|  | | | Grade category | | Total |
|  |  |  | 9-10 | 11-12 |  |
| To help my parents | Yes | Count | 58 | 58 | 116 |
|  |  | % within Grade category | 21.1% | 22.7% | 21.9% |
|  |  | % of Total | 10.9% | 10.9% | 21.9% |
|  | No | Count | 217 | 197 | 414 |
|  |  | % within Grade category | 78.9% | 77.3% | 78.1% |
|  |  | % of Total | 40.9% | 37.2% | 78.1% |
| Total | | Count | 275 | 255 | 530 |
|  |  | % within Grade category | 100.0% | 100.0% | 100.0% |
|  |  | % of Total | 51.9% | 48.1% | 100.0% |

| **Chi-Square Tests** | | | | | |
| --- | --- | --- | --- | --- | --- |
|  | Value | df | Asymptotic Significance (2-sided) | Exact Sig. (2-sided) | Exact Sig. (1-sided) |
| Pearson Chi-Square | .212^a^ | 1 | .645 |  |  |
| Continuity Correction^b^ | .126 | 1 | .723 |  |  |
| Likelihood Ratio | .212 | 1 | .645 |  |  |
| Fisher's Exact Test |  |  |  | .675 | .361 |
| Linear-by-Linear Association | .211 | 1 | .646 |  |  |
| N of Valid Cases | 530 |  |  |  |  |
| a. 0 cells (0.0%) have expected count less than 5. The minimum expected count is 55.81. | | | | | |
| b. Computed only for a 2x2 table | | | | | |

**To help my parents * School type**

| **Crosstab** | | | | | |
| --- | --- | --- | --- | --- | --- |
|  | | | School type | | Total |
|  |  |  | Public | Private |  |
| To help my parents | Yes | Count | 60 | 56 | 116 |
|  |  | % within School type | 19.6% | 25.0% | 21.9% |
|  |  | % of Total | 11.3% | 10.6% | 21.9% |
|  | No | Count | 246 | 168 | 414 |
|  |  | % within School type | 80.4% | 75.0% | 78.1% |
|  |  | % of Total | 46.4% | 31.7% | 78.1% |
| Total | | Count | 306 | 224 | 530 |
|  |  | % within School type | 100.0% | 100.0% | 100.0% |
|  |  | % of Total | 57.7% | 42.3% | 100.0% |

| **Chi-Square Tests** | | | | | |
| --- | --- | --- | --- | --- | --- |
|  | Value | df | Asymptotic Significance (2-sided) | Exact Sig. (2-sided) | Exact Sig. (1-sided) |
| Pearson Chi-Square | 2.199^a^ | 1 | .138 |  |  |
| Continuity Correction^b^ | 1.895 | 1 | .169 |  |  |
| Likelihood Ratio | 2.184 | 1 | .139 |  |  |
| Fisher's Exact Test |  |  |  | .167 | .085 |
| Linear-by-Linear Association | 2.195 | 1 | .138 |  |  |
| N of Valid Cases | 530 |  |  |  |  |
| a. 0 cells (0.0%) have expected count less than 5. The minimum expected count is 49.03. | | | | | |
| b. Computed only for a 2x2 table | | | | | |

**To help my siblings * Sex**

| **Crosstab** | | | | | |
| --- | --- | --- | --- | --- | --- |
|  | | | Sex | | Total |
|  |  |  | Male | Female |  |
| To help my siblings | Yes | Count | 26 | 34 | 60 |
|  |  | % within Sex | 11.9% | 10.9% | 11.3% |
|  |  | % of Total | 4.9% | 6.4% | 11.3% |
|  | No | Count | 192 | 278 | 470 |
|  |  | % within Sex | 88.1% | 89.1% | 88.7% |
|  |  | % of Total | 36.2% | 52.5% | 88.7% |
| Total | | Count | 218 | 312 | 530 |
|  |  | % within Sex | 100.0% | 100.0% | 100.0% |
|  |  | % of Total | 41.1% | 58.9% | 100.0% |

| **Chi-Square Tests** | | | | | |
| --- | --- | --- | --- | --- | --- |
|  | Value | df | Asymptotic Significance (2-sided) | Exact Sig. (2-sided) | Exact Sig. (1-sided) |
| Pearson Chi-Square | .135^a^ | 1 | .713 |  |  |
| Continuity Correction^b^ | .052 | 1 | .819 |  |  |
| Likelihood Ratio | .135 | 1 | .713 |  |  |
| Fisher's Exact Test |  |  |  | .781 | .407 |
| Linear-by-Linear Association | .135 | 1 | .713 |  |  |
| N of Valid Cases | 530 |  |  |  |  |
| a. 0 cells (0.0%) have expected count less than 5. The minimum expected count is 24.68. | | | | | |
| b. Computed only for a 2x2 table | | | | | |

**To help my siblings * Grade category**

| **Crosstab** | | | | | |
| --- | --- | --- | --- | --- | --- |
|  | | | Grade category | | Total |
|  |  |  | 9-10 | 11-12 |  |
| To help my siblings | Yes | Count | 36 | 24 | 60 |
|  |  | % within Grade category | 13.1% | 9.4% | 11.3% |
|  |  | % of Total | 6.8% | 4.5% | 11.3% |
|  | No | Count | 239 | 231 | 470 |
|  |  | % within Grade category | 86.9% | 90.6% | 88.7% |
|  |  | % of Total | 45.1% | 43.6% | 88.7% |
| Total | | Count | 275 | 255 | 530 |
|  |  | % within Grade category | 100.0% | 100.0% | 100.0% |
|  |  | % of Total | 51.9% | 48.1% | 100.0% |

| **Chi-Square Tests** | | | | | |
| --- | --- | --- | --- | --- | --- |
|  | Value | df | Asymptotic Significance (2-sided) | Exact Sig. (2-sided) | Exact Sig. (1-sided) |
| Pearson Chi-Square | 1.784^a^ | 1 | .182 |  |  |
| Continuity Correction^b^ | 1.436 | 1 | .231 |  |  |
| Likelihood Ratio | 1.798 | 1 | .180 |  |  |
| Fisher's Exact Test |  |  |  | .217 | .115 |
| Linear-by-Linear Association | 1.781 | 1 | .182 |  |  |
| N of Valid Cases | 530 |  |  |  |  |
| a. 0 cells (0.0%) have expected count less than 5. The minimum expected count is 28.87. | | | | | |
| b. Computed only for a 2x2 table | | | | | |

**To help my siblings * School type**

| **Crosstab** | | | | | |
| --- | --- | --- | --- | --- | --- |
|  | | | School type | | Total |
|  |  |  | Public | Private |  |
| To help my siblings | Yes | Count | 38 | 22 | 60 |
|  |  | % within School type | 12.4% | 9.8% | 11.3% |
|  |  | % of Total | 7.2% | 4.2% | 11.3% |
|  | No | Count | 268 | 202 | 470 |
|  |  | % within School type | 87.6% | 90.2% | 88.7% |
|  |  | % of Total | 50.6% | 38.1% | 88.7% |
| Total | | Count | 306 | 224 | 530 |
|  |  | % within School type | 100.0% | 100.0% | 100.0% |
|  |  | % of Total | 57.7% | 42.3% | 100.0% |

| **Chi-Square Tests** | | | | | |
| --- | --- | --- | --- | --- | --- |
|  | Value | df | Asymptotic Significance (2-sided) | Exact Sig. (2-sided) | Exact Sig. (1-sided) |
| Pearson Chi-Square | .869^a^ | 1 | .351 |  |  |
| Continuity Correction^b^ | .629 | 1 | .428 |  |  |
| Likelihood Ratio | .880 | 1 | .348 |  |  |
| Fisher's Exact Test |  |  |  | .406 | .215 |
| Linear-by-Linear Association | .867 | 1 | .352 |  |  |
| N of Valid Cases | 530 |  |  |  |  |
| a. 0 cells (0.0%) have expected count less than 5. The minimum expected count is 25.36. | | | | | |
| b. Computed only for a 2x2 table | | | | | |

**To help my relatives * Sex**

| **Crosstab** | | | | | |
| --- | --- | --- | --- | --- | --- |
|  | | | Sex | | Total |
|  |  |  | Male | Female |  |
| To help my relatives | Yes | Count | 21 | 24 | 45 |
|  |  | % within Sex | 9.6% | 7.7% | 8.5% |
|  |  | % of Total | 4.0% | 4.5% | 8.5% |
|  | No | Count | 197 | 288 | 485 |
|  |  | % within Sex | 90.4% | 92.3% | 91.5% |
|  |  | % of Total | 37.2% | 54.3% | 91.5% |
| Total | | Count | 218 | 312 | 530 |
|  |  | % within Sex | 100.0% | 100.0% | 100.0% |
|  |  | % of Total | 41.1% | 58.9% | 100.0% |

| **Chi-Square Tests** | | | | | |
| --- | --- | --- | --- | --- | --- |
|  | Value | df | Asymptotic Significance (2-sided) | Exact Sig. (2-sided) | Exact Sig. (1-sided) |
| Pearson Chi-Square | .622^a^ | 1 | .430 |  |  |
| Continuity Correction^b^ | .397 | 1 | .528 |  |  |
| Likelihood Ratio | .616 | 1 | .433 |  |  |
| Fisher's Exact Test |  |  |  | .433 | .263 |
| Linear-by-Linear Association | .621 | 1 | .431 |  |  |
| N of Valid Cases | 530 |  |  |  |  |
| a. 0 cells (0.0%) have expected count less than 5. The minimum expected count is 18.51. | | | | | |
| b. Computed only for a 2x2 table | | | | | |

**To help my relatives * Grade category**

| **Crosstab** | | | | | |
| --- | --- | --- | --- | --- | --- |
|  | | | Grade category | | Total |
|  |  |  | 9-10 | 11-12 |  |
| To help my relatives | Yes | Count | 27 | 18 | 45 |
|  |  | % within Grade category | 9.8% | 7.1% | 8.5% |
|  |  | % of Total | 5.1% | 3.4% | 8.5% |
|  | No | Count | 248 | 237 | 485 |
|  |  | % within Grade category | 90.2% | 92.9% | 91.5% |
|  |  | % of Total | 46.8% | 44.7% | 91.5% |
| Total | | Count | 275 | 255 | 530 |
|  |  | % within Grade category | 100.0% | 100.0% | 100.0% |
|  |  | % of Total | 51.9% | 48.1% | 100.0% |

| **Chi-Square Tests** | | | | | |
| --- | --- | --- | --- | --- | --- |
|  | Value | df | Asymptotic Significance (2-sided) | Exact Sig. (2-sided) | Exact Sig. (1-sided) |
| Pearson Chi-Square | 1.297^a^ | 1 | .255 |  |  |
| Continuity Correction^b^ | .966 | 1 | .326 |  |  |
| Likelihood Ratio | 1.307 | 1 | .253 |  |  |
| Fisher's Exact Test |  |  |  | .278 | .163 |
| Linear-by-Linear Association | 1.294 | 1 | .255 |  |  |
| N of Valid Cases | 530 |  |  |  |  |
| a. 0 cells (0.0%) have expected count less than 5. The minimum expected count is 21.65. | | | | | |
| b. Computed only for a 2x2 table | | | | | |

**To help my relatives * School type**

| **Crosstab** | | | | | |
| --- | --- | --- | --- | --- | --- |
|  | | | School type | | Total |
|  |  |  | Public | Private |  |
| To help my relatives | Yes | Count | 25 | 20 | 45 |
|  |  | % within School type | 8.2% | 8.9% | 8.5% |
|  |  | % of Total | 4.7% | 3.8% | 8.5% |
|  | No | Count | 281 | 204 | 485 |
|  |  | % within School type | 91.8% | 91.1% | 91.5% |
|  |  | % of Total | 53.0% | 38.5% | 91.5% |
| Total | | Count | 306 | 224 | 530 |
|  |  | % within School type | 100.0% | 100.0% | 100.0% |
|  |  | % of Total | 57.7% | 42.3% | 100.0% |

| **Chi-Square Tests** | | | | | |
| --- | --- | --- | --- | --- | --- |
|  | Value | df | Asymptotic Significance (2-sided) | Exact Sig. (2-sided) | Exact Sig. (1-sided) |
| Pearson Chi-Square | .096^a^ | 1 | .757 |  |  |
| Continuity Correction^b^ | .023 | 1 | .879 |  |  |
| Likelihood Ratio | .095 | 1 | .757 |  |  |
| Fisher's Exact Test |  |  |  | .755 | .437 |
| Linear-by-Linear Association | .096 | 1 | .757 |  |  |
| N of Valid Cases | 530 |  |  |  |  |
| a. 0 cells (0.0%) have expected count less than 5. The minimum expected count is 19.02. | | | | | |
| b. Computed only for a 2x2 table | | | | | |

**To learn about health issues * Sex**

| **Crosstab** | | | | | |
| --- | --- | --- | --- | --- | --- |
|  | | | Sex | | Total |
|  |  |  | Male | Female |  |
| To learn about health issues | Yes | Count | 84 | 122 | 206 |
|  |  | % within Sex | 38.5% | 39.1% | 38.9% |
|  |  | % of Total | 15.8% | 23.0% | 38.9% |
|  | No | Count | 134 | 190 | 324 |
|  |  | % within Sex | 61.5% | 60.9% | 61.1% |
|  |  | % of Total | 25.3% | 35.8% | 61.1% |
| Total | | Count | 218 | 312 | 530 |
|  |  | % within Sex | 100.0% | 100.0% | 100.0% |
|  |  | % of Total | 41.1% | 58.9% | 100.0% |

| **Chi-Square Tests** | | | | | |
| --- | --- | --- | --- | --- | --- |
|  | Value | df | Asymptotic Significance (2-sided) | Exact Sig. (2-sided) | Exact Sig. (1-sided) |
| Pearson Chi-Square | .018^a^ | 1 | .895 |  |  |
| Continuity Correction^b^ | .002 | 1 | .966 |  |  |
| Likelihood Ratio | .018 | 1 | .895 |  |  |
| Fisher's Exact Test |  |  |  | .928 | .484 |
| Linear-by-Linear Association | .018 | 1 | .895 |  |  |
| N of Valid Cases | 530 |  |  |  |  |
| a. 0 cells (0.0%) have expected count less than 5. The minimum expected count is 84.73. | | | | | |
| b. Computed only for a 2x2 table | | | | | |

**To learn about health issues * Grade category**

| **Crosstab** | | | | | |
| --- | --- | --- | --- | --- | --- |
|  | | | Grade category | | Total |
|  |  |  | 9-10 | 11-12 |  |
| To learn about health issues | Yes | Count | 103 | 103 | 206 |
|  |  | % within Grade category | 37.5% | 40.4% | 38.9% |
|  |  | % of Total | 19.4% | 19.4% | 38.9% |
|  | No | Count | 172 | 152 | 324 |
|  |  | % within Grade category | 62.5% | 59.6% | 61.1% |
|  |  | % of Total | 32.5% | 28.7% | 61.1% |
| Total | | Count | 275 | 255 | 530 |
|  |  | % within Grade category | 100.0% | 100.0% | 100.0% |
|  |  | % of Total | 51.9% | 48.1% | 100.0% |

| **Chi-Square Tests** | | | | | |
| --- | --- | --- | --- | --- | --- |
|  | Value | df | Asymptotic Significance (2-sided) | Exact Sig. (2-sided) | Exact Sig. (1-sided) |
| Pearson Chi-Square | .481^a^ | 1 | .488 |  |  |
| Continuity Correction^b^ | .365 | 1 | .546 |  |  |
| Likelihood Ratio | .480 | 1 | .488 |  |  |
| Fisher's Exact Test |  |  |  | .533 | .273 |
| Linear-by-Linear Association | .480 | 1 | .489 |  |  |
| N of Valid Cases | 530 |  |  |  |  |
| a. 0 cells (0.0%) have expected count less than 5. The minimum expected count is 99.11. | | | | | |
| b. Computed only for a 2x2 table | | | | | |

**To learn about health issues * School type**

| **Crosstab** | | | | | |
| --- | --- | --- | --- | --- | --- |
|  | | | School type | | Total |
|  |  |  | Public | Private |  |
| To learn about health issues | Yes | Count | 106 | 100 | 206 |
|  |  | % within School type | 34.6% | 44.6% | 38.9% |
|  |  | % of Total | 20.0% | 18.9% | 38.9% |
|  | No | Count | 200 | 124 | 324 |
|  |  | % within School type | 65.4% | 55.4% | 61.1% |
|  |  | % of Total | 37.7% | 23.4% | 61.1% |
| Total | | Count | 306 | 224 | 530 |
|  |  | % within School type | 100.0% | 100.0% | 100.0% |
|  |  | % of Total | 57.7% | 42.3% | 100.0% |

| **Chi-Square Tests** | | | | | |
| --- | --- | --- | --- | --- | --- |
|  | Value | df | Asymptotic Significance (2-sided) | Exact Sig. (2-sided) | Exact Sig. (1-sided) |
| Pearson Chi-Square | 5.445^a^ | 1 | .020 |  |  |
| Continuity Correction^b^ | 5.033 | 1 | .025 |  |  |
| Likelihood Ratio | 5.431 | 1 | .020 |  |  |
| Fisher's Exact Test |  |  |  | .024 | .013 |
| Linear-by-Linear Association | 5.435 | 1 | .020 |  |  |
| N of Valid Cases | 530 |  |  |  |  |
| a. 0 cells (0.0%) have expected count less than 5. The minimum expected count is 87.06. | | | | | |
| b. Computed only for a 2x2 table | | | | | |

**10. Specific type of health information sought:** If you have ever intentionally searched for health information, what specific health topics did you search for?

**Mental health related such as stress or depression * Sex**

| **Crosstab** | | | | | |
| --- | --- | --- | --- | --- | --- |
|  | | | Sex | | Total |
|  |  |  | Male | Female |  |
| Mental health related such as stress or depression | Yes | Count | 87 | 139 | 226 |
|  |  | % within Sex | 40.3% | 44.7% | 42.9% |
|  |  | % of Total | 16.5% | 26.4% | 42.9% |
|  | No | Count | 129 | 172 | 301 |
|  |  | % within Sex | 59.7% | 55.3% | 57.1% |
|  |  | % of Total | 24.5% | 32.6% | 57.1% |
| Total | | Count | 216 | 311 | 527 |
|  |  | % within Sex | 100.0% | 100.0% | 100.0% |
|  |  | % of Total | 41.0% | 59.0% | 100.0% |

| **Chi-Square Tests** | | | | | |
| --- | --- | --- | --- | --- | --- |
|  | Value | df | Asymptotic Significance (2-sided) | Exact Sig. (2-sided) | Exact Sig. (1-sided) |
| Pearson Chi-Square | 1.015^a^ | 1 | .314 |  |  |
| Continuity Correction^b^ | .843 | 1 | .359 |  |  |
| Likelihood Ratio | 1.017 | 1 | .313 |  |  |
| Fisher's Exact Test |  |  |  | .326 | .179 |
| Linear-by-Linear Association | 1.013 | 1 | .314 |  |  |
| N of Valid Cases | 527 |  |  |  |  |
| a. 0 cells (0.0%) have expected count less than 5. The minimum expected count is 92.63. | | | | | |
| b. Computed only for a 2x2 table | | | | | |

**Mental health related such as stress or depression * Grade category**

| **Crosstab** | | | | | |
| --- | --- | --- | --- | --- | --- |
|  | | | Grade category | | Total |
|  |  |  | 9-10 | 11-12 |  |
| Mental health related such as stress or depression | Yes | Count | 117 | 109 | 226 |
|  |  | % within Grade category | 42.7% | 43.1% | 42.9% |
|  |  | % of Total | 22.2% | 20.7% | 42.9% |
|  | No | Count | 157 | 144 | 301 |
|  |  | % within Grade category | 57.3% | 56.9% | 57.1% |
|  |  | % of Total | 29.8% | 27.3% | 57.1% |
| Total | | Count | 274 | 253 | 527 |
|  |  | % within Grade category | 100.0% | 100.0% | 100.0% |
|  |  | % of Total | 52.0% | 48.0% | 100.0% |

| **Chi-Square Tests** | | | | | |
| --- | --- | --- | --- | --- | --- |
|  | Value | df | Asymptotic Significance (2-sided) | Exact Sig. (2-sided) | Exact Sig. (1-sided) |
| Pearson Chi-Square | .008^a^ | 1 | .929 |  |  |
| Continuity Correction^b^ | .000 | 1 | 1.000 |  |  |
| Likelihood Ratio | .008 | 1 | .929 |  |  |
| Fisher's Exact Test |  |  |  | .930 | .500 |
| Linear-by-Linear Association | .008 | 1 | .929 |  |  |
| N of Valid Cases | 527 |  |  |  |  |
| a. 0 cells (0.0%) have expected count less than 5. The minimum expected count is 108.50. | | | | | |
| b. Computed only for a 2x2 table | | | | | |

**Mental health related such as stress or depression * School type**

| **Crosstab** | | | | | |
| --- | --- | --- | --- | --- | --- |
|  | | | School type | | Total |
|  |  |  | Public | Private |  |
| Mental health related such as stress or depression | Yes | Count | 135 | 91 | 226 |
|  |  | % within School type | 44.6% | 40.6% | 42.9% |
|  |  | % of Total | 25.6% | 17.3% | 42.9% |
|  | No | Count | 168 | 133 | 301 |
|  |  | % within School type | 55.4% | 59.4% | 57.1% |
|  |  | % of Total | 31.9% | 25.2% | 57.1% |
| Total | | Count | 303 | 224 | 527 |
|  |  | % within School type | 100.0% | 100.0% | 100.0% |
|  |  | % of Total | 57.5% | 42.5% | 100.0% |

| **Chi-Square Tests** | | | | | |
| --- | --- | --- | --- | --- | --- |
|  | Value | df | Asymptotic Significance (2-sided) | Exact Sig. (2-sided) | Exact Sig. (1-sided) |
| Pearson Chi-Square | .812^a^ | 1 | .368 |  |  |
| Continuity Correction^b^ | .659 | 1 | .417 |  |  |
| Likelihood Ratio | .813 | 1 | .367 |  |  |
| Fisher's Exact Test |  |  |  | .375 | .208 |
| Linear-by-Linear Association | .810 | 1 | .368 |  |  |
| N of Valid Cases | 527 |  |  |  |  |
| a. 0 cells (0.0%) have expected count less than 5. The minimum expected count is 96.06. | | | | | |
| b. Computed only for a 2x2 table | | | | | |

**Sexual and reproductive health related issues * Sex**

| **Crosstab** | | | | | |
| --- | --- | --- | --- | --- | --- |
|  | | | Sex | | Total |
|  |  |  | Male | Female |  |
| Sexual and reproductive health related issues | Yes | Count | 74 | 140 | 214 |
|  |  | % within Sex | 34.3% | 45.0% | 40.6% |
|  |  | % of Total | 14.0% | 26.6% | 40.6% |
|  | No | Count | 142 | 171 | 313 |
|  |  | % within Sex | 65.7% | 55.0% | 59.4% |
|  |  | % of Total | 26.9% | 32.4% | 59.4% |
| Total | | Count | 216 | 311 | 527 |
|  |  | % within Sex | 100.0% | 100.0% | 100.0% |
|  |  | % of Total | 41.0% | 59.0% | 100.0% |

| **Chi-Square Tests** | | | | | |
| --- | --- | --- | --- | --- | --- |
|  | Value | df | Asymptotic Significance (2-sided) | Exact Sig. (2-sided) | Exact Sig. (1-sided) |
| Pearson Chi-Square | 6.116^a^ | 1 | .013 |  |  |
| Continuity Correction^b^ | 5.678 | 1 | .017 |  |  |
| Likelihood Ratio | 6.162 | 1 | .013 |  |  |
| Fisher's Exact Test |  |  |  | .015 | .008 |
| Linear-by-Linear Association | 6.104 | 1 | .013 |  |  |
| N of Valid Cases | 527 |  |  |  |  |
| a. 0 cells (0.0%) have expected count less than 5. The minimum expected count is 87.71. | | | | | |
| b. Computed only for a 2x2 table | | | | | |

**Sexual and reproductive health related issues * Grade category**

| **Crosstab** | | | | | |
| --- | --- | --- | --- | --- | --- |
|  | | | Grade category | | Total |
|  |  |  | 9-10 | 11-12 |  |
| Sexual and reproductive health related issues | Yes | Count | 114 | 100 | 214 |
|  |  | % within Grade category | 41.6% | 39.5% | 40.6% |
|  |  | % of Total | 21.6% | 19.0% | 40.6% |
|  | No | Count | 160 | 153 | 313 |
|  |  | % within Grade category | 58.4% | 60.5% | 59.4% |
|  |  | % of Total | 30.4% | 29.0% | 59.4% |
| Total | | Count | 274 | 253 | 527 |
|  |  | % within Grade category | 100.0% | 100.0% | 100.0% |
|  |  | % of Total | 52.0% | 48.0% | 100.0% |

| **Chi-Square Tests** | | | | | |
| --- | --- | --- | --- | --- | --- |
|  | Value | df | Asymptotic Significance (2-sided) | Exact Sig. (2-sided) | Exact Sig. (1-sided) |
| Pearson Chi-Square | .236^a^ | 1 | .627 |  |  |
| Continuity Correction^b^ | .158 | 1 | .691 |  |  |
| Likelihood Ratio | .236 | 1 | .627 |  |  |
| Fisher's Exact Test |  |  |  | .658 | .346 |
| Linear-by-Linear Association | .236 | 1 | .627 |  |  |
| N of Valid Cases | 527 |  |  |  |  |
| a. 0 cells (0.0%) have expected count less than 5. The minimum expected count is 102.74. | | | | | |
| b. Computed only for a 2x2 table | | | | | |

**Sexual and reproductive health related issues * School type**

| **Crosstab** | | | | | |
| --- | --- | --- | --- | --- | --- |
|  | | | School type | | Total |
|  |  |  | Public | Private |  |
| Sexual and reproductive health related issues | Yes | Count | 138 | 76 | 214 |
|  |  | % within School type | 45.5% | 33.9% | 40.6% |
|  |  | % of Total | 26.2% | 14.4% | 40.6% |
|  | No | Count | 165 | 148 | 313 |
|  |  | % within School type | 54.5% | 66.1% | 59.4% |
|  |  | % of Total | 31.3% | 28.1% | 59.4% |
| Total | | Count | 303 | 224 | 527 |
|  |  | % within School type | 100.0% | 100.0% | 100.0% |
|  |  | % of Total | 57.5% | 42.5% | 100.0% |

| **Chi-Square Tests** | | | | | |
| --- | --- | --- | --- | --- | --- |
|  | Value | df | Asymptotic Significance (2-sided) | Exact Sig. (2-sided) | Exact Sig. (1-sided) |
| Pearson Chi-Square | 7.205^a^ | 1 | .007 |  |  |
| Continuity Correction^b^ | 6.732 | 1 | .009 |  |  |
| Likelihood Ratio | 7.259 | 1 | .007 |  |  |
| Fisher's Exact Test |  |  |  | .009 | .005 |
| Linear-by-Linear Association | 7.192 | 1 | .007 |  |  |
| N of Valid Cases | 527 |  |  |  |  |
| a. 0 cells (0.0%) have expected count less than 5. The minimum expected count is 90.96. | | | | | |
| b. Computed only for a 2x2 table | | | | | |

**Chronic conditions related issues (e.g., diabetes, blood pressure, cancer) * Sex**

| **Crosstab** | | | | | |
| --- | --- | --- | --- | --- | --- |
|  | | | Sex | | Total |
|  |  |  | Male | Female |  |
| Chronic conditions related issues (e.g.,diabetes, blood pressure, cancer) | Yes | Count | 33 | 56 | 89 |
|  |  | % within Sex | 15.2% | 18.0% | 16.9% |
|  |  | % of Total | 6.3% | 10.6% | 16.9% |
|  | No | Count | 184 | 255 | 439 |
|  |  | % within Sex | 84.8% | 82.0% | 83.1% |
|  |  | % of Total | 34.8% | 48.3% | 83.1% |
| Total | | Count | 217 | 311 | 528 |
|  |  | % within Sex | 100.0% | 100.0% | 100.0% |
|  |  | % of Total | 41.1% | 58.9% | 100.0% |

| **Chi-Square Tests** | | | | | |
| --- | --- | --- | --- | --- | --- |
|  | Value | df | Asymptotic Significance (2-sided) | Exact Sig. (2-sided) | Exact Sig. (1-sided) |
| Pearson Chi-Square | .715^a^ | 1 | .398 |  |  |
| Continuity Correction^b^ | .529 | 1 | .467 |  |  |
| Likelihood Ratio | .721 | 1 | .396 |  |  |
| Fisher's Exact Test |  |  |  | .411 | .234 |
| Linear-by-Linear Association | .713 | 1 | .398 |  |  |
| N of Valid Cases | 528 |  |  |  |  |
| a. 0 cells (0.0%) have expected count less than 5. The minimum expected count is 36.58. | | | | | |
| b. Computed only for a 2x2 table | | | | | |

**Chronic conditions related issues (e.g., diabetes, blood pressure, cancer) * Grade category**

| **Crosstab** | | | | | |
| --- | --- | --- | --- | --- | --- |
|  | | | Grade category | | Total |
|  |  |  | 9-10 | 11-12 |  |
| Chronic conditions related issues (e.g.,diabetes, blood pressure, cancer) | Yes | Count | 53 | 36 | 89 |
|  |  | % within Grade category | 19.3% | 14.2% | 16.9% |
|  |  | % of Total | 10.0% | 6.8% | 16.9% |
|  | No | Count | 221 | 218 | 439 |
|  |  | % within Grade category | 80.7% | 85.8% | 83.1% |
|  |  | % of Total | 41.9% | 41.3% | 83.1% |
| Total | | Count | 274 | 254 | 528 |
|  |  | % within Grade category | 100.0% | 100.0% | 100.0% |
|  |  | % of Total | 51.9% | 48.1% | 100.0% |

| **Chi-Square Tests** | | | | | |
| --- | --- | --- | --- | --- | --- |
|  | Value | df | Asymptotic Significance (2-sided) | Exact Sig. (2-sided) | Exact Sig. (1-sided) |
| Pearson Chi-Square | 2.514^a^ | 1 | .113 |  |  |
| Continuity Correction^b^ | 2.158 | 1 | .142 |  |  |
| Likelihood Ratio | 2.530 | 1 | .112 |  |  |
| Fisher's Exact Test |  |  |  | .131 | .071 |
| Linear-by-Linear Association | 2.509 | 1 | .113 |  |  |
| N of Valid Cases | 528 |  |  |  |  |
| a. 0 cells (0.0%) have expected count less than 5. The minimum expected count is 42.81. | | | | | |
| b. Computed only for a 2x2 table | | | | | |

**Chronic conditions related issues (e.g., diabetes, blood pressure, cancer) * School type**

| **Crosstab** | | | | | |
| --- | --- | --- | --- | --- | --- |
|  | | | School type | | Total |
|  |  |  | Public | Private |  |
| Chronic conditions related issues (e.g., diabetes, blood pressure, cancer) | Yes | Count | 51 | 38 | 89 |
|  |  | % within School type | 16.8% | 17.0% | 16.9% |
|  |  | % of Total | 9.7% | 7.2% | 16.9% |
|  | No | Count | 253 | 186 | 439 |
|  |  | % within School type | 83.2% | 83.0% | 83.1% |
|  |  | % of Total | 47.9% | 35.2% | 83.1% |
| Total | | Count | 304 | 224 | 528 |
|  |  | % within School type | 100.0% | 100.0% | 100.0% |
|  |  | % of Total | 57.6% | 42.4% | 100.0% |

| **Chi-Square Tests** | | | | | |
| --- | --- | --- | --- | --- | --- |
|  | Value | df | Asymptotic Significance (2-sided) | Exact Sig. (2-sided) | Exact Sig. (1-sided) |
| Pearson Chi-Square | .003^a^ | 1 | .955 |  |  |
| Continuity Correction^b^ | .000 | 1 | 1.000 |  |  |
| Likelihood Ratio | .003 | 1 | .955 |  |  |
| Fisher's Exact Test |  |  |  | 1.000 | .523 |
| Linear-by-Linear Association | .003 | 1 | .955 |  |  |
| N of Valid Cases | 528 |  |  |  |  |
| a. 0 cells (0.0%) have expected count less than 5. The minimum expected count is 37.76. | | | | | |
| b. Computed only for a 2x2 table | | | | | |

**11. Usually used sources of information to get the required information:** Whenever you look for health information, whom do you usually ask or from where do you usually get it?

**Parents/guardians * Sex**

| **Crosstab** | | | | | |
| --- | --- | --- | --- | --- | --- |
|  | | | Sex | | Total |
|  |  |  | Male | Female |  |
| Parents/guardians | Yes | Count | 175 | 264 | 439 |
|  |  | % within Sex | 55.6% | 64.9% | 60.8% |
|  |  | % of Total | 24.2% | 36.6% | 60.8% |
|  | No | Count | 140 | 143 | 283 |
|  |  | % within Sex | 44.4% | 35.1% | 39.2% |
|  |  | % of Total | 19.4% | 19.8% | 39.2% |
| Total | | Count | 315 | 407 | 722 |
|  |  | % within Sex | 100.0% | 100.0% | 100.0% |
|  |  | % of Total | 43.6% | 56.4% | 100.0% |

| **Chi-Square Tests** | | | | | |
| --- | --- | --- | --- | --- | --- |
|  | Value | df | Asymptotic Significance (2-sided) | Exact Sig. (2-sided) | Exact Sig. (1-sided) |
| Pearson Chi-Square | 6.457^a^ | 1 | .011 |  |  |
| Continuity Correction^b^ | 6.072 | 1 | .014 |  |  |
| Likelihood Ratio | 6.446 | 1 | .011 |  |  |
| Fisher's Exact Test |  |  |  | .011 | .007 |
| Linear-by-Linear Association | 6.448 | 1 | .011 |  |  |
| N of Valid Cases | 722 |  |  |  |  |
| a. 0 cells (0.0%) have expected count less than 5. The minimum expected count is 123.47. | | | | | |
| b. Computed only for a 2x2 table | | | | | |

**Parents/guardians * Grade category**

| **Crosstab** | | | | | |
| --- | --- | --- | --- | --- | --- |
|  | | | Grade category | | Total |
|  |  |  | 9-10 | 11-12 |  |
| Parents/guardians | Yes | Count | 247 | 192 | 439 |
|  |  | % within Grade category | 63.8% | 57.3% | 60.8% |
|  |  | % of Total | 34.2% | 26.6% | 60.8% |
|  | No | Count | 140 | 143 | 283 |
|  |  | % within Grade category | 36.2% | 42.7% | 39.2% |
|  |  | % of Total | 19.4% | 19.8% | 39.2% |
| Total | | Count | 387 | 335 | 722 |
|  |  | % within Grade category | 100.0% | 100.0% | 100.0% |
|  |  | % of Total | 53.6% | 46.4% | 100.0% |

| **Chi-Square Tests** | | | | | |
| --- | --- | --- | --- | --- | --- |
|  | Value | df | Asymptotic Significance (2-sided) | Exact Sig. (2-sided) | Exact Sig. (1-sided) |
| Pearson Chi-Square | 3.194^a^ | 1 | .074 |  |  |
| Continuity Correction^b^ | 2.927 | 1 | .087 |  |  |
| Likelihood Ratio | 3.192 | 1 | .074 |  |  |
| Fisher's Exact Test |  |  |  | .079 | .044 |
| Linear-by-Linear Association | 3.189 | 1 | .074 |  |  |
| N of Valid Cases | 722 |  |  |  |  |
| a. 0 cells (0.0%) have expected count less than 5. The minimum expected count is 131.31. | | | | | |
| b. Computed only for a 2x2 table | | | | | |

**Parents/guardians * School type**

| **Crosstab** | | | | | |
| --- | --- | --- | --- | --- | --- |
|  | | | School type | | Total |
|  |  |  | Public | Private |  |
| Parents/guardians | Yes | Count | 242 | 197 | 439 |
|  |  | % within School type | 57.6% | 65.2% | 60.8% |
|  |  | % of Total | 33.5% | 27.3% | 60.8% |
|  | No | Count | 178 | 105 | 283 |
|  |  | % within School type | 42.4% | 34.8% | 39.2% |
|  |  | % of Total | 24.7% | 14.5% | 39.2% |
| Total | | Count | 420 | 302 | 722 |
|  |  | % within School type | 100.0% | 100.0% | 100.0% |
|  |  | % of Total | 58.2% | 41.8% | 100.0% |

| **Chi-Square Tests** | | | | | |
| --- | --- | --- | --- | --- | --- |
|  | Value | df | Asymptotic Significance (2-sided) | Exact Sig. (2-sided) | Exact Sig. (1-sided) |
| Pearson Chi-Square | 4.272^a^ | 1 | .039 |  |  |
| Continuity Correction^b^ | 3.958 | 1 | .047 |  |  |
| Likelihood Ratio | 4.294 | 1 | .038 |  |  |
| Fisher's Exact Test |  |  |  | .044 | .023 |
| Linear-by-Linear Association | 4.266 | 1 | .039 |  |  |
| N of Valid Cases | 722 |  |  |  |  |
| a. 0 cells (0.0%) have expected count less than 5. The minimum expected count is 118.37. | | | | | |
| b. Computed only for a 2x2 table | | | | | |

**Siblings (brother/sister) * Sex**

| **Crosstab** | | | | | |
| --- | --- | --- | --- | --- | --- |
|  | | | Sex | | Total |
|  |  |  | Male | Female |  |
| Siblings (brother/sister) | Yes | Count | 30 | 50 | 80 |
|  |  | % within Sex | 9.5% | 12.3% | 11.1% |
|  |  | % of Total | 4.2% | 6.9% | 11.1% |
|  | No | Count | 285 | 357 | 642 |
|  |  | % within Sex | 90.5% | 87.7% | 88.9% |
|  |  | % of Total | 39.5% | 49.4% | 88.9% |
| Total | | Count | 315 | 407 | 722 |
|  |  | % within Sex | 100.0% | 100.0% | 100.0% |
|  |  | % of Total | 43.6% | 56.4% | 100.0% |

| **Chi-Square Tests** | | | | | |
| --- | --- | --- | --- | --- | --- |
|  | Value | df | Asymptotic Significance (2-sided) | Exact Sig. (2-sided) | Exact Sig. (1-sided) |
| Pearson Chi-Square | 1.374^a^ | 1 | .241 |  |  |
| Continuity Correction^b^ | 1.108 | 1 | .292 |  |  |
| Likelihood Ratio | 1.390 | 1 | .238 |  |  |
| Fisher's Exact Test |  |  |  | .282 | .146 |
| Linear-by-Linear Association | 1.372 | 1 | .241 |  |  |
| N of Valid Cases | 722 |  |  |  |  |
| a. 0 cells (0.0%) have expected count less than 5. The minimum expected count is 34.90. | | | | | |
| b. Computed only for a 2x2 table | | | | | |

**Siblings (brother/sister) * Grade category**

| **Crosstab** | | | | | |
| --- | --- | --- | --- | --- | --- |
|  | | | Grade category | | Total |
|  |  |  | 9-10 | 11-12 |  |
| Siblings (brother/sister) | Yes | Count | 45 | 35 | 80 |
|  |  | % within Grade category | 11.6% | 10.4% | 11.1% |
|  |  | % of Total | 6.2% | 4.8% | 11.1% |
|  | No | Count | 342 | 300 | 642 |
|  |  | % within Grade category | 88.4% | 89.6% | 88.9% |
|  |  | % of Total | 47.4% | 41.6% | 88.9% |
| Total | | Count | 387 | 335 | 722 |
|  |  | % within Grade category | 100.0% | 100.0% | 100.0% |
|  |  | % of Total | 53.6% | 46.4% | 100.0% |

| **Chi-Square Tests** | | | | | |
| --- | --- | --- | --- | --- | --- |
|  | Value | df | Asymptotic Significance (2-sided) | Exact Sig. (2-sided) | Exact Sig. (1-sided) |
| Pearson Chi-Square | .254^a^ | 1 | .614 |  |  |
| Continuity Correction^b^ | .148 | 1 | .700 |  |  |
| Likelihood Ratio | .255 | 1 | .614 |  |  |
| Fisher's Exact Test |  |  |  | .636 | .351 |
| Linear-by-Linear Association | .253 | 1 | .615 |  |  |
| N of Valid Cases | 722 |  |  |  |  |
| a. 0 cells (0.0%) have expected count less than 5. The minimum expected count is 37.12. | | | | | |
| b. Computed only for a 2x2 table | | | | | |

**Siblings (brother/sister) * School type**

| **Crosstab** | | | | | |
| --- | --- | --- | --- | --- | --- |
|  | | | School type | | Total |
|  |  |  | Public | Private |  |
| Siblings (brother/sister) | Yes | Count | 54 | 26 | 80 |
|  |  | % within School type | 12.9% | 8.6% | 11.1% |
|  |  | % of Total | 7.5% | 3.6% | 11.1% |
|  | No | Count | 366 | 276 | 642 |
|  |  | % within School type | 87.1% | 91.4% | 88.9% |
|  |  | % of Total | 50.7% | 38.2% | 88.9% |
| Total | | Count | 420 | 302 | 722 |
|  |  | % within School type | 100.0% | 100.0% | 100.0% |
|  |  | % of Total | 58.2% | 41.8% | 100.0% |

| **Chi-Square Tests** | | | | | |
| --- | --- | --- | --- | --- | --- |
|  | Value | df | Asymptotic Significance (2-sided) | Exact Sig. (2-sided) | Exact Sig. (1-sided) |
| Pearson Chi-Square | 3.217^a^ | 1 | .073 |  |  |
| Continuity Correction^b^ | 2.801 | 1 | .094 |  |  |
| Likelihood Ratio | 3.297 | 1 | .069 |  |  |
| Fisher's Exact Test |  |  |  | .092 | .046 |
| Linear-by-Linear Association | 3.213 | 1 | .073 |  |  |
| N of Valid Cases | 722 |  |  |  |  |
| a. 0 cells (0.0%) have expected count less than 5. The minimum expected count is 33.46. | | | | | |
| b. Computed only for a 2x2 table | | | | | |

**Friends/peers * Sex**

| **Crosstab** | | | | | |
| --- | --- | --- | --- | --- | --- |
|  | | | Sex | | Total |
|  |  |  | Male | Female |  |
| Friends/peers | Yes | Count | 70 | 79 | 149 |
|  |  | % within Sex | 22.2% | 19.4% | 20.6% |
|  |  | % of Total | 9.7% | 10.9% | 20.6% |
|  | No | Count | 245 | 328 | 573 |
|  |  | % within Sex | 77.8% | 80.6% | 79.4% |
|  |  | % of Total | 33.9% | 45.4% | 79.4% |
| Total | | Count | 315 | 407 | 722 |
|  |  | % within Sex | 100.0% | 100.0% | 100.0% |
|  |  | % of Total | 43.6% | 56.4% | 100.0% |

| **Chi-Square Tests** | | | | | |
| --- | --- | --- | --- | --- | --- |
|  | Value | df | Asymptotic Significance (2-sided) | Exact Sig. (2-sided) | Exact Sig. (1-sided) |
| Pearson Chi-Square | .857^a^ | 1 | .355 |  |  |
| Continuity Correction^b^ | .694 | 1 | .405 |  |  |
| Likelihood Ratio | .854 | 1 | .355 |  |  |
| Fisher's Exact Test |  |  |  | .356 | .202 |
| Linear-by-Linear Association | .856 | 1 | .355 |  |  |
| N of Valid Cases | 722 |  |  |  |  |
| a. 0 cells (0.0%) have expected count less than 5. The minimum expected count is 65.01. | | | | | |
| b. Computed only for a 2x2 table | | | | | |

**Friends/peers * Grade category**

| **Crosstab** | | | | | |
| --- | --- | --- | --- | --- | --- |
|  | | | Grade category | | Total |
|  |  |  | 9-10 | 11-12 |  |
| Friends/peers | Yes | Count | 76 | 73 | 149 |
|  |  | % within Grade category | 19.6% | 21.8% | 20.6% |
|  |  | % of Total | 10.5% | 10.1% | 20.6% |
|  | No | Count | 311 | 262 | 573 |
|  |  | % within Grade category | 80.4% | 78.2% | 79.4% |
|  |  | % of Total | 43.1% | 36.3% | 79.4% |
| Total | | Count | 387 | 335 | 722 |
|  |  | % within Grade category | 100.0% | 100.0% | 100.0% |
|  |  | % of Total | 53.6% | 46.4% | 100.0% |

| **Chi-Square Tests** | | | | | |
| --- | --- | --- | --- | --- | --- |
|  | Value | df | Asymptotic Significance (2-sided) | Exact Sig. (2-sided) | Exact Sig. (1-sided) |
| Pearson Chi-Square | .508^a^ | 1 | .476 |  |  |
| Continuity Correction^b^ | .385 | 1 | .535 |  |  |
| Likelihood Ratio | .507 | 1 | .476 |  |  |
| Fisher's Exact Test |  |  |  | .519 | .267 |
| Linear-by-Linear Association | .507 | 1 | .476 |  |  |
| N of Valid Cases | 722 |  |  |  |  |
| a. 0 cells (0.0%) have expected count less than 5. The minimum expected count is 69.13. | | | | | |
| b. Computed only for a 2x2 table | | | | | |

**Friends/peers * School type**

| **Crosstab** | | | | | |
| --- | --- | --- | --- | --- | --- |
|  | | | School type | | Total |
|  |  |  | Public | Private |  |
| Friends/peers | Yes | Count | 99 | 50 | 149 |
|  |  | % within School type | 23.6% | 16.6% | 20.6% |
|  |  | % of Total | 13.7% | 6.9% | 20.6% |
|  | No | Count | 321 | 252 | 573 |
|  |  | % within School type | 76.4% | 83.4% | 79.4% |
|  |  | % of Total | 44.5% | 34.9% | 79.4% |
| Total | | Count | 420 | 302 | 722 |
|  |  | % within School type | 100.0% | 100.0% | 100.0% |
|  |  | % of Total | 58.2% | 41.8% | 100.0% |

| **Chi-Square Tests** | | | | | |
| --- | --- | --- | --- | --- | --- |
|  | Value | df | Asymptotic Significance (2-sided) | Exact Sig. (2-sided) | Exact Sig. (1-sided) |
| Pearson Chi-Square | 5.279^a^ | 1 | .022 |  |  |
| Continuity Correction^b^ | 4.859 | 1 | .028 |  |  |
| Likelihood Ratio | 5.375 | 1 | .020 |  |  |
| Fisher's Exact Test |  |  |  | .025 | .013 |
| Linear-by-Linear Association | 5.271 | 1 | .022 |  |  |
| N of Valid Cases | 722 |  |  |  |  |
| a. 0 cells (0.0%) have expected count less than 5. The minimum expected count is 62.32. | | | | | |
| b. Computed only for a 2x2 table | | | | | |

**Health professionals * Sex**

| **Crosstab** | | | | | |
| --- | --- | --- | --- | --- | --- |
|  | | | Sex | | Total |
|  |  |  | Male | Female |  |
| Health professionals | Yes | Count | 48 | 50 | 98 |
|  |  | % within Sex | 15.2% | 12.3% | 13.6% |
|  |  | % of Total | 6.6% | 6.9% | 13.6% |
|  | No | Count | 267 | 357 | 624 |
|  |  | % within Sex | 84.8% | 87.7% | 86.4% |
|  |  | % of Total | 37.0% | 49.4% | 86.4% |
| Total | | Count | 315 | 407 | 722 |
|  |  | % within Sex | 100.0% | 100.0% | 100.0% |
|  |  | % of Total | 43.6% | 56.4% | 100.0% |

| **Chi-Square Tests** | | | | | |
| --- | --- | --- | --- | --- | --- |
|  | Value | df | Asymptotic Significance (2-sided) | Exact Sig. (2-sided) | Exact Sig. (1-sided) |
| Pearson Chi-Square | 1.320^a^ | 1 | .251 |  |  |
| Continuity Correction^b^ | 1.080 | 1 | .299 |  |  |
| Likelihood Ratio | 1.312 | 1 | .252 |  |  |
| Fisher's Exact Test |  |  |  | .274 | .149 |
| Linear-by-Linear Association | 1.318 | 1 | .251 |  |  |
| N of Valid Cases | 722 |  |  |  |  |
| a. 0 cells (0.0%) have expected count less than 5. The minimum expected count is 42.76. | | | | | |
| b. Computed only for a 2x2 table | | | | | |

**Health professionals * Grade category**

| **Crosstab** | | | | | |
| --- | --- | --- | --- | --- | --- |
|  | | | Grade category | | Total |
|  |  |  | 9-10 | 11-12 |  |
| Health professionals | Yes | Count | 57 | 41 | 98 |
|  |  | % within Grade category | 14.7% | 12.2% | 13.6% |
|  |  | % of Total | 7.9% | 5.7% | 13.6% |
|  | No | Count | 330 | 294 | 624 |
|  |  | % within Grade category | 85.3% | 87.8% | 86.4% |
|  |  | % of Total | 45.7% | 40.7% | 86.4% |
| Total | | Count | 387 | 335 | 722 |
|  |  | % within Grade category | 100.0% | 100.0% | 100.0% |
|  |  | % of Total | 53.6% | 46.4% | 100.0% |

| **Chi-Square Tests** | | | | | |
| --- | --- | --- | --- | --- | --- |
|  | Value | df | Asymptotic Significance (2-sided) | Exact Sig. (2-sided) | Exact Sig. (1-sided) |
| Pearson Chi-Square | .949^a^ | 1 | .330 |  |  |
| Continuity Correction^b^ | .749 | 1 | .387 |  |  |
| Likelihood Ratio | .954 | 1 | .329 |  |  |
| Fisher's Exact Test |  |  |  | .384 | .194 |
| Linear-by-Linear Association | .948 | 1 | .330 |  |  |
| N of Valid Cases | 722 |  |  |  |  |
| a. 0 cells (0.0%) have expected count less than 5. The minimum expected count is 45.47. | | | | | |
| b. Computed only for a 2x2 table | | | | | |

**Health professionals * School type**

| **Crosstab** | | | | | |
| --- | --- | --- | --- | --- | --- |
|  | | | School type | | Total |
|  |  |  | Public | Private |  |
| Health professionals | Yes | Count | 56 | 42 | 98 |
|  |  | % within School type | 13.3% | 13.9% | 13.6% |
|  |  | % of Total | 7.8% | 5.8% | 13.6% |
|  | No | Count | 364 | 260 | 624 |
|  |  | % within School type | 86.7% | 86.1% | 86.4% |
|  |  | % of Total | 50.4% | 36.0% | 86.4% |
| Total | | Count | 420 | 302 | 722 |
|  |  | % within School type | 100.0% | 100.0% | 100.0% |
|  |  | % of Total | 58.2% | 41.8% | 100.0% |

| **Chi-Square Tests** | | | | | |
| --- | --- | --- | --- | --- | --- |
|  | Value | df | Asymptotic Significance (2-sided) | Exact Sig. (2-sided) | Exact Sig. (1-sided) |
| Pearson Chi-Square | .049^a^ | 1 | .824 |  |  |
| Continuity Correction^b^ | .013 | 1 | .911 |  |  |
| Likelihood Ratio | .049 | 1 | .824 |  |  |
| Fisher's Exact Test |  |  |  | .827 | .454 |
| Linear-by-Linear Association | .049 | 1 | .824 |  |  |
| N of Valid Cases | 722 |  |  |  |  |
| a. 0 cells (0.0%) have expected count less than 5. The minimum expected count is 40.99. | | | | | |
| b. Computed only for a 2x2 table | | | | | |

**Teachers * Sex**

| **Crosstab** | | | | | |
| --- | --- | --- | --- | --- | --- |
|  | | | Sex | | Total |
|  |  |  | Male | Female |  |
| Teachers | Yes | Count | 4 | 12 | 16 |
|  |  | % within Sex | 1.3% | 3.0% | 2.2% |
|  |  | % of Total | 0.6% | 1.7% | 2.2% |
|  | No | Count | 311 | 394 | 705 |
|  |  | % within Sex | 98.7% | 97.0% | 97.8% |
|  |  | % of Total | 43.1% | 54.6% | 97.8% |
| Total | | Count | 315 | 406 | 721 |
|  |  | % within Sex | 100.0% | 100.0% | 100.0% |
|  |  | % of Total | 43.7% | 56.3% | 100.0% |

| **Chi-Square Tests** | | | | | |
| --- | --- | --- | --- | --- | --- |
|  | Value | df | Asymptotic Significance (2-sided) | Exact Sig. (2-sided) | Exact Sig. (1-sided) |
| Pearson Chi-Square | 2.323^a^ | 1 | .127 |  |  |
| Continuity Correction^b^ | 1.611 | 1 | .204 |  |  |
| Likelihood Ratio | 2.464 | 1 | .116 |  |  |
| Fisher's Exact Test |  |  |  | .201 | .100 |
| Linear-by-Linear Association | 2.320 | 1 | .128 |  |  |
| N of Valid Cases | 721 |  |  |  |  |
| a. 0 cells (0.0%) have expected count less than 5. The minimum expected count is 6.99. | | | | | |
| b. Computed only for a 2x2 table | | | | | |

**Teachers * Grade category**

| **Crosstab** | | | | | |
| --- | --- | --- | --- | --- | --- |
|  | | | Grade category | | Total |
|  |  |  | 9-10 | 11-12 |  |
| Teachers | Yes | Count | 8 | 8 | 16 |
|  |  | % within Grade category | 2.1% | 2.4% | 2.2% |
|  |  | % of Total | 1.1% | 1.1% | 2.2% |
|  | No | Count | 378 | 327 | 705 |
|  |  | % within Grade category | 97.9% | 97.6% | 97.8% |
|  |  | % of Total | 52.4% | 45.4% | 97.8% |
| Total | | Count | 386 | 335 | 721 |
|  |  | % within Grade category | 100.0% | 100.0% | 100.0% |
|  |  | % of Total | 53.5% | 46.5% | 100.0% |

| **Chi-Square Tests** | | | | | |
| --- | --- | --- | --- | --- | --- |
|  | Value | df | Asymptotic Significance (2-sided) | Exact Sig. (2-sided) | Exact Sig. (1-sided) |
| Pearson Chi-Square | .082^a^ | 1 | .774 |  |  |
| Continuity Correction^b^ | .001 | 1 | .973 |  |  |
| Likelihood Ratio | .082 | 1 | .774 |  |  |
| Fisher's Exact Test |  |  |  | .805 | .484 |
| Linear-by-Linear Association | .082 | 1 | .774 |  |  |
| N of Valid Cases | 721 |  |  |  |  |
| a. 0 cells (0.0%) have expected count less than 5. The minimum expected count is 7.43. | | | | | |
| b. Computed only for a 2x2 table | | | | | |

**Teachers * School type**

| **Crosstab** | | | | | |
| --- | --- | --- | --- | --- | --- |
|  | | | School type | | Total |
|  |  |  | Public | Private |  |
| Teachers | Yes | Count | 10 | 6 | 16 |
|  |  | % within School type | 2.4% | 2.0% | 2.2% |
|  |  | % of Total | 1.4% | 0.8% | 2.2% |
|  | No | Count | 410 | 295 | 705 |
|  |  | % within School type | 97.6% | 98.0% | 97.8% |
|  |  | % of Total | 56.9% | 40.9% | 97.8% |
| Total | | Count | 420 | 301 | 721 |
|  |  | % within School type | 100.0% | 100.0% | 100.0% |
|  |  | % of Total | 58.3% | 41.7% | 100.0% |

| **Chi-Square Tests** | | | | | |
| --- | --- | --- | --- | --- | --- |
|  | Value | df | Asymptotic Significance (2-sided) | Exact Sig. (2-sided) | Exact Sig. (1-sided) |
| Pearson Chi-Square | .121^a^ | 1 | .728 |  |  |
| Continuity Correction^b^ | .008 | 1 | .927 |  |  |
| Likelihood Ratio | .123 | 1 | .726 |  |  |
| Fisher's Exact Test |  |  |  | .803 | .469 |
| Linear-by-Linear Association | .121 | 1 | .728 |  |  |
| N of Valid Cases | 721 |  |  |  |  |
| a. 0 cells (0.0%) have expected count less than 5. The minimum expected count is 6.68. | | | | | |
| b. Computed only for a 2x2 table | | | | | |

**Internet * Sex**

| **Crosstab** | | | | | |
| --- | --- | --- | --- | --- | --- |
|  | | | Sex | | Total |
|  |  |  | Male | Female |  |
| Internet | Yes | Count | 60 | 65 | 125 |
|  |  | % within Sex | 19.0% | 16.0% | 17.3% |
|  |  | % of Total | 8.3% | 9.0% | 17.3% |
|  | No | Count | 255 | 342 | 597 |
|  |  | % within Sex | 81.0% | 84.0% | 82.7% |
|  |  | % of Total | 35.3% | 47.4% | 82.7% |
| Total | | Count | 315 | 407 | 722 |
|  |  | % within Sex | 100.0% | 100.0% | 100.0% |
|  |  | % of Total | 43.6% | 56.4% | 100.0% |

| **Chi-Square Tests** | | | | | |
| --- | --- | --- | --- | --- | --- |
|  | Value | df | Asymptotic Significance (2-sided) | Exact Sig. (2-sided) | Exact Sig. (1-sided) |
| Pearson Chi-Square | 1.174^a^ | 1 | .278 |  |  |
| Continuity Correction^b^ | .969 | 1 | .325 |  |  |
| Likelihood Ratio | 1.169 | 1 | .280 |  |  |
| Fisher's Exact Test |  |  |  | .321 | .162 |
| Linear-by-Linear Association | 1.173 | 1 | .279 |  |  |
| N of Valid Cases | 722 |  |  |  |  |
| a. 0 cells (0.0%) have expected count less than 5. The minimum expected count is 54.54. | | | | | |
| b. Computed only for a 2x2 table | | | | | |

**Internet * Grade category**

| **Crosstab** | | | | | |
| --- | --- | --- | --- | --- | --- |
|  | | | Grade category | | Total |
|  |  |  | 9-10 | 11-12 |  |
| Internet | Yes | Count | 45 | 80 | 125 |
|  |  | % within Grade category | 11.6% | 23.9% | 17.3% |
|  |  | % of Total | 6.2% | 11.1% | 17.3% |
|  | No | Count | 342 | 255 | 597 |
|  |  | % within Grade category | 88.4% | 76.1% | 82.7% |
|  |  | % of Total | 47.4% | 35.3% | 82.7% |
| Total | | Count | 387 | 335 | 722 |
|  |  | % within Grade category | 100.0% | 100.0% | 100.0% |
|  |  | % of Total | 53.6% | 46.4% | 100.0% |

| **Chi-Square Tests** | | | | | |
| --- | --- | --- | --- | --- | --- |
|  | Value | df | Asymptotic Significance (2-sided) | Exact Sig. (2-sided) | Exact Sig. (1-sided) |
| Pearson Chi-Square | 18.831^a^ | 1 | .000 |  |  |
| Continuity Correction^b^ | 17.985 | 1 | .000 |  |  |
| Likelihood Ratio | 18.908 | 1 | .000 |  |  |
| Fisher's Exact Test |  |  |  | .000 | .000 |
| Linear-by-Linear Association | 18.805 | 1 | .000 |  |  |
| N of Valid Cases | 722 |  |  |  |  |
| a. 0 cells (0.0%) have expected count less than 5. The minimum expected count is 58.00. | | | | | |
| b. Computed only for a 2x2 table | | | | | |

**Internet * School type**

| **Crosstab** | | | | | |
| --- | --- | --- | --- | --- | --- |
|  | | | School type | | Total |
|  |  |  | Public | Private |  |
| Internet | Yes | Count | 36 | 89 | 125 |
|  |  | % within School type | 8.6% | 29.5% | 17.3% |
|  |  | % of Total | 5.0% | 12.3% | 17.3% |
|  | No | Count | 384 | 213 | 597 |
|  |  | % within School type | 91.4% | 70.5% | 82.7% |
|  |  | % of Total | 53.2% | 29.5% | 82.7% |
| Total | | Count | 420 | 302 | 722 |
|  |  | % within School type | 100.0% | 100.0% | 100.0% |
|  |  | % of Total | 58.2% | 41.8% | 100.0% |

| **Chi-Square Tests** | | | | | |
| --- | --- | --- | --- | --- | --- |
|  | Value | df | Asymptotic Significance (2-sided) | Exact Sig. (2-sided) | Exact Sig. (1-sided) |
| Pearson Chi-Square | 53.598^a^ | 1 | .000 |  |  |
| Continuity Correction^b^ | 52.148 | 1 | .000 |  |  |
| Likelihood Ratio | 53.500 | 1 | .000 |  |  |
| Fisher's Exact Test |  |  |  | .000 | .000 |
| Linear-by-Linear Association | 53.524 | 1 | .000 |  |  |
| N of Valid Cases | 722 |  |  |  |  |
| a. 0 cells (0.0%) have expected count less than 5. The minimum expected count is 52.29. | | | | | |
| b. Computed only for a 2x2 table | | | | | |

**Religious figures * Sex**

| **Crosstab** | | | | | |
| --- | --- | --- | --- | --- | --- |
|  | | | Sex | | Total |
|  |  |  | Male | Female |  |
| Religious figures | Yes | Count | 11 | 7 | 18 |
|  |  | % within Sex | 3.5% | 1.7% | 2.5% |
|  |  | % of Total | 1.5% | 1.0% | 2.5% |
|  | No | Count | 304 | 400 | 704 |
|  |  | % within Sex | 96.5% | 98.3% | 97.5% |
|  |  | % of Total | 42.1% | 55.4% | 97.5% |
| Total | | Count | 315 | 407 | 722 |
|  |  | % within Sex | 100.0% | 100.0% | 100.0% |
|  |  | % of Total | 43.6% | 56.4% | 100.0% |

| **Chi-Square Tests** | | | | | |
| --- | --- | --- | --- | --- | --- |
|  | Value | df | Asymptotic Significance (2-sided) | Exact Sig. (2-sided) | Exact Sig. (1-sided) |
| Pearson Chi-Square | 2.294^a^ | 1 | .130 |  |  |
| Continuity Correction^b^ | 1.623 | 1 | .203 |  |  |
| Likelihood Ratio | 2.273 | 1 | .132 |  |  |
| Fisher's Exact Test |  |  |  | .152 | .102 |
| Linear-by-Linear Association | 2.291 | 1 | .130 |  |  |
| N of Valid Cases | 722 |  |  |  |  |
| a. 0 cells (0.0%) have expected count less than 5. The minimum expected count is 7.85. | | | | | |
| b. Computed only for a 2x2 table | | | | | |

**Religious figures * Grade category**

| **Crosstab** | | | | | |
| --- | --- | --- | --- | --- | --- |
|  | | | Grade category | | Total |
|  |  |  | 9-10 | 11-12 |  |
| Religious figures | Yes | Count | 8 | 10 | 18 |
|  |  | % within Grade category | 2.1% | 3.0% | 2.5% |
|  |  | % of Total | 1.1% | 1.4% | 2.5% |
|  | No | Count | 379 | 325 | 704 |
|  |  | % within Grade category | 97.9% | 97.0% | 97.5% |
|  |  | % of Total | 52.5% | 45.0% | 97.5% |
| Total | | Count | 387 | 335 | 722 |
|  |  | % within Grade category | 100.0% | 100.0% | 100.0% |
|  |  | % of Total | 53.6% | 46.4% | 100.0% |

| **Chi-Square Tests** | | | | | |
| --- | --- | --- | --- | --- | --- |
|  | Value | df | Asymptotic Significance (2-sided) | Exact Sig. (2-sided) | Exact Sig. (1-sided) |
| Pearson Chi-Square | .622^a^ | 1 | .430 |  |  |
| Continuity Correction^b^ | .302 | 1 | .583 |  |  |
| Likelihood Ratio | .620 | 1 | .431 |  |  |
| Fisher's Exact Test |  |  |  | .479 | .291 |
| Linear-by-Linear Association | .621 | 1 | .430 |  |  |
| N of Valid Cases | 722 |  |  |  |  |
| a. 0 cells (0.0%) have expected count less than 5. The minimum expected count is 8.35. | | | | | |
| b. Computed only for a 2x2 table | | | | | |

**Religious figures * School type**

| **Crosstab** | | | | | |
| --- | --- | --- | --- | --- | --- |
|  | | | School type | | Total |
|  |  |  | Public | Private |  |
| Religious figures | Yes | Count | 11 | 7 | 18 |
|  |  | % within School type | 2.6% | 2.3% | 2.5% |
|  |  | % of Total | 1.5% | 1.0% | 2.5% |
|  | No | Count | 409 | 295 | 704 |
|  |  | % within School type | 97.4% | 97.7% | 97.5% |
|  |  | % of Total | 56.6% | 40.9% | 97.5% |
| Total | | Count | 420 | 302 | 722 |
|  |  | % within School type | 100.0% | 100.0% | 100.0% |
|  |  | % of Total | 58.2% | 41.8% | 100.0% |

| **Chi-Square Tests** | | | | | |
| --- | --- | --- | --- | --- | --- |
|  | Value | df | Asymptotic Significance (2-sided) | Exact Sig. (2-sided) | Exact Sig. (1-sided) |
| Pearson Chi-Square | .066^a^ | 1 | .798 |  |  |
| Continuity Correction^b^ | .000 | 1 | .989 |  |  |
| Likelihood Ratio | .066 | 1 | .797 |  |  |
| Fisher's Exact Test |  |  |  | 1.000 | .500 |
| Linear-by-Linear Association | .065 | 1 | .798 |  |  |
| N of Valid Cases | 722 |  |  |  |  |
| a. 0 cells (0.0%) have expected count less than 5. The minimum expected count is 7.53. | | | | | |
| b. Computed only for a 2x2 table | | | | | |

**12. Trusted sources of health information:** Which source/s of health information do you trust more for reliable information?

**Health professionals * Sex**

| **Crosstab** | | | | | |
| --- | --- | --- | --- | --- | --- |
|  | | | Sex | | Total |
|  |  |  | Male | Female |  |
| Health professionals | Yes | Count | 230 | 322 | 552 |
|  |  | % within Sex | 73.0% | 79.1% | 76.5% |
|  |  | % of Total | 31.9% | 44.6% | 76.5% |
|  | No | Count | 85 | 85 | 170 |
|  |  | % within Sex | 27.0% | 20.9% | 23.5% |
|  |  | % of Total | 11.8% | 11.8% | 23.5% |
| Total | | Count | 315 | 407 | 722 |
|  |  | % within Sex | 100.0% | 100.0% | 100.0% |
|  |  | % of Total | 43.6% | 56.4% | 100.0% |

| **Chi-Square Tests** | | | | | |
| --- | --- | --- | --- | --- | --- |
|  | Value | df | Asymptotic Significance (2-sided) | Exact Sig. (2-sided) | Exact Sig. (1-sided) |
| Pearson Chi-Square | 3.670^a^ | 1 | .055 |  |  |
| Continuity Correction^b^ | 3.339 | 1 | .068 |  |  |
| Likelihood Ratio | 3.650 | 1 | .056 |  |  |
| Fisher's Exact Test |  |  |  | .063 | .034 |
| Linear-by-Linear Association | 3.665 | 1 | .056 |  |  |
| N of Valid Cases | 722 |  |  |  |  |
| a. 0 cells (0.0%) have expected count less than 5. The minimum expected count is 74.17. | | | | | |
| b. Computed only for a 2x2 table | | | | | |

**Health professionals * Grade category**

| **Crosstab** | | | | | |
| --- | --- | --- | --- | --- | --- |
|  | | | Grade category | | Total |
|  |  |  | 9-10 | 11-12 |  |
| Health professionals | Yes | Count | 301 | 251 | 552 |
|  |  | % within Grade category | 77.8% | 74.9% | 76.5% |
|  |  | % of Total | 41.7% | 34.8% | 76.5% |
|  | No | Count | 86 | 84 | 170 |
|  |  | % within Grade category | 22.2% | 25.1% | 23.5% |
|  |  | % of Total | 11.9% | 11.6% | 23.5% |
| Total | | Count | 387 | 335 | 722 |
|  |  | % within Grade category | 100.0% | 100.0% | 100.0% |
|  |  | % of Total | 53.6% | 46.4% | 100.0% |

| **Chi-Square Tests** | | | | | |
| --- | --- | --- | --- | --- | --- |
|  | Value | df | Asymptotic Significance (2-sided) | Exact Sig. (2-sided) | Exact Sig. (1-sided) |
| Pearson Chi-Square | .812^a^ | 1 | .368 |  |  |
| Continuity Correction^b^ | .661 | 1 | .416 |  |  |
| Likelihood Ratio | .810 | 1 | .368 |  |  |
| Fisher's Exact Test |  |  |  | .380 | .208 |
| Linear-by-Linear Association | .810 | 1 | .368 |  |  |
| N of Valid Cases | 722 |  |  |  |  |
| a. 0 cells (0.0%) have expected count less than 5. The minimum expected count is 78.88. | | | | | |
| b. Computed only for a 2x2 table | | | | | |

**Health professionals * School type**

| **Crosstab** | | | | | |
| --- | --- | --- | --- | --- | --- |
|  | | | School type | | Total |
|  |  |  | Public | Private |  |
| Health professionals | Yes | Count | 328 | 224 | 552 |
|  |  | % within School type | 78.1% | 74.2% | 76.5% |
|  |  | % of Total | 45.4% | 31.0% | 76.5% |
|  | No | Count | 92 | 78 | 170 |
|  |  | % within School type | 21.9% | 25.8% | 23.5% |
|  |  | % of Total | 12.7% | 10.8% | 23.5% |
| Total | | Count | 420 | 302 | 722 |
|  |  | % within School type | 100.0% | 100.0% | 100.0% |
|  |  | % of Total | 58.2% | 41.8% | 100.0% |

| **Chi-Square Tests** | | | | | |
| --- | --- | --- | --- | --- | --- |
|  | Value | df | Asymptotic Significance (2-sided) | Exact Sig. (2-sided) | Exact Sig. (1-sided) |
| Pearson Chi-Square | 1.502^a^ | 1 | .220 |  |  |
| Continuity Correction^b^ | 1.292 | 1 | .256 |  |  |
| Likelihood Ratio | 1.494 | 1 | .222 |  |  |
| Fisher's Exact Test |  |  |  | .248 | .128 |
| Linear-by-Linear Association | 1.500 | 1 | .221 |  |  |
| N of Valid Cases | 722 |  |  |  |  |
| a. 0 cells (0.0%) have expected count less than 5. The minimum expected count is 71.11. | | | | | |
| b. Computed only for a 2x2 table | | | | | |

**Books * Sex**

| **Crosstab** | | | | | |
| --- | --- | --- | --- | --- | --- |
|  | | | Sex | | Total |
|  |  |  | Male | Female |  |
| Books | Yes | Count | 51 | 61 | 112 |
|  |  | % within Sex | 16.2% | 15.0% | 15.5% |
|  |  | % of Total | 7.1% | 8.4% | 15.5% |
|  | No | Count | 264 | 346 | 610 |
|  |  | % within Sex | 83.8% | 85.0% | 84.5% |
|  |  | % of Total | 36.6% | 47.9% | 84.5% |
| Total | | Count | 315 | 407 | 722 |
|  |  | % within Sex | 100.0% | 100.0% | 100.0% |
|  |  | % of Total | 43.6% | 56.4% | 100.0% |

| **Chi-Square Tests** | | | | | |
| --- | --- | --- | --- | --- | --- |
|  | Value | df | Asymptotic Significance (2-sided) | Exact Sig. (2-sided) | Exact Sig. (1-sided) |
| Pearson Chi-Square | .196^a^ | 1 | .658 |  |  |
| Continuity Correction^b^ | .115 | 1 | .735 |  |  |
| Likelihood Ratio | .196 | 1 | .658 |  |  |
| Fisher's Exact Test |  |  |  | .679 | .366 |
| Linear-by-Linear Association | .196 | 1 | .658 |  |  |
| N of Valid Cases | 722 |  |  |  |  |
| a. 0 cells (0.0%) have expected count less than 5. The minimum expected count is 48.86. | | | | | |
| b. Computed only for a 2x2 table | | | | | |

**Books * Grade category**

| **Crosstab** | | | | | |
| --- | --- | --- | --- | --- | --- |
|  | | | Grade category | | Total |
|  |  |  | 9-10 | 11-12 |  |
| Books | Yes | Count | 54 | 58 | 112 |
|  |  | % within Grade category | 14.0% | 17.3% | 15.5% |
|  |  | % of Total | 7.5% | 8.0% | 15.5% |
|  | No | Count | 333 | 277 | 610 |
|  |  | % within Grade category | 86.0% | 82.7% | 84.5% |
|  |  | % of Total | 46.1% | 38.4% | 84.5% |
| Total | | Count | 387 | 335 | 722 |
|  |  | % within Grade category | 100.0% | 100.0% | 100.0% |
|  |  | % of Total | 53.6% | 46.4% | 100.0% |

| **Chi-Square Tests** | | | | | |
| --- | --- | --- | --- | --- | --- |
|  | Value | df | Asymptotic Significance (2-sided) | Exact Sig. (2-sided) | Exact Sig. (1-sided) |
| Pearson Chi-Square | 1.547^a^ | 1 | .214 |  |  |
| Continuity Correction^b^ | 1.301 | 1 | .254 |  |  |
| Likelihood Ratio | 1.543 | 1 | .214 |  |  |
| Fisher's Exact Test |  |  |  | .218 | .127 |
| Linear-by-Linear Association | 1.545 | 1 | .214 |  |  |
| N of Valid Cases | 722 |  |  |  |  |
| a. 0 cells (0.0%) have expected count less than 5. The minimum expected count is 51.97. | | | | | |
| b. Computed only for a 2x2 table | | | | | |

**Books * School type**

| **Crosstab** | | | | | |
| --- | --- | --- | --- | --- | --- |
|  | | | School type | | Total |
|  |  |  | Public | Private |  |
| Books | Yes | Count | 57 | 55 | 112 |
|  |  | % within School type | 13.6% | 18.2% | 15.5% |
|  |  | % of Total | 7.9% | 7.6% | 15.5% |
|  | No | Count | 363 | 247 | 610 |
|  |  | % within School type | 86.4% | 81.8% | 84.5% |
|  |  | % of Total | 50.3% | 34.2% | 84.5% |
| Total | | Count | 420 | 302 | 722 |
|  |  | % within School type | 100.0% | 100.0% | 100.0% |
|  |  | % of Total | 58.2% | 41.8% | 100.0% |

| **Chi-Square Tests** | | | | | |
| --- | --- | --- | --- | --- | --- |
|  | Value | df | Asymptotic Significance (2-sided) | Exact Sig. (2-sided) | Exact Sig. (1-sided) |
| Pearson Chi-Square | 2.887^a^ | 1 | .089 |  |  |
| Continuity Correction^b^ | 2.543 | 1 | .111 |  |  |
| Likelihood Ratio | 2.858 | 1 | .091 |  |  |
| Fisher's Exact Test |  |  |  | .096 | .056 |
| Linear-by-Linear Association | 2.883 | 1 | .090 |  |  |
| N of Valid Cases | 722 |  |  |  |  |
| a. 0 cells (0.0%) have expected count less than 5. The minimum expected count is 46.85. | | | | | |
| b. Computed only for a 2x2 table | | | | | |

**Internet * Sex**

| **Crosstab** | | | | | |
| --- | --- | --- | --- | --- | --- |
|  | | | Sex | | Total |
|  |  |  | Male | Female |  |
| Internet | Yes | Count | 86 | 79 | 165 |
|  |  | % within Sex | 27.3% | 19.4% | 22.9% |
|  |  | % of Total | 11.9% | 10.9% | 22.9% |
|  | No | Count | 229 | 328 | 557 |
|  |  | % within Sex | 72.7% | 80.6% | 77.1% |
|  |  | % of Total | 31.7% | 45.4% | 77.1% |
| Total | | Count | 315 | 407 | 722 |
|  |  | % within Sex | 100.0% | 100.0% | 100.0% |
|  |  | % of Total | 43.6% | 56.4% | 100.0% |

| **Chi-Square Tests** | | | | | |
| --- | --- | --- | --- | --- | --- |
|  | Value | df | Asymptotic Significance (2-sided) | Exact Sig. (2-sided) | Exact Sig. (1-sided) |
| Pearson Chi-Square | 6.272^a^ | 1 | .012 |  |  |
| Continuity Correction^b^ | 5.832 | 1 | .016 |  |  |
| Likelihood Ratio | 6.232 | 1 | .013 |  |  |
| Fisher's Exact Test |  |  |  | .016 | .008 |
| Linear-by-Linear Association | 6.263 | 1 | .012 |  |  |
| N of Valid Cases | 722 |  |  |  |  |
| a. 0 cells (0.0%) have expected count less than 5. The minimum expected count is 71.99. | | | | | |
| b. Computed only for a 2x2 table | | | | | |

**Internet * Grade category**

| **Crosstab** | | | | | |
| --- | --- | --- | --- | --- | --- |
|  | | | Grade category | | Total |
|  |  |  | 9-10 | 11-12 |  |
| Internet | Yes | Count | 70 | 95 | 165 |
|  |  | % within Grade category | 18.1% | 28.4% | 22.9% |
|  |  | % of Total | 9.7% | 13.2% | 22.9% |
|  | No | Count | 317 | 240 | 557 |
|  |  | % within Grade category | 81.9% | 71.6% | 77.1% |
|  |  | % of Total | 43.9% | 33.2% | 77.1% |
| Total | | Count | 387 | 335 | 722 |
|  |  | % within Grade category | 100.0% | 100.0% | 100.0% |
|  |  | % of Total | 53.6% | 46.4% | 100.0% |

| **Chi-Square Tests** | | | | | |
| --- | --- | --- | --- | --- | --- |
|  | Value | df | Asymptotic Significance (2-sided) | Exact Sig. (2-sided) | Exact Sig. (1-sided) |
| Pearson Chi-Square | 10.743^a^ | 1 | .001 |  |  |
| Continuity Correction^b^ | 10.168 | 1 | .001 |  |  |
| Likelihood Ratio | 10.733 | 1 | .001 |  |  |
| Fisher's Exact Test |  |  |  | .001 | .001 |
| Linear-by-Linear Association | 10.728 | 1 | .001 |  |  |
| N of Valid Cases | 722 |  |  |  |  |
| a. 0 cells (0.0%) have expected count less than 5. The minimum expected count is 76.56. | | | | | |
| b. Computed only for a 2x2 table | | | | | |

**Internet * School type**

| **Crosstab** | | | | | |
| --- | --- | --- | --- | --- | --- |
|  | | | School type | | Total |
|  |  |  | Public | Private |  |
| Internet | Yes | Count | 51 | 114 | 165 |
|  |  | % within School type | 12.1% | 37.7% | 22.9% |
|  |  | % of Total | 7.1% | 15.8% | 22.9% |
|  | No | Count | 369 | 188 | 557 |
|  |  | % within School type | 87.9% | 62.3% | 77.1% |
|  |  | % of Total | 51.1% | 26.0% | 77.1% |
| Total | | Count | 420 | 302 | 722 |
|  |  | % within School type | 100.0% | 100.0% | 100.0% |
|  |  | % of Total | 58.2% | 41.8% | 100.0% |

| **Chi-Square Tests** | | | | | |
| --- | --- | --- | --- | --- | --- |
|  | Value | df | Asymptotic Significance (2-sided) | Exact Sig. (2-sided) | Exact Sig. (1-sided) |
| Pearson Chi-Square | 65.331^a^ | 1 | .000 |  |  |
| Continuity Correction^b^ | 63.887 | 1 | .000 |  |  |
| Likelihood Ratio | 65.202 | 1 | .000 |  |  |
| Fisher's Exact Test |  |  |  | .000 | .000 |
| Linear-by-Linear Association | 65.241 | 1 | .000 |  |  |
| N of Valid Cases | 722 |  |  |  |  |
| a. 0 cells (0.0%) have expected count less than 5. The minimum expected count is 69.02. | | | | | |
| b. Computed only for a 2x2 table | | | | | |

**Television * Sex**

| **Crosstab** | | | | | |
| --- | --- | --- | --- | --- | --- |
|  | | | Sex | | Total |
|  |  |  | Male | Female |  |
| Television | Yes | Count | 60 | 61 | 121 |
|  |  | % within Sex | 19.0% | 15.0% | 16.8% |
|  |  | % of Total | 8.3% | 8.4% | 16.8% |
|  | No | Count | 255 | 346 | 601 |
|  |  | % within Sex | 81.0% | 85.0% | 83.2% |
|  |  | % of Total | 35.3% | 47.9% | 83.2% |
| Total | | Count | 315 | 407 | 722 |
|  |  | % within Sex | 100.0% | 100.0% | 100.0% |
|  |  | % of Total | 43.6% | 56.4% | 100.0% |

| **Chi-Square Tests** | | | | | |
| --- | --- | --- | --- | --- | --- |
|  | Value | df | Asymptotic Significance (2-sided) | Exact Sig. (2-sided) | Exact Sig. (1-sided) |
| Pearson Chi-Square | 2.098^a^ | 1 | .147 |  |  |
| Continuity Correction^b^ | 1.817 | 1 | .178 |  |  |
| Likelihood Ratio | 2.085 | 1 | .149 |  |  |
| Fisher's Exact Test |  |  |  | .160 | .089 |
| Linear-by-Linear Association | 2.095 | 1 | .148 |  |  |
| N of Valid Cases | 722 |  |  |  |  |
| a. 0 cells (0.0%) have expected count less than 5. The minimum expected count is 52.79. | | | | | |
| b. Computed only for a 2x2 table | | | | | |

**Television * Grade category**

| **Crosstab** | | | | | |
| --- | --- | --- | --- | --- | --- |
|  | | | Grade category | | Total |
|  |  |  | 9-10 | 11-12 |  |
| Television | Yes | Count | 66 | 55 | 121 |
|  |  | % within Grade category | 17.1% | 16.4% | 16.8% |
|  |  | % of Total | 9.1% | 7.6% | 16.8% |
|  | No | Count | 321 | 280 | 601 |
|  |  | % within Grade category | 82.9% | 83.6% | 83.2% |
|  |  | % of Total | 44.5% | 38.8% | 83.2% |
| Total | | Count | 387 | 335 | 722 |
|  |  | % within Grade category | 100.0% | 100.0% | 100.0% |
|  |  | % of Total | 53.6% | 46.4% | 100.0% |

| **Chi-Square Tests** | | | | | |
| --- | --- | --- | --- | --- | --- |
|  | Value | df | Asymptotic Significance (2-sided) | Exact Sig. (2-sided) | Exact Sig. (1-sided) |
| Pearson Chi-Square | .052^a^ | 1 | .819 |  |  |
| Continuity Correction^b^ | .016 | 1 | .898 |  |  |
| Likelihood Ratio | .052 | 1 | .819 |  |  |
| Fisher's Exact Test |  |  |  | .842 | .450 |
| Linear-by-Linear Association | .052 | 1 | .820 |  |  |
| N of Valid Cases | 722 |  |  |  |  |
| a. 0 cells (0.0%) have expected count less than 5. The minimum expected count is 56.14. | | | | | |
| b. Computed only for a 2x2 table | | | | | |

**Television * School type**

| **Crosstab** | | | | | |
| --- | --- | --- | --- | --- | --- |
|  | | | School type | | Total |
|  |  |  | Public | Private |  |
| Television | Yes | Count | 79 | 42 | 121 |
|  |  | % within School type | 18.8% | 13.9% | 16.8% |
|  |  | % of Total | 10.9% | 5.8% | 16.8% |
|  | No | Count | 341 | 260 | 601 |
|  |  | % within School type | 81.2% | 86.1% | 83.2% |
|  |  | % of Total | 47.2% | 36.0% | 83.2% |
| Total | | Count | 420 | 302 | 722 |
|  |  | % within School type | 100.0% | 100.0% | 100.0% |
|  |  | % of Total | 58.2% | 41.8% | 100.0% |

| **Chi-Square Tests** | | | | | |
| --- | --- | --- | --- | --- | --- |
|  | Value | df | Asymptotic Significance (2-sided) | Exact Sig. (2-sided) | Exact Sig. (1-sided) |
| Pearson Chi-Square | 3.026^a^ | 1 | .082 |  |  |
| Continuity Correction^b^ | 2.685 | 1 | .101 |  |  |
| Likelihood Ratio | 3.075 | 1 | .079 |  |  |
| Fisher's Exact Test |  |  |  | .087 | .050 |
| Linear-by-Linear Association | 3.022 | 1 | .082 |  |  |
| N of Valid Cases | 722 |  |  |  |  |
| a. 0 cells (0.0%) have expected count less than 5. The minimum expected count is 50.61. | | | | | |
| b. Computed only for a 2x2 table | | | | | |

**Family * Sex**

| **Crosstab** | | | | | |
| --- | --- | --- | --- | --- | --- |
|  | | | Sex | | Total |
|  |  |  | Male | Female |  |
| Family | Yes | Count | 50 | 65 | 115 |
|  |  | % within Sex | 15.9% | 16.0% | 15.9% |
|  |  | % of Total | 6.9% | 9.0% | 15.9% |
|  | No | Count | 265 | 342 | 607 |
|  |  | % within Sex | 84.1% | 84.0% | 84.1% |
|  |  | % of Total | 36.7% | 47.4% | 84.1% |
| Total | | Count | 315 | 407 | 722 |
|  |  | % within Sex | 100.0% | 100.0% | 100.0% |
|  |  | % of Total | 43.6% | 56.4% | 100.0% |

| **Chi-Square Tests** | | | | | |
| --- | --- | --- | --- | --- | --- |
|  | Value | df | Asymptotic Significance (2-sided) | Exact Sig. (2-sided) | Exact Sig. (1-sided) |
| Pearson Chi-Square | .001^a^ | 1 | .972 |  |  |
| Continuity Correction^b^ | .000 | 1 | 1.000 |  |  |
| Likelihood Ratio | .001 | 1 | .972 |  |  |
| Fisher's Exact Test |  |  |  | 1.000 | .528 |
| Linear-by-Linear Association | .001 | 1 | .972 |  |  |
| N of Valid Cases | 722 |  |  |  |  |
| a. 0 cells (0.0%) have expected count less than 5. The minimum expected count is 50.17. | | | | | |
| b. Computed only for a 2x2 table | | | | | |

**Family * Grade category**

| **Crosstab** | | | | | |
| --- | --- | --- | --- | --- | --- |
|  | | | Grade category | | Total |
|  |  |  | 9-10 | 11-12 |  |
| Family | Yes | Count | 69 | 46 | 115 |
|  |  | % within Grade category | 17.8% | 13.7% | 15.9% |
|  |  | % of Total | 9.6% | 6.4% | 15.9% |
|  | No | Count | 318 | 289 | 607 |
|  |  | % within Grade category | 82.2% | 86.3% | 84.1% |
|  |  | % of Total | 44.0% | 40.0% | 84.1% |
| Total | | Count | 387 | 335 | 722 |
|  |  | % within Grade category | 100.0% | 100.0% | 100.0% |
|  |  | % of Total | 53.6% | 46.4% | 100.0% |

| **Chi-Square Tests** | | | | | |
| --- | --- | --- | --- | --- | --- |
|  | Value | df | Asymptotic Significance (2-sided) | Exact Sig. (2-sided) | Exact Sig. (1-sided) |
| Pearson Chi-Square | 2.252^a^ | 1 | .133 |  |  |
| Continuity Correction^b^ | 1.956 | 1 | .162 |  |  |
| Likelihood Ratio | 2.269 | 1 | .132 |  |  |
| Fisher's Exact Test |  |  |  | .153 | .081 |
| Linear-by-Linear Association | 2.249 | 1 | .134 |  |  |
| N of Valid Cases | 722 |  |  |  |  |
| a. 0 cells (0.0%) have expected count less than 5. The minimum expected count is 53.36. | | | | | |
| b. Computed only for a 2x2 table | | | | | |

**Family * School type**

| **Crosstab** | | | | | |
| --- | --- | --- | --- | --- | --- |
|  | | | School type | | Total |
|  |  |  | Public | Private |  |
| Family | Yes | Count | 43 | 72 | 115 |
|  |  | % within School type | 10.2% | 23.8% | 15.9% |
|  |  | % of Total | 6.0% | 10.0% | 15.9% |
|  | No | Count | 377 | 230 | 607 |
|  |  | % within School type | 89.8% | 76.2% | 84.1% |
|  |  | % of Total | 52.2% | 31.9% | 84.1% |
| Total | | Count | 420 | 302 | 722 |
|  |  | % within School type | 100.0% | 100.0% | 100.0% |
|  |  | % of Total | 58.2% | 41.8% | 100.0% |

| **Chi-Square Tests** | | | | | |
| --- | --- | --- | --- | --- | --- |
|  | Value | df | Asymptotic Significance (2-sided) | Exact Sig. (2-sided) | Exact Sig. (1-sided) |
| Pearson Chi-Square | 24.276^a^ | 1 | .000 |  |  |
| Continuity Correction^b^ | 23.271 | 1 | .000 |  |  |
| Likelihood Ratio | 23.977 | 1 | .000 |  |  |
| Fisher's Exact Test |  |  |  | .000 | .000 |
| Linear-by-Linear Association | 24.242 | 1 | .000 |  |  |
| N of Valid Cases | 722 |  |  |  |  |
| a. 0 cells (0.0%) have expected count less than 5. The minimum expected count is 48.10. | | | | | |
| b. Computed only for a 2x2 table | | | | | |

**Friends/peers * Sex**

| **Crosstab** | | | | | |
| --- | --- | --- | --- | --- | --- |
|  | | | Sex | | Total |
|  |  |  | Male | Female |  |
| Friends/peers | Yes | Count | 8 | 11 | 19 |
|  |  | % within Sex | 2.5% | 2.7% | 2.6% |
|  |  | % of Total | 1.1% | 1.5% | 2.6% |
|  | No | Count | 307 | 396 | 703 |
|  |  | % within Sex | 97.5% | 97.3% | 97.4% |
|  |  | % of Total | 42.5% | 54.8% | 97.4% |
| Total | | Count | 315 | 407 | 722 |
|  |  | % within Sex | 100.0% | 100.0% | 100.0% |
|  |  | % of Total | 43.6% | 56.4% | 100.0% |

| **Chi-Square Tests** | | | | | |
| --- | --- | --- | --- | --- | --- |
|  | Value | df | Asymptotic Significance (2-sided) | Exact Sig. (2-sided) | Exact Sig. (1-sided) |
| Pearson Chi-Square | .018^a^ | 1 | .892 |  |  |
| Continuity Correction^b^ | .000 | 1 | 1.000 |  |  |
| Likelihood Ratio | .018 | 1 | .892 |  |  |
| Fisher's Exact Test |  |  |  | 1.000 | .543 |
| Linear-by-Linear Association | .018 | 1 | .892 |  |  |
| N of Valid Cases | 722 |  |  |  |  |
| a. 0 cells (0.0%) have expected count less than 5. The minimum expected count is 8.29. | | | | | |
| b. Computed only for a 2x2 table | | | | | |

**Friends/peers * Grade category**

| **Crosstab** | | | | | |
| --- | --- | --- | --- | --- | --- |
|  | | | Grade category | | Total |
|  |  |  | 9-10 | 11-12 |  |
| Friends/peers | Yes | Count | 9 | 10 | 19 |
|  |  | % within Grade category | 2.3% | 3.0% | 2.6% |
|  |  | % of Total | 1.2% | 1.4% | 2.6% |
|  | No | Count | 378 | 325 | 703 |
|  |  | % within Grade category | 97.7% | 97.0% | 97.4% |
|  |  | % of Total | 52.4% | 45.0% | 97.4% |
| Total | | Count | 387 | 335 | 722 |
|  |  | % within Grade category | 100.0% | 100.0% | 100.0% |
|  |  | % of Total | 53.6% | 46.4% | 100.0% |

| **Chi-Square Tests** | | | | | |
| --- | --- | --- | --- | --- | --- |
|  | Value | df | Asymptotic Significance (2-sided) | Exact Sig. (2-sided) | Exact Sig. (1-sided) |
| Pearson Chi-Square | .305^a^ | 1 | .581 |  |  |
| Continuity Correction^b^ | .102 | 1 | .750 |  |  |
| Likelihood Ratio | .304 | 1 | .582 |  |  |
| Fisher's Exact Test |  |  |  | .645 | .374 |
| Linear-by-Linear Association | .304 | 1 | .581 |  |  |
| N of Valid Cases | 722 |  |  |  |  |
| a. 0 cells (0.0%) have expected count less than 5. The minimum expected count is 8.82. | | | | | |
| b. Computed only for a 2x2 table | | | | | |

**Friends/peers * School type**

| **Crosstab** | | | | | |
| --- | --- | --- | --- | --- | --- |
|  | | | School type | | Total |
|  |  |  | Public | Private |  |
| Friends/peers | Yes | Count | 11 | 8 | 19 |
|  |  | % within School type | 2.6% | 2.6% | 2.6% |
|  |  | % of Total | 1.5% | 1.1% | 2.6% |
|  | No | Count | 409 | 294 | 703 |
|  |  | % within School type | 97.4% | 97.4% | 97.4% |
|  |  | % of Total | 56.6% | 40.7% | 97.4% |
| Total | | Count | 420 | 302 | 722 |
|  |  | % within School type | 100.0% | 100.0% | 100.0% |
|  |  | % of Total | 58.2% | 41.8% | 100.0% |

| **Chi-Square Tests** | | | | | |
| --- | --- | --- | --- | --- | --- |
|  | Value | df | Asymptotic Significance (2-sided) | Exact Sig. (2-sided) | Exact Sig. (1-sided) |
| Pearson Chi-Square | .001^a^ | 1 | .980 |  |  |
| Continuity Correction^b^ | .000 | 1 | 1.000 |  |  |
| Likelihood Ratio | .001 | 1 | .980 |  |  |
| Fisher's Exact Test |  |  |  | 1.000 | .578 |
| Linear-by-Linear Association | .001 | 1 | .980 |  |  |
| N of Valid Cases | 722 |  |  |  |  |
| a. 0 cells (0.0%) have expected count less than 5. The minimum expected count is 7.95. | | | | | |
| b. Computed only for a 2x2 table | | | | | |

**Teachers * Sex**

| **Crosstab** | | | | | |
| --- | --- | --- | --- | --- | --- |
|  | | | Sex | | Total |
|  |  |  | Male | Female |  |
| Teachers | Yes | Count | 28 | 50 | 78 |
|  |  | % within Sex | 8.9% | 12.3% | 10.8% |
|  |  | % of Total | 3.9% | 6.9% | 10.8% |
|  | No | Count | 287 | 357 | 644 |
|  |  | % within Sex | 91.1% | 87.7% | 89.2% |
|  |  | % of Total | 39.8% | 49.4% | 89.2% |
| Total | | Count | 315 | 407 | 722 |
|  |  | % within Sex | 100.0% | 100.0% | 100.0% |
|  |  | % of Total | 43.6% | 56.4% | 100.0% |

| **Chi-Square Tests** | | | | | |
| --- | --- | --- | --- | --- | --- |
|  | Value | df | Asymptotic Significance (2-sided) | Exact Sig. (2-sided) | Exact Sig. (1-sided) |
| Pearson Chi-Square | 2.125^a^ | 1 | .145 |  |  |
| Continuity Correction^b^ | 1.788 | 1 | .181 |  |  |
| Likelihood Ratio | 2.159 | 1 | .142 |  |  |
| Fisher's Exact Test |  |  |  | .149 | .090 |
| Linear-by-Linear Association | 2.122 | 1 | .145 |  |  |
| N of Valid Cases | 722 |  |  |  |  |
| a. 0 cells (0.0%) have expected count less than 5. The minimum expected count is 34.03. | | | | | |
| b. Computed only for a 2x2 table | | | | | |

**Teachers * Grade category**

| **Crosstab** | | | | | |
| --- | --- | --- | --- | --- | --- |
|  | | | Grade category | | Total |
|  |  |  | 9-10 | 11-12 |  |
| Teachers | Yes | Count | 45 | 33 | 78 |
|  |  | % within Grade category | 11.6% | 9.9% | 10.8% |
|  |  | % of Total | 6.2% | 4.6% | 10.8% |
|  | No | Count | 342 | 302 | 644 |
|  |  | % within Grade category | 88.4% | 90.1% | 89.2% |
|  |  | % of Total | 47.4% | 41.8% | 89.2% |
| Total | | Count | 387 | 335 | 722 |
|  |  | % within Grade category | 100.0% | 100.0% | 100.0% |
|  |  | % of Total | 53.6% | 46.4% | 100.0% |

| **Chi-Square Tests** | | | | | |
| --- | --- | --- | --- | --- | --- |
|  | Value | df | Asymptotic Significance (2-sided) | Exact Sig. (2-sided) | Exact Sig. (1-sided) |
| Pearson Chi-Square | .589^a^ | 1 | .443 |  |  |
| Continuity Correction^b^ | .419 | 1 | .518 |  |  |
| Likelihood Ratio | .591 | 1 | .442 |  |  |
| Fisher's Exact Test |  |  |  | .472 | .259 |
| Linear-by-Linear Association | .588 | 1 | .443 |  |  |
| N of Valid Cases | 722 |  |  |  |  |
| a. 0 cells (0.0%) have expected count less than 5. The minimum expected count is 36.19. | | | | | |
| b. Computed only for a 2x2 table | | | | | |

**Teachers * School type**

| **Crosstab** | | | | | |
| --- | --- | --- | --- | --- | --- |
|  | | | School type | | Total |
|  |  |  | Public | Private |  |
| Teachers | Yes | Count | 37 | 41 | 78 |
|  |  | % within School type | 8.8% | 13.6% | 10.8% |
|  |  | % of Total | 5.1% | 5.7% | 10.8% |
|  | No | Count | 383 | 261 | 644 |
|  |  | % within School type | 91.2% | 86.4% | 89.2% |
|  |  | % of Total | 53.0% | 36.1% | 89.2% |
| Total | | Count | 420 | 302 | 722 |
|  |  | % within School type | 100.0% | 100.0% | 100.0% |
|  |  | % of Total | 58.2% | 41.8% | 100.0% |

| **Chi-Square Tests** | | | | | |
| --- | --- | --- | --- | --- | --- |
|  | Value | df | Asymptotic Significance (2-sided) | Exact Sig. (2-sided) | Exact Sig. (1-sided) |
| Pearson Chi-Square | 4.142^a^ | 1 | .042 |  |  |
| Continuity Correction^b^ | 3.662 | 1 | .056 |  |  |
| Likelihood Ratio | 4.085 | 1 | .043 |  |  |
| Fisher's Exact Test |  |  |  | .051 | .028 |
| Linear-by-Linear Association | 4.137 | 1 | .042 |  |  |
| N of Valid Cases | 722 |  |  |  |  |
| a. 0 cells (0.0%) have expected count less than 5. The minimum expected count is 32.63. | | | | | |
| b. Computed only for a 2x2 table | | | | | |

**Health clubs * Sex**

| **Crosstab** | | | | | |
| --- | --- | --- | --- | --- | --- |
|  | | | Sex | | Total |
|  |  |  | Male | Female |  |
| Health clubs | Yes | Count | 12 | 26 | 38 |
|  |  | % within Sex | 3.8% | 6.4% | 5.3% |
|  |  | % of Total | 1.7% | 3.6% | 5.3% |
|  | No | Count | 303 | 381 | 684 |
|  |  | % within Sex | 96.2% | 93.6% | 94.7% |
|  |  | % of Total | 42.0% | 52.8% | 94.7% |
| Total | | Count | 315 | 407 | 722 |
|  |  | % within Sex | 100.0% | 100.0% | 100.0% |
|  |  | % of Total | 43.6% | 56.4% | 100.0% |

| **Chi-Square Tests** | | | | | |
| --- | --- | --- | --- | --- | --- |
|  | Value | df | Asymptotic Significance (2-sided) | Exact Sig. (2-sided) | Exact Sig. (1-sided) |
| Pearson Chi-Square | 2.368^a^ | 1 | .124 |  |  |
| Continuity Correction^b^ | 1.879 | 1 | .170 |  |  |
| Likelihood Ratio | 2.441 | 1 | .118 |  |  |
| Fisher's Exact Test |  |  |  | .134 | .084 |
| Linear-by-Linear Association | 2.365 | 1 | .124 |  |  |
| N of Valid Cases | 722 |  |  |  |  |
| a. 0 cells (0.0%) have expected count less than 5. The minimum expected count is 16.58. | | | | | |
| b. Computed only for a 2x2 table | | | | | |

**Health clubs * Grade category**

| **Crosstab** | | | | | |
| --- | --- | --- | --- | --- | --- |
|  | | | Grade category | | Total |
|  |  |  | 9-10 | 11-12 |  |
| Health clubs | Yes | Count | 20 | 18 | 38 |
|  |  | % within Grade category | 5.2% | 5.4% | 5.3% |
|  |  | % of Total | 2.8% | 2.5% | 5.3% |
|  | No | Count | 367 | 317 | 684 |
|  |  | % within Grade category | 94.8% | 94.6% | 94.7% |
|  |  | % of Total | 50.8% | 43.9% | 94.7% |
| Total | | Count | 387 | 335 | 722 |
|  |  | % within Grade category | 100.0% | 100.0% | 100.0% |
|  |  | % of Total | 53.6% | 46.4% | 100.0% |

| **Chi-Square Tests** | | | | | |
| --- | --- | --- | --- | --- | --- |
|  | Value | df | Asymptotic Significance (2-sided) | Exact Sig. (2-sided) | Exact Sig. (1-sided) |
| Pearson Chi-Square | .015^a^ | 1 | .902 |  |  |
| Continuity Correction^b^ | .000 | 1 | 1.000 |  |  |
| Likelihood Ratio | .015 | 1 | .902 |  |  |
| Fisher's Exact Test |  |  |  | 1.000 | .516 |
| Linear-by-Linear Association | .015 | 1 | .902 |  |  |
| N of Valid Cases | 722 |  |  |  |  |
| a. 0 cells (0.0%) have expected count less than 5. The minimum expected count is 17.63. | | | | | |
| b. Computed only for a 2x2 table | | | | | |

**Health clubs * School type**

| **Crosstab** | | | | | |
| --- | --- | --- | --- | --- | --- |
|  | | | School type | | Total |
|  |  |  | Public | Private |  |
| Health clubs | Yes | Count | 22 | 16 | 38 |
|  |  | % within School type | 5.2% | 5.3% | 5.3% |
|  |  | % of Total | 3.0% | 2.2% | 5.3% |
|  | No | Count | 398 | 286 | 684 |
|  |  | % within School type | 94.8% | 94.7% | 94.7% |
|  |  | % of Total | 55.1% | 39.6% | 94.7% |
| Total | | Count | 420 | 302 | 722 |
|  |  | % within School type | 100.0% | 100.0% | 100.0% |
|  |  | % of Total | 58.2% | 41.8% | 100.0% |

| **Chi-Square Tests** | | | | | |
| --- | --- | --- | --- | --- | --- |
|  | Value | df | Asymptotic Significance (2-sided) | Exact Sig. (2-sided) | Exact Sig. (1-sided) |
| Pearson Chi-Square | .001^a^ | 1 | .972 |  |  |
| Continuity Correction^b^ | .000 | 1 | 1.000 |  |  |
| Likelihood Ratio | .001 | 1 | .972 |  |  |
| Fisher's Exact Test |  |  |  | 1.000 | .550 |
| Linear-by-Linear Association | .001 | 1 | .972 |  |  |
| N of Valid Cases | 722 |  |  |  |  |
| a. 0 cells (0.0%) have expected count less than 5. The minimum expected count is 15.89. | | | | | |
| b. Computed only for a 2x2 table | | | | | |

**Religious leaders * Sex**

| **Crosstab** | | | | | |
| --- | --- | --- | --- | --- | --- |
|  | | | Sex | | Total |
|  |  |  | Male | Female |  |
| Religious leaders | Yes | Count | 35 | 57 | 92 |
|  |  | % within Sex | 11.1% | 14.0% | 12.7% |
|  |  | % of Total | 4.8% | 7.9% | 12.7% |
|  | No | Count | 280 | 350 | 630 |
|  |  | % within Sex | 88.9% | 86.0% | 87.3% |
|  |  | % of Total | 38.8% | 48.5% | 87.3% |
| Total | | Count | 315 | 407 | 722 |
|  |  | % within Sex | 100.0% | 100.0% | 100.0% |
|  |  | % of Total | 43.6% | 56.4% | 100.0% |

| **Chi-Square Tests** | | | | | |
| --- | --- | --- | --- | --- | --- |
|  | Value | df | Asymptotic Significance (2-sided) | Exact Sig. (2-sided) | Exact Sig. (1-sided) |
| Pearson Chi-Square | 1.337^a^ | 1 | .247 |  |  |
| Continuity Correction^b^ | 1.090 | 1 | .297 |  |  |
| Likelihood Ratio | 1.351 | 1 | .245 |  |  |
| Fisher's Exact Test |  |  |  | .262 | .148 |
| Linear-by-Linear Association | 1.336 | 1 | .248 |  |  |
| N of Valid Cases | 722 |  |  |  |  |
| a. 0 cells (0.0%) have expected count less than 5. The minimum expected count is 40.14. | | | | | |
| b. Computed only for a 2x2 table | | | | | |

**Religious leaders * Grade category**

| **Crosstab** | | | | | |
| --- | --- | --- | --- | --- | --- |
|  | | | Grade category | | Total |
|  |  |  | 9-10 | 11-12 |  |
| Religious leaders | Yes | Count | 50 | 42 | 92 |
|  |  | % within Grade category | 12.9% | 12.5% | 12.7% |
|  |  | % of Total | 6.9% | 5.8% | 12.7% |
|  | No | Count | 337 | 293 | 630 |
|  |  | % within Grade category | 87.1% | 87.5% | 87.3% |
|  |  | % of Total | 46.7% | 40.6% | 87.3% |
| Total | | Count | 387 | 335 | 722 |
|  |  | % within Grade category | 100.0% | 100.0% | 100.0% |
|  |  | % of Total | 53.6% | 46.4% | 100.0% |

| **Chi-Square Tests** | | | | | |
| --- | --- | --- | --- | --- | --- |
|  | Value | df | Asymptotic Significance (2-sided) | Exact Sig. (2-sided) | Exact Sig. (1-sided) |
| Pearson Chi-Square | .024^a^ | 1 | .878 |  |  |
| Continuity Correction^b^ | .002 | 1 | .967 |  |  |
| Likelihood Ratio | .024 | 1 | .878 |  |  |
| Fisher's Exact Test |  |  |  | .911 | .484 |
| Linear-by-Linear Association | .024 | 1 | .878 |  |  |
| N of Valid Cases | 722 |  |  |  |  |
| a. 0 cells (0.0%) have expected count less than 5. The minimum expected count is 42.69. | | | | | |
| b. Computed only for a 2x2 table | | | | | |

**Religious leaders * School type**

| **Crosstab** | | | | | |
| --- | --- | --- | --- | --- | --- |
|  | | | School type | | Total |
|  |  |  | Public | Private |  |
| Religious leaders | Yes | Count | 54 | 38 | 92 |
|  |  | % within School type | 12.9% | 12.6% | 12.7% |
|  |  | % of Total | 7.5% | 5.3% | 12.7% |
|  | No | Count | 366 | 264 | 630 |
|  |  | % within School type | 87.1% | 87.4% | 87.3% |
|  |  | % of Total | 50.7% | 36.6% | 87.3% |
| Total | | Count | 420 | 302 | 722 |
|  |  | % within School type | 100.0% | 100.0% | 100.0% |
|  |  | % of Total | 58.2% | 41.8% | 100.0% |

| **Chi-Square Tests** | | | | | |
| --- | --- | --- | --- | --- | --- |
|  | Value | df | Asymptotic Significance (2-sided) | Exact Sig. (2-sided) | Exact Sig. (1-sided) |
| Pearson Chi-Square | .012^a^ | 1 | .913 |  |  |
| Continuity Correction^b^ | .000 | 1 | 1.000 |  |  |
| Likelihood Ratio | .012 | 1 | .913 |  |  |
| Fisher's Exact Test |  |  |  | 1.000 | .503 |
| Linear-by-Linear Association | .012 | 1 | .913 |  |  |
| N of Valid Cases | 722 |  |  |  |  |
| a. 0 cells (0.0%) have expected count less than 5. The minimum expected count is 38.48. | | | | | |
| b. Computed only for a 2x2 table | | | | | |

**13. Issues or topics of great concern among adolescents:** What health topics do you think are major concerns requiring attention among adolescents?

**Sexual and reproductive health issues (risky sexual behaviors) * Sex**

| **Crosstab** | | | | | |
| --- | --- | --- | --- | --- | --- |
|  | | | Sex | | Total |
|  |  |  | Male | Female |  |
| Sexual and reproductive health issues (risky sexual behaviors) | Yes | Count | 181 | 267 | 448 |
|  |  | % within Sex | 58.0% | 65.6% | 62.3% |
|  |  | % of Total | 25.2% | 37.1% | 62.3% |
|  | No | Count | 131 | 140 | 271 |
|  |  | % within Sex | 42.0% | 34.4% | 37.7% |
|  |  | % of Total | 18.2% | 19.5% | 37.7% |
| Total | | Count | 312 | 407 | 719 |
|  |  | % within Sex | 100.0% | 100.0% | 100.0% |
|  |  | % of Total | 43.4% | 56.6% | 100.0% |

| **Chi-Square Tests** | | | | | |
| --- | --- | --- | --- | --- | --- |
|  | Value | df | Asymptotic Significance (2-sided) | Exact Sig. (2-sided) | Exact Sig. (1-sided) |
| Pearson Chi-Square | 4.331^a^ | 1 | .037 |  |  |
| Continuity Correction^b^ | 4.014 | 1 | .045 |  |  |
| Likelihood Ratio | 4.322 | 1 | .038 |  |  |
| Fisher's Exact Test |  |  |  | .043 | .023 |
| Linear-by-Linear Association | 4.325 | 1 | .038 |  |  |
| N of Valid Cases | 719 |  |  |  |  |
| a. 0 cells (0.0%) have expected count less than 5. The minimum expected count is 117.60. | | | | | |
| b. Computed only for a 2x2 table | | | | | |

**Sexual and reproductive health issues (risky sexual behaviors) * Grade category**

| **Crosstab** | | | | | |
| --- | --- | --- | --- | --- | --- |
|  | | | Grade category | | Total |
|  |  |  | 9-10 | 11-12 |  |
| Sexual and reproductive health issues (risky sexual behaviors) | Yes | Count | 247 | 201 | 448 |
|  |  | % within Grade category | 64.0% | 60.4% | 62.3% |
|  |  | % of Total | 34.4% | 28.0% | 62.3% |
|  | No | Count | 139 | 132 | 271 |
|  |  | % within Grade category | 36.0% | 39.6% | 37.7% |
|  |  | % of Total | 19.3% | 18.4% | 37.7% |
| Total | | Count | 386 | 333 | 719 |
|  |  | % within Grade category | 100.0% | 100.0% | 100.0% |
|  |  | % of Total | 53.7% | 46.3% | 100.0% |

| **Chi-Square Tests** | | | | | |
| --- | --- | --- | --- | --- | --- |
|  | Value | df | Asymptotic Significance (2-sided) | Exact Sig. (2-sided) | Exact Sig. (1-sided) |
| Pearson Chi-Square | 1.003^a^ | 1 | .317 |  |  |
| Continuity Correction^b^ | .854 | 1 | .355 |  |  |
| Likelihood Ratio | 1.002 | 1 | .317 |  |  |
| Fisher's Exact Test |  |  |  | .354 | .178 |
| Linear-by-Linear Association | 1.001 | 1 | .317 |  |  |
| N of Valid Cases | 719 |  |  |  |  |
| a. 0 cells (0.0%) have expected count less than 5. The minimum expected count is 125.51. | | | | | |
| b. Computed only for a 2x2 table | | | | | |

**Sexual and reproductive health issues (risky sexual behaviors) * School type**

| **Crosstab** | | | | | |
| --- | --- | --- | --- | --- | --- |
|  | | | School type | | Total |
|  |  |  | Public | Private |  |
| Sexual and reproductive health issues (risky sexual behaviors) | Yes | Count | 262 | 186 | 448 |
|  |  | % within School type | 62.7% | 61.8% | 62.3% |
|  |  | % of Total | 36.4% | 25.9% | 62.3% |
|  | No | Count | 156 | 115 | 271 |
|  |  | % within School type | 37.3% | 38.2% | 37.7% |
|  |  | % of Total | 21.7% | 16.0% | 37.7% |
| Total | | Count | 418 | 301 | 719 |
|  |  | % within School type | 100.0% | 100.0% | 100.0% |
|  |  | % of Total | 58.1% | 41.9% | 100.0% |

| **Chi-Square Tests** | | | | | |
| --- | --- | --- | --- | --- | --- |
|  | Value | df | Asymptotic Significance (2-sided) | Exact Sig. (2-sided) | Exact Sig. (1-sided) |
| Pearson Chi-Square | .058^a^ | 1 | .809 |  |  |
| Continuity Correction^b^ | .027 | 1 | .870 |  |  |
| Likelihood Ratio | .058 | 1 | .809 |  |  |
| Fisher's Exact Test |  |  |  | .815 | .435 |
| Linear-by-Linear Association | .058 | 1 | .809 |  |  |
| N of Valid Cases | 719 |  |  |  |  |
| a. 0 cells (0.0%) have expected count less than 5. The minimum expected count is 113.45. | | | | | |
| b. Computed only for a 2x2 table | | | | | |

**Mental health issues * Sex**

| **Crosstab** | | | | | |
| --- | --- | --- | --- | --- | --- |
|  | | | Sex | | Total |
|  |  |  | Male | Female |  |
| Mental health issues | Yes | Count | 140 | 215 | 355 |
|  |  | % within Sex | 44.7% | 53.0% | 49.4% |
|  |  | % of Total | 19.5% | 29.9% | 49.4% |
|  | No | Count | 173 | 191 | 364 |
|  |  | % within Sex | 55.3% | 47.0% | 50.6% |
|  |  | % of Total | 24.1% | 26.6% | 50.6% |
| Total | | Count | 313 | 406 | 719 |
|  |  | % within Sex | 100.0% | 100.0% | 100.0% |
|  |  | % of Total | 43.5% | 56.5% | 100.0% |

| **Chi-Square Tests** | | | | | |
| --- | --- | --- | --- | --- | --- |
|  | Value | df | Asymptotic Significance (2-sided) | Exact Sig. (2-sided) | Exact Sig. (1-sided) |
| Pearson Chi-Square | 4.786^a^ | 1 | .029 |  |  |
| Continuity Correction^b^ | 4.463 | 1 | .035 |  |  |
| Likelihood Ratio | 4.793 | 1 | .029 |  |  |
| Fisher's Exact Test |  |  |  | .029 | .017 |
| Linear-by-Linear Association | 4.779 | 1 | .029 |  |  |
| N of Valid Cases | 719 |  |  |  |  |
| a. 0 cells (0.0%) have expected count less than 5. The minimum expected count is 154.54. | | | | | |
| b. Computed only for a 2x2 table | | | | | |

**Mental health issues * Grade category**

| **Crosstab** | | | | | |
| --- | --- | --- | --- | --- | --- |
|  | | | Grade category | | Total |
|  |  |  | 9-10 | 11-12 |  |
| Mental health issues | Yes | Count | 183 | 172 | 355 |
|  |  | % within Grade category | 47.4% | 51.7% | 49.4% |
|  |  | % of Total | 25.5% | 23.9% | 49.4% |
|  | No | Count | 203 | 161 | 364 |
|  |  | % within Grade category | 52.6% | 48.3% | 50.6% |
|  |  | % of Total | 28.2% | 22.4% | 50.6% |
| Total | | Count | 386 | 333 | 719 |
|  |  | % within Grade category | 100.0% | 100.0% | 100.0% |
|  |  | % of Total | 53.7% | 46.3% | 100.0% |

| **Chi-Square Tests** | | | | | |
| --- | --- | --- | --- | --- | --- |
|  | Value | df | Asymptotic Significance (2-sided) | Exact Sig. (2-sided) | Exact Sig. (1-sided) |
| Pearson Chi-Square | 1.287^a^ | 1 | .257 |  |  |
| Continuity Correction^b^ | 1.123 | 1 | .289 |  |  |
| Likelihood Ratio | 1.288 | 1 | .257 |  |  |
| Fisher's Exact Test |  |  |  | .263 | .145 |
| Linear-by-Linear Association | 1.285 | 1 | .257 |  |  |
| N of Valid Cases | 719 |  |  |  |  |
| a. 0 cells (0.0%) have expected count less than 5. The minimum expected count is 164.42. | | | | | |
| b. Computed only for a 2x2 table | | | | | |

**Mental health issues * School type**

| **Crosstab** | | | | | |
| --- | --- | --- | --- | --- | --- |
|  | | | School type | | Total |
|  |  |  | Public | Private |  |
| Mental health issues | Yes | Count | 208 | 147 | 355 |
|  |  | % within School type | 49.9% | 48.7% | 49.4% |
|  |  | % of Total | 28.9% | 20.4% | 49.4% |
|  | No | Count | 209 | 155 | 364 |
|  |  | % within School type | 50.1% | 51.3% | 50.6% |
|  |  | % of Total | 29.1% | 21.6% | 50.6% |
| Total | | Count | 417 | 302 | 719 |
|  |  | % within School type | 100.0% | 100.0% | 100.0% |
|  |  | % of Total | 58.0% | 42.0% | 100.0% |

| **Chi-Square Tests** | | | | | |
| --- | --- | --- | --- | --- | --- |
|  | Value | df | Asymptotic Significance (2-sided) | Exact Sig. (2-sided) | Exact Sig. (1-sided) |
| Pearson Chi-Square | .102^a^ | 1 | .750 |  |  |
| Continuity Correction^b^ | .059 | 1 | .808 |  |  |
| Likelihood Ratio | .102 | 1 | .750 |  |  |
| Fisher's Exact Test |  |  |  | .763 | .404 |
| Linear-by-Linear Association | .102 | 1 | .750 |  |  |
| N of Valid Cases | 719 |  |  |  |  |
| a. 0 cells (0.0%) have expected count less than 5. The minimum expected count is 149.11. | | | | | |
| b. Computed only for a 2x2 table | | | | | |

**Social media addiction * Sex**

| **Crosstab** | | | | | |
| --- | --- | --- | --- | --- | --- |
|  | | | Sex | | Total |
|  |  |  | Male | Female |  |
| Social media addiction | Yes | Count | 162 | 175 | 337 |
|  |  | % within Sex | 51.8% | 43.0% | 46.8% |
|  |  | % of Total | 22.5% | 24.3% | 46.8% |
|  | No | Count | 151 | 232 | 383 |
|  |  | % within Sex | 48.2% | 57.0% | 53.2% |
|  |  | % of Total | 21.0% | 32.2% | 53.2% |
| Total | | Count | 313 | 407 | 720 |
|  |  | % within Sex | 100.0% | 100.0% | 100.0% |
|  |  | % of Total | 43.5% | 56.5% | 100.0% |

| **Chi-Square Tests** | | | | | |
| --- | --- | --- | --- | --- | --- |
|  | Value | df | Asymptotic Significance (2-sided) | Exact Sig. (2-sided) | Exact Sig. (1-sided) |
| Pearson Chi-Square | 5.453^a^ | 1 | .020 |  |  |
| Continuity Correction^b^ | 5.107 | 1 | .024 |  |  |
| Likelihood Ratio | 5.455 | 1 | .020 |  |  |
| Fisher's Exact Test |  |  |  | .024 | .012 |
| Linear-by-Linear Association | 5.445 | 1 | .020 |  |  |
| N of Valid Cases | 720 |  |  |  |  |
| a. 0 cells (0.0%) have expected count less than 5. The minimum expected count is 146.50. | | | | | |
| b. Computed only for a 2x2 table | | | | | |

**Social media addiction * Grade category**

| **Crosstab** | | | | | |
| --- | --- | --- | --- | --- | --- |
|  | | | Grade category | | Total |
|  |  |  | 9-10 | 11-12 |  |
| Social media addiction | Yes | Count | 173 | 164 | 337 |
|  |  | % within Grade category | 44.7% | 49.2% | 46.8% |
|  |  | % of Total | 24.0% | 22.8% | 46.8% |
|  | No | Count | 214 | 169 | 383 |
|  |  | % within Grade category | 55.3% | 50.8% | 53.2% |
|  |  | % of Total | 29.7% | 23.5% | 53.2% |
| Total | | Count | 387 | 333 | 720 |
|  |  | % within Grade category | 100.0% | 100.0% | 100.0% |
|  |  | % of Total | 53.8% | 46.3% | 100.0% |

| **Chi-Square Tests** | | | | | |
| --- | --- | --- | --- | --- | --- |
|  | Value | df | Asymptotic Significance (2-sided) | Exact Sig. (2-sided) | Exact Sig. (1-sided) |
| Pearson Chi-Square | 1.486^a^ | 1 | .223 |  |  |
| Continuity Correction^b^ | 1.309 | 1 | .253 |  |  |
| Likelihood Ratio | 1.486 | 1 | .223 |  |  |
| Fisher's Exact Test |  |  |  | .231 | .126 |
| Linear-by-Linear Association | 1.484 | 1 | .223 |  |  |
| N of Valid Cases | 720 |  |  |  |  |
| a. 0 cells (0.0%) have expected count less than 5. The minimum expected count is 155.86. | | | | | |
| b. Computed only for a 2x2 table | | | | | |

**Social media addiction * School type**

| **Crosstab** | | | | | |
| --- | --- | --- | --- | --- | --- |
|  | | | School type | | Total |
|  |  |  | Public | Private |  |
| Social media addiction | Yes | Count | 183 | 154 | 337 |
|  |  | % within School type | 43.8% | 51.0% | 46.8% |
|  |  | % of Total | 25.4% | 21.4% | 46.8% |
|  | No | Count | 235 | 148 | 383 |
|  |  | % within School type | 56.2% | 49.0% | 53.2% |
|  |  | % of Total | 32.6% | 20.6% | 53.2% |
| Total | | Count | 418 | 302 | 720 |
|  |  | % within School type | 100.0% | 100.0% | 100.0% |
|  |  | % of Total | 58.1% | 41.9% | 100.0% |

| **Chi-Square Tests** | | | | | |
| --- | --- | --- | --- | --- | --- |
|  | Value | df | Asymptotic Significance (2-sided) | Exact Sig. (2-sided) | Exact Sig. (1-sided) |
| Pearson Chi-Square | 3.664^a^ | 1 | .056 |  |  |
| Continuity Correction^b^ | 3.380 | 1 | .066 |  |  |
| Likelihood Ratio | 3.664 | 1 | .056 |  |  |
| Fisher's Exact Test |  |  |  | .059 | .033 |
| Linear-by-Linear Association | 3.659 | 1 | .056 |  |  |
| N of Valid Cases | 720 |  |  |  |  |
| a. 0 cells (0.0%) have expected count less than 5. The minimum expected count is 141.35. | | | | | |
| b. Computed only for a 2x2 table | | | | | |

**Substance abuse issues (including cigarette, chat, alcohol, ganja, and others) * Sex**

| **Crosstab** | | | | | |
| --- | --- | --- | --- | --- | --- |
|  | | | Sex | | Total |
|  |  |  | Male | Female |  |
| Substance abuse issues (including cigarette, chat, alcohol, and others) | Yes | Count | 218 | 181 | 399 |
|  |  | % within Sex | 69.4% | 44.5% | 55.3% |
|  |  | % of Total | 30.2% | 25.1% | 55.3% |
|  | No | Count | 96 | 226 | 322 |
|  |  | % within Sex | 30.6% | 55.5% | 44.7% |
|  |  | % of Total | 13.3% | 31.3% | 44.7% |
| Total | | Count | 314 | 407 | 721 |
|  |  | % within Sex | 100.0% | 100.0% | 100.0% |
|  |  | % of Total | 43.6% | 56.4% | 100.0% |

| **Chi-Square Tests** | | | | | |
| --- | --- | --- | --- | --- | --- |
|  | Value | df | Asymptotic Significance (2-sided) | Exact Sig. (2-sided) | Exact Sig. (1-sided) |
| Pearson Chi-Square | 44.663^a^ | 1 | .000 |  |  |
| Continuity Correction^b^ | 43.659 | 1 | .000 |  |  |
| Likelihood Ratio | 45.419 | 1 | .000 |  |  |
| Fisher's Exact Test |  |  |  | .000 | .000 |
| Linear-by-Linear Association | 44.601 | 1 | .000 |  |  |
| N of Valid Cases | 721 |  |  |  |  |
| a. 0 cells (0.0%) have expected count less than 5. The minimum expected count is 140.23. | | | | | |
| b. Computed only for a 2x2 table | | | | | |

**Substance abuse issues (including cigarette, chat, alcohol, ganja, and others) * Grade category**

| **Crosstab** | | | | | |
| --- | --- | --- | --- | --- | --- |
|  | | | Grade category | | Total |
|  |  |  | 9-10 | 11-12 |  |
| Substance abuse issues (including cigarette, chat, alcohol, and others) | Yes | Count | 205 | 194 | 399 |
|  |  | % within Grade category | 53.0% | 58.1% | 55.3% |
|  |  | % of Total | 28.4% | 26.9% | 55.3% |
|  | No | Count | 182 | 140 | 322 |
|  |  | % within Grade category | 47.0% | 41.9% | 44.7% |
|  |  | % of Total | 25.2% | 19.4% | 44.7% |
| Total | | Count | 387 | 334 | 721 |
|  |  | % within Grade category | 100.0% | 100.0% | 100.0% |
|  |  | % of Total | 53.7% | 46.3% | 100.0% |

| **Chi-Square Tests** | | | | | |
| --- | --- | --- | --- | --- | --- |
|  | Value | df | Asymptotic Significance (2-sided) | Exact Sig. (2-sided) | Exact Sig. (1-sided) |
| Pearson Chi-Square | 1.896^a^ | 1 | .169 |  |  |
| Continuity Correction^b^ | 1.695 | 1 | .193 |  |  |
| Likelihood Ratio | 1.898 | 1 | .168 |  |  |
| Fisher's Exact Test |  |  |  | .177 | .096 |
| Linear-by-Linear Association | 1.893 | 1 | .169 |  |  |
| N of Valid Cases | 721 |  |  |  |  |
| a. 0 cells (0.0%) have expected count less than 5. The minimum expected count is 149.17. | | | | | |
| b. Computed only for a 2x2 table | | | | | |

**Substance abuse issues (including cigarette, chat, alcohol, ganja, and others) * School type**

| **Crosstab** | | | | | |
| --- | --- | --- | --- | --- | --- |
|  | | | School type | | Total |
|  |  |  | Public | Private |  |
| Substance abuse issues (including cigarette, chat, alcohol, and others) | Yes | Count | 234 | 165 | 399 |
|  |  | % within School type | 55.8% | 54.6% | 55.3% |
|  |  | % of Total | 32.5% | 22.9% | 55.3% |
|  | No | Count | 185 | 137 | 322 |
|  |  | % within School type | 44.2% | 45.4% | 44.7% |
|  |  | % of Total | 25.7% | 19.0% | 44.7% |
| Total | | Count | 419 | 302 | 721 |
|  |  | % within School type | 100.0% | 100.0% | 100.0% |
|  |  | % of Total | 58.1% | 41.9% | 100.0% |

| **Chi-Square Tests** | | | | | |
| --- | --- | --- | --- | --- | --- |
|  | Value | df | Asymptotic Significance (2-sided) | Exact Sig. (2-sided) | Exact Sig. (1-sided) |
| Pearson Chi-Square | .104^a^ | 1 | .747 |  |  |
| Continuity Correction^b^ | .061 | 1 | .805 |  |  |
| Likelihood Ratio | .104 | 1 | .747 |  |  |
| Fisher's Exact Test |  |  |  | .762 | .402 |
| Linear-by-Linear Association | .104 | 1 | .747 |  |  |
| N of Valid Cases | 721 |  |  |  |  |
| a. 0 cells (0.0%) have expected count less than 5. The minimum expected count is 134.87. | | | | | |
| b. Computed only for a 2x2 table | | | | | |
